# Synthesis of lamellarin alkaloids using orthoester-masked α-keto acids†
†Electronic supplementary information (ESI) available: Synthetic procedures, compounds' characterisation data and NMR spectra. See DOI: 10.1039/c8sc05678a


**DOI:** 10.1039/c8sc05678a

**Published:** 2019-03-19

**Authors:** Harry J. Shirley, Maria Koyioni, Filip Muncan, Timothy J. Donohoe

**Affiliations:** a Department of Chemistry , University of Oxford , Chemistry Research Laboratory , Mansfield Road , Oxford , OX1 3TA , UK . Email: timothy.donohoe@chem.ox.ac.uk

## Abstract

Pyruvic acid and other α-keto acids are frequently encountered as intermediates in metabolic pathways, yet their application in total synthesis has met with limited success. In this work, we present a bioinspired strategy that utilizes highly functionalized OBO (oxabicyclo[2.2.2]octyl) orthoester masked α-ketoacids as key intermediates for the construction of both type I and II lamellarin alkaloids. Lamellarin D was synthesized, *via* a key 1,4-dicarbonyl, in 7 steps and 22% yield from pyruvic acid. Key steps in the synthesis involve one-pot double enolate functionalisation of **1** followed by double annulation to form the target pyrrole/*N*-vinyl pyrrole core and late-stage direct C–H arylation. Lastly, a novel OBO-masked β-cyano ketone, synthesized from **1**, proved to be a valuable intermediate for construction of the type II lamellarin core *via* HBr-mediated cyclisation. In this way, lamellarin Q was synthesized in 7 steps and 20% yield from pyruvic acid.

## Abstract 2

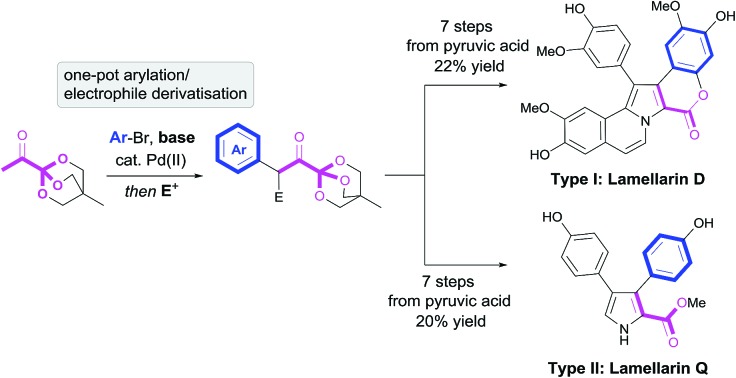
Enolate arylation of a protected pyruvate is used as a key step in the short and efficient syntheses of the lamellarins.

## Introduction

**Scheme 1:**
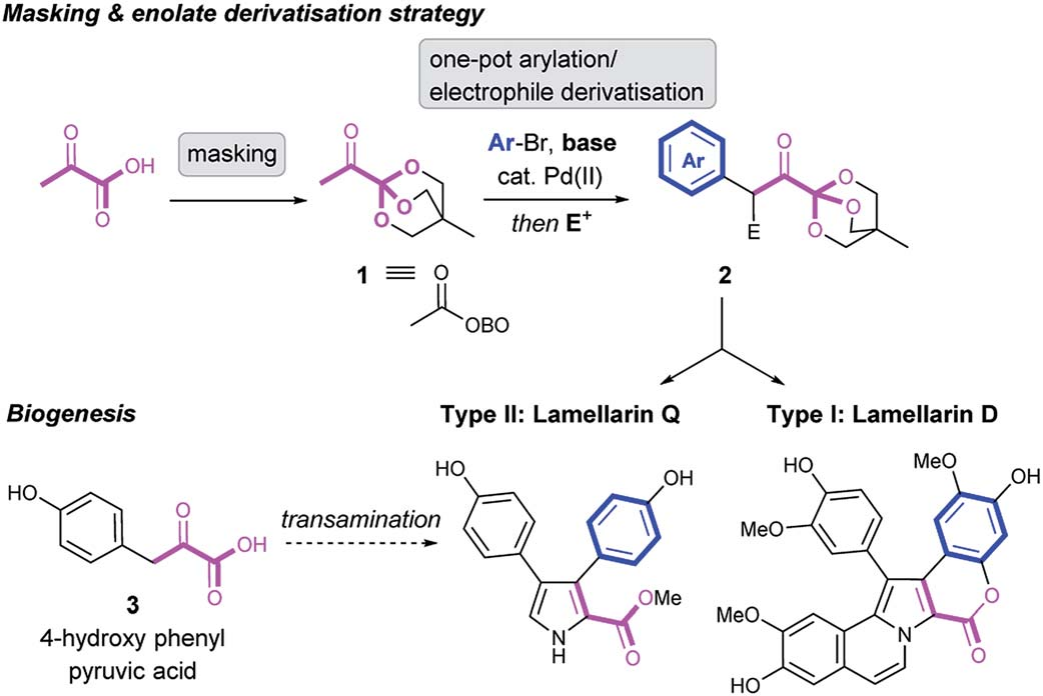
Masked pyruvic acid route to lamellarins D and Q.

The lamellarins are an important family of pyrrole alkaloids isolated from marine organisms, consisting of around 70 distinct members which are categorised into *type I*: pentacyclic skeleton with a fully decorated pyrrole core, or *type II*: monocyclic skeleton with a 3,4-disubstituted pyrrole core (Scheme 1).^1^ The biological activities of this family has proven to be nothing short of remarkable, with lamellarins D, G, H, L and N displaying striking biological assay results.^2^ Without doubt, lamellarin D is the lead compound from this catalogue and its activity has been broadly studied, notably being revealed as a potent cytotoxin and topoisomerase I inhibitor. Many lamellarins have displayed biological effects on both mammalian cells and viruses, including antiproliferative and multidrug resistance reversal activity, cytotoxicity, anti-tumour activity and inhibition of HIV-1 integrase. Given these striking biological properties coupled with their scarcity in the fragile marine environment, there has been intense interest in achieving their total syntheses in order to assist biological assays and SAR studies.^1^ In general, routes to the lamellarins have involved functionalisation of a simple pyrrole core through halogenations and conventional C–C cross couplings or synthesis of a functionalised pyrrole core from acyclic precursors.^3^ Recently, impressive and efficient syntheses of lamellarin D and related alkaloids, including work by Jia (2011),^4^ Chandrasekhar (2017),^5^ and Yang (2017),6*a* have been reported.^6^

Pyruvic acid and its α-keto acid derivatives are ubiquitous in Nature, proven as key intermediates in multiple primary and secondary metabolic pathways. They are involved in the formation of amino acids and in the biogenesis of countless secondary metabolites.^7^ Indeed, the lamellarins are proposed to be biogenetically constructed from transamination of 4-hydroxyphenyl pyruvic acid **3**, possibly to the amino acid tyrosine, en-route to the type II core, which is itself the biogenetic precursor to the type I core (Scheme 1).^8^ Despite the prevalence of α-keto acids in biogenetic pathways, their use in the total synthesis of natural products has had limited success, partly attributed to their instability under basic conditions. This is exemplified in early work by Steglich, who described low yielding routes to lamellarins L^9^ and G trimethyl ether^10^ using pyruvate esters as synthetic starting materials.

Previously, we have demonstrated the synthetic utility of OBO-ester (oxabicyclo[2.2.2]octyl orthoester) masked α-keto acid **1** in the construction of 1,4- and 1,5-dicarbonyls, *via* a Pd-catalysed enolate arylation/alkylation sequence, and subsequent condensation to form aromatic heterocycles, *e.g.* 2-carboxy-pyrroles.^11^ Inspired by the key role of α-keto acids in Nature, we envisioned that appropriately substituted OBO-ester α-keto acids **2** could serve as powerful building blocks with which to prepare the lamellarin alkaloids. Herein, access to highly functionalised masked pyruvic acid derivatives *via* one-pot enolate arylation and alkylation (*e.g.***1** → **2**) was achieved. Subsequent condensation reactions to a pyrrole nucleus under acidic conditions allowed efficient syntheses of both type I and II lamellarin cores. This bioinspired strategy allowed short and high yielding syntheses of lamellarin D (type I) and lamellarin Q (type II) (Scheme 1). Key advances in the synthesis of the type I core include double annulation of a 1,4-dicarbonyl with aminoacetaldehyde diethyl acetal and late-stage C–H arylation of the pyrrole core to complete the type I core. Moreover, S_N_Ar chemistry is used on more than one occasion to allow efficient syntheses of protected phenols from readily available aryl fluorides.

## Results and discussion

### Lamellarin D

**Scheme 2:**
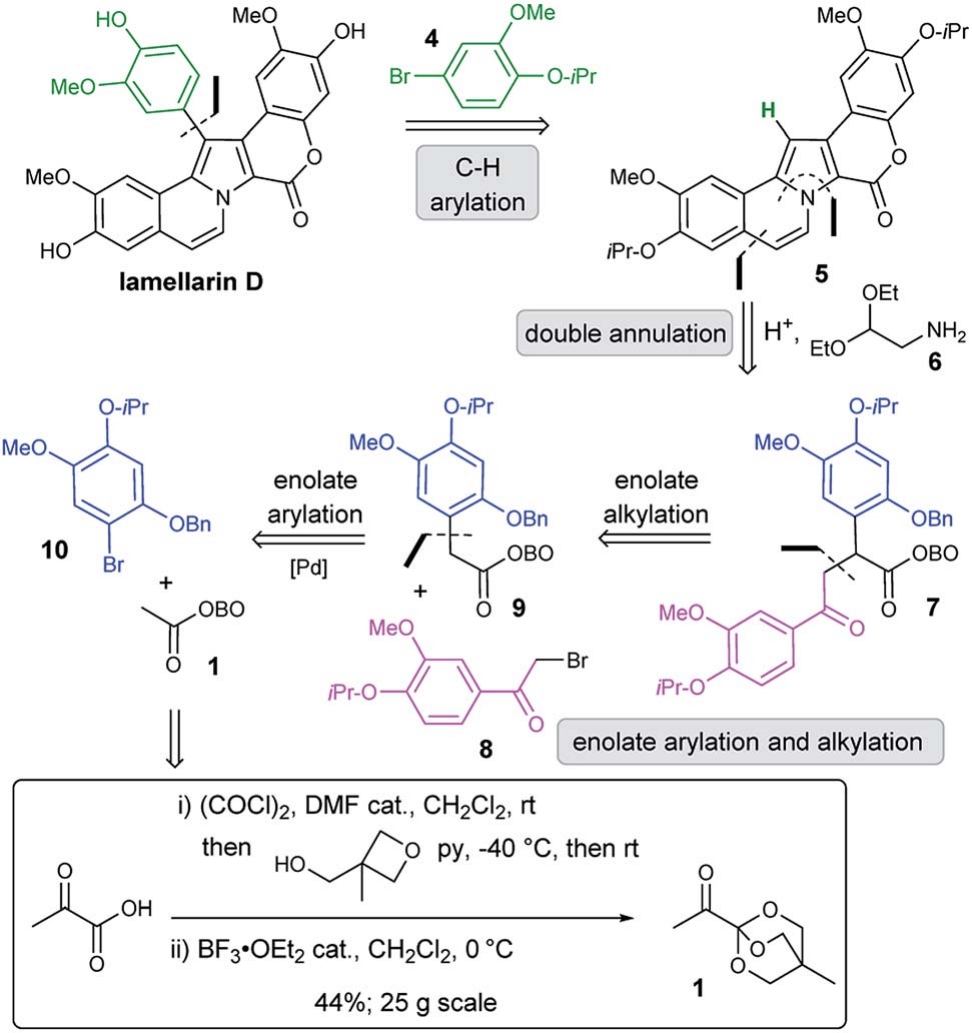
Retrosynthetic analysis of lamellarin D.

Our retrosynthetic analysis of lamellarin D commenced with a key disconnection of the C-4 aryl group, which would correspond to C–H arylation of pyrrole **5** with aryl bromide **4** using transition metal catalysis (Scheme 2). We hypothesised that both the pyrrole and the fused benzenoid ring could be prepared from a key 1,4-dicarbonyl precursor **7** by reaction with a suitably substituted amine (*e.g.***6**) under acidic conditions. Synthesis of the required 1,4-dicarbonyl **7** would be achieved using a strategic enolate arylation of OBO-ketone **1**, and subsequent enolate alkylation using α-bromoacetophenone **8**.^11^ A possible one-pot procedure from **1** to **7**, by the sequential addition of **10** and **8** under basic conditions, was envisaged. Note that 25 g of **1** can be prepared from pyruvic acid in 2 steps in 44% yield.11*a*

**Scheme 3:**
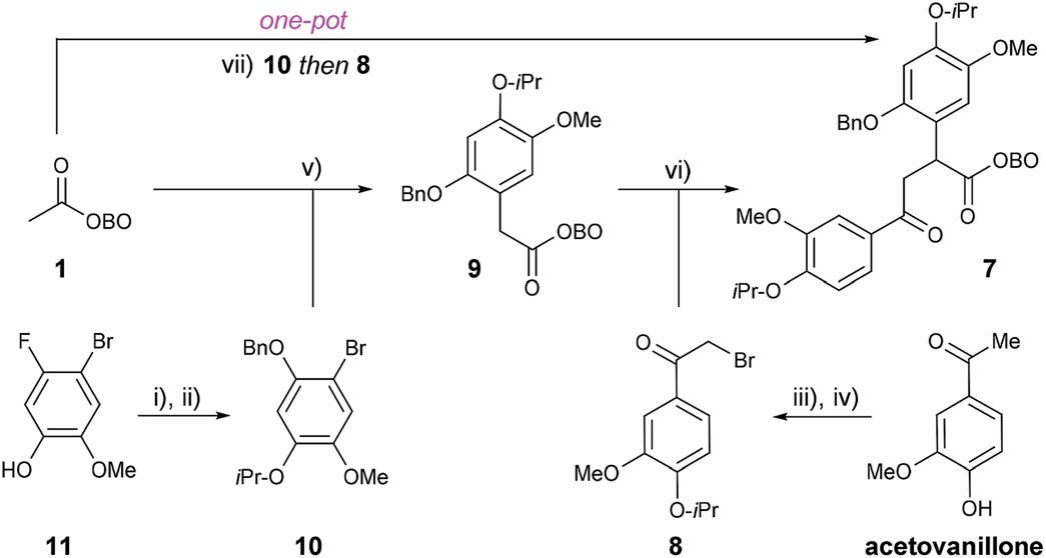
Synthesis of 1,4-dicarbonyl **7** from **1**. Reagents and conditions: (i) 2-bromopropane (3 eq.), K_2_CO_3_ (3 eq.), DMSO, 70 °C, 5 h, 97%; (ii) BnOH (4 eq.), NaH (4.6 eq.), NMP, 100 °C, 2 h, 86%; (iii) 2-bromopropane (1.5 eq.), K_2_CO_3_ (2 eq.), DMSO, 55 °C, 3.5 h, 96%; (iv) (±)-10-camphorsulfonic acid (1.9 eq.), NBS (1 eq.), MeCN, 85 °C, 1.5 h, 91%; (v) NaO^*t*^Bu (2.5 eq.), Pd(dtbpf)Cl_2_ (5 mol%), THF, 50 °C, 24 h, 75%; (vi) **8** (1.2 eq.), NaO^*t*^Bu (1.2 eq.), THF, 1.5 h, rt, 100%; (vii) **10** (1 eq.), NaO^*t*^Bu (2.5 eq.), Pd(dtbpf)Cl_2_ (5 mol%), THF, 50 °C, 52 h, *then***8** (2 eq.), 30 min, 78%.

We set out to synthesise α-aryl OBO-ketone **9** by Pd-catalysed enolate arylation of methyl-OBO-ketone **1** with aryl bromide **10**. The aryl bromide **10** was itself prepared from commercially available 4-bromo-5-fluoro-2-methoxyphenol (**11**) by O-alkylation with 2-bromopropane and subsequent nucleophilic aromatic substitution (S_N_Ar) at C-5 with sodium benzylate.^12^ The S_N_Ar route to allow installation of a protected phenol is much shorter and more efficient than a traditional Baeyer–Villiger reaction of an aromatic aldehyde (for example, compound **10** was synthesized in 5 steps from isovanillin, see ESI Section S1†). Through a short screening process, optimal conditions for the coupling of **1** and **10** were found, using NaO^*t*^Bu and Pd(dtbpf)Cl_2_ (5 mol%) in THF to give **9** in 75% yield (Scheme 3).^11^,^13^ The α-bromoacetophenone **8** required for alkylation of **9** was prepared by O-alkylation of acetovanillone with 2-bromopropane followed by α-bromination using (±)-10-camphorsulfonic acid/NBS. Treatment of **9** with NaO^*t*^Bu followed by addition of **8** gave the desired 1,4-dicarbonyl **7** in quantitative yield after purification. Having established a first-generation route to 1,4-dicarbonyl **7**, we were keen to explore a direct one-pot protocol from **1**. It was found that **7** could be prepared in one step from methyl-OBO-ketone **1** by sequential addition of **10** and **8**. Optimal conditions involved initial treatment of methyl-OBO-ketone **1** with NaO^*t*^Bu in the presence of aryl bromide **10** and Pd(dtbpf)Cl_2_ (5 mol%) before quenching with **8**. Protic workup then gave 1,4-dicarbonyl **7** in 78% yield after purification (Scheme 3).

**Scheme 4:**
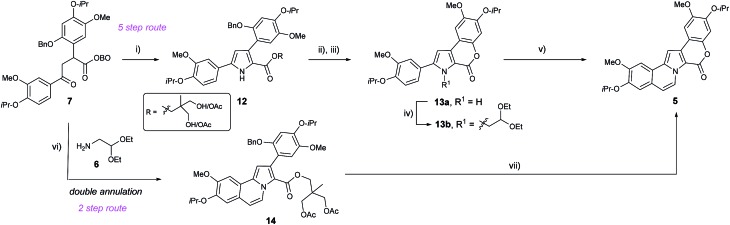
Synthesis of pyrrole **5**. Reagents and conditions: (i) NH_4_OAc (10 eq.) AcOH, 110 °C, 30 min; (ii) H_2_, Pd/C, EtOH, rt, 6.5 h; (iii) K_2_CO_3_ (2 eq.), EtOH, 90 °C, 2 h, 95% over 3 steps; (iv) BrCH_2_CH(OEt)_2_ (6.6 eq.), Cs_2_CO_3_ (6.5 eq.), DMF, 110 °C, 24 h, 86%; (v) TfOH (1 M in DCM), DCM, –10 to –5 °C, 24 h, 88%; (vi) **6** (9 eq.), AcOH, H_2_O (1 mol%), HCO_2_H (1 mmol%), 100 °C, 16 h, 89%; (vii) 20% Pd(OH)_2_/C, 1,4-cyclohexadiene (25 eq.), MeOH/EtOAc, 60 °C, 6 h then K_2_CO_3_, 89%.

After establishing a convenient route to 1,4-dicarbonyl **7** in one step from methyl-OBO-ketone **1**, we probed methods for condensation to form the pyrrole core. Treatment of **7** with NH_4_OAc in refluxing acetic acid resulted in successful formation of the pyrrole core, together with opening of the OBO cage to give esters **12** (Scheme 4). Without purification, **12** was immediately debenzylated using H_2_ and Pd/C to reveal a phenol which, upon treatment with K_2_CO_3_ in MeOH, underwent lactonisation to give **13a** in 95% yield over three steps. In order to install the alkene and fused ring motif, *N*-alkylation of the pyrrole using bromoacetaldehyde diethyl acetal was performed, using reported conditions giving **13b** as an unstable oil in 86% yield.^14^ Electrophilic aromatic substitution (S_E_Ar) by treatment of the acetal intermediate **13b** with catalytic TfOH,^14^ and subsequent *in situ* elimination then gave *N*-vinyl pyrrole **5** in 88% yield.

Despite this high yielding synthesis of key intermediate **5**, efforts were also directed towards an alternative and shorter retrosynthetic strategy, envisaging installation of two rings directly from 1,4-dicarbonyl **7** by using an acetal substituted amine.^15^ Therefore, treatment of **7** with aminoacetaldehyde diethyl acetal (**6**) in glacial acetic acid led to the formation of several pyrrole products from which the desired product **14** was observed by ^1^H NMR spectroscopy in small quantities. It was then found that heating **7** at reflux in ‘wet’ (1 mol% H_2_O with 1 mmol% formic acid) acetic acid delivered **14** in 89% yield. The presence of catalytic amounts of water and formic acid likely promoted the desired hydrolysis of the OBO-ester and any acetal intermediate(s). It is noteworthy that this reaction allows construction of both the pyrrole and fused carbocycle ring in a single step.

With successful isolation of **14**, the benzyl group was successfully removed by transfer hydrogenation using Pd(OH)_2_/C (Pearlman's catalyst) and 1,4-cyclohexadiene. The phenol intermediate was observed by TLC analysis, and quenching of the reaction mixture with K_2_CO_3_ resulted in rapid lactonisation to deliver **5** in 89% yield after purification.

**Scheme 5:**
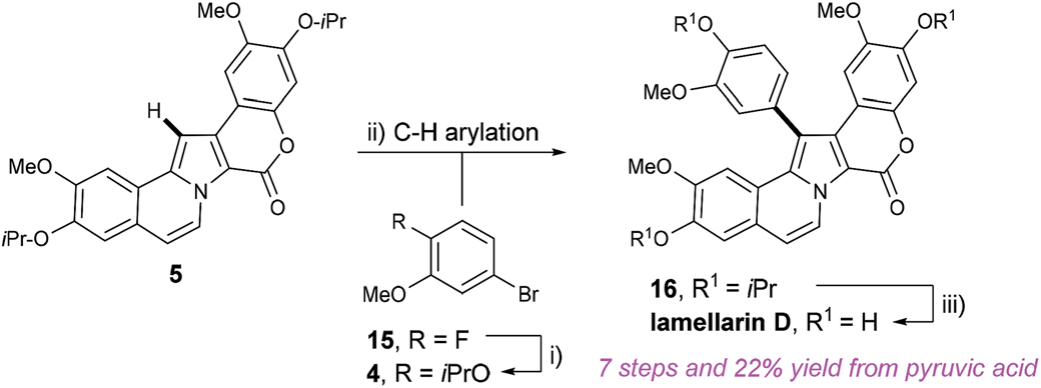
Synthesis of lamellarin D. Reagents and conditions: (i) ^i^PrOH (4.5 eq.), KO^*t*^Bu (4.0 eq.), PhMe, DMPU, 80 °C, 30 min, *then***15** (1 eq.), 3 h, 82%; (ii) Pd(PPh_3_)_2_Cl_2_ (5 mol%), KOAc (2.0 eq.), **4** (2.5 eq.), DMA, 150 °C, 22 h, 80%; (iii) BCl_3_ (1 M in heptane, 9 eq.), DCM, –78 °C to rt, 3.5 h, 99%.

Having devised a route to pyrrole **5** from methyl-OBO-ketone **1** in only three steps, efforts then focused on the envisaged and key C–H arylation of **5** with aryl halide fragment **4**. The synthesis of aryl bromide **4** was accomplished by utilising nucleophilic aromatic substitution of commercially available 5-bromo-2-fluoroanisole (**15**) at C-2 with KO^i^Pr (Scheme 5).12*b* Once more S_N_Ar chemistry on an unactivated substrate allowed a very short route to a protected phenol. Initial conditions for C–H arylation involved treatment of **5** with KOAc, Pd(PPh_3_) (5 mol%) and aryl bromide **4** in DMA at 150 °C for 16 h.^16^ Improved yields were realised by switching the extraction solvent from Et_2_O to CH_2_Cl_2_ and eventually **16** was isolated in 80% yield. It should be noted that this is the first late-stage pyrrole C–H arylation in a lamellarin alkaloid synthesis; traditional routes to lamellarins have almost universally used pyrrole halogenation followed by C–C cross coupling reactions. However, note that impressive early-stage Rh-catalysed C–H arylation in the synthesis of lamellarins I and C was demonstrated by Yamaguchi and co-workers in 2014.^17^ Finally, we could then complete the synthesis and access lamellarin D by using BCl_3_ to remove all isopropyl ethers.^18^

This synthesis of lamellarin D over 7 steps and 22% overall yield from pyruvic acid is short and efficient and compares very well to others in the literature. Notable steps include di-functionalisation of methyl-OBO-ketone **1** to the 1,4-dicarbonyl **7** in a one-pot procedure, convenient synthesis of coupling partners **10** and **4** using S_N_Ar reactivity, rapid construction of the pyrrole and carbocyclic core in a single step and C–H arylation of late-stage pyrrole intermediate **5**. This synthesis will provide convenient access to lamellarin D and analogues for biological and pharmaceutical research.

### Lamellarin Q

**Scheme 6:**
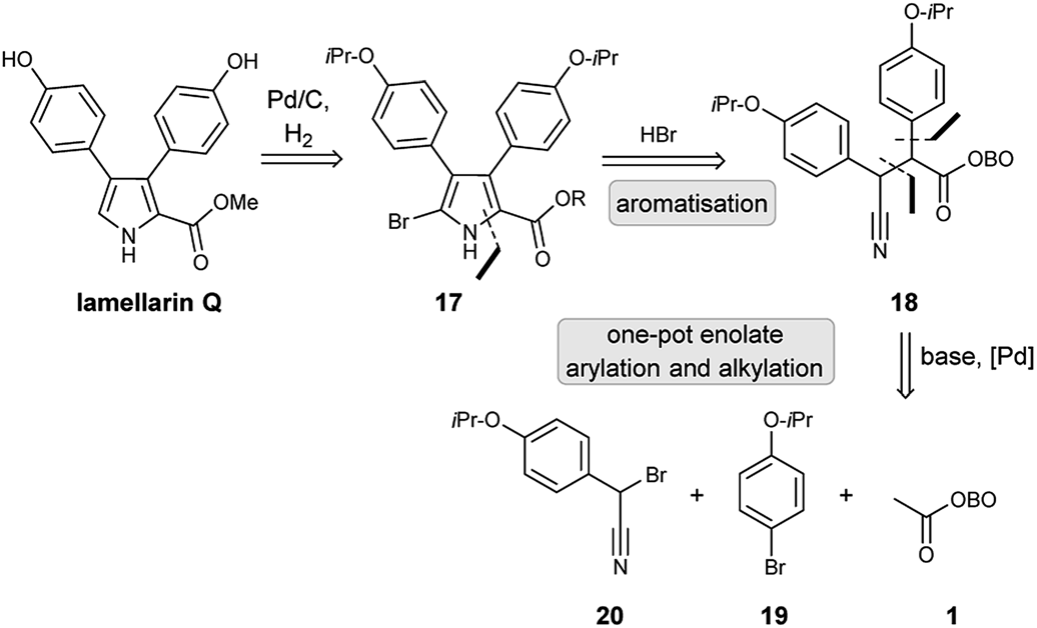
Retrosynthetic analysis of lamellarin Q.

Retrosynthetic analysis of lamellarin Q commenced with the synthesis of C-5 bromopyrrole **17**; this could be prepared by an unusual HBr-assisted cyclisation/aromatisation of β-cyano ketone **18**.^19^ Debromination could likely be achieved using standard partial hydrogenolysis conditions (H_2_, Pd/C), as has been reported in the literature.^20^ β-Cyano ketone **18** could be accessed by employment of the key masked pyruvate functionalisation strategy (Scheme 6). Treatment of methyl-OBO-ketone **1** with aryl bromide **19** under Pd catalysis and basic conditions should facilitate enolate arylation and subsequent enolate quenching with bromo-benzonitrile **20** was envisaged to give **18**.

**Scheme 7:**
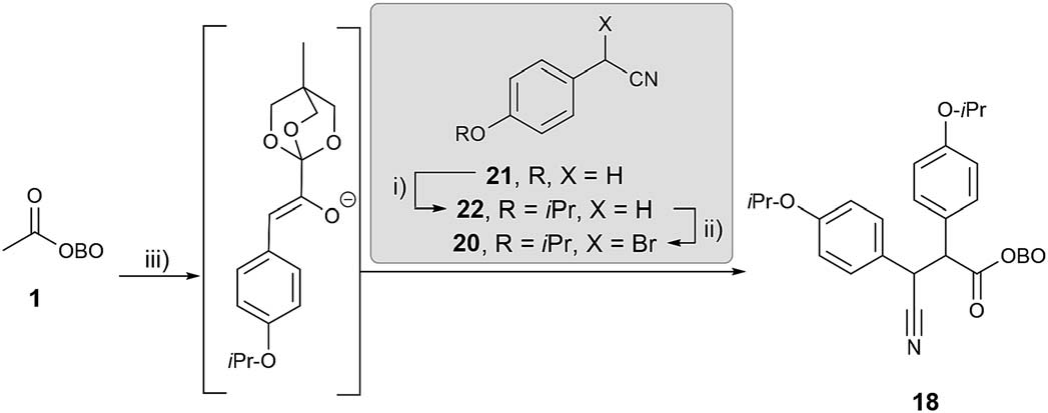
Synthesis of β-cyano ketone intermediate **18**. Reagents and conditions: (i) 2-bromopropane (1.5 eq.), K_2_CO_3_ (2.0 eq.), DMSO, >99%; (ii) NBS (1.5 eq.), dibenzoyl peroxide (4 mol%), (EtO)_2_CO, 100 °C, 24 h, 60%; (iii) **19** (1.6 eq.), Pd(dtbpf)Cl_2_ (5 mol%), NaO^*t*^Bu (2.5 eq.), THF, 55 °C, 20 h *then***20** (1.4 eq.), 3 h, 75%.

Initial work investigated the desired functionalisation of methyl-OBO-ketone **1** with aryl bromide **19** and α-bromo benzyl nitrile **20**. Protection of 4-hydroxybenzyl nitrile (**21**) with 2-bromopropane gave **22** and benzylic bromination using NBS/dibenzoyl peroxide in diethyl carbonate^21^ gave **20**. Using the previously developed conditions,^11^ NaO^*t*^Bu and Pd(dtpbf)Cl_2_ (5 mol%) in THF, methyl-OBO-ketone **1** coupled with aryl bromide **19**. After 24 h, the enolate was quenched with α-bromo benzyl nitrile **20** to deliver **18** in 75% yield in one step and as a single diastereomer (unassigned) after workup and purification (Scheme 7).

**Scheme 8:**
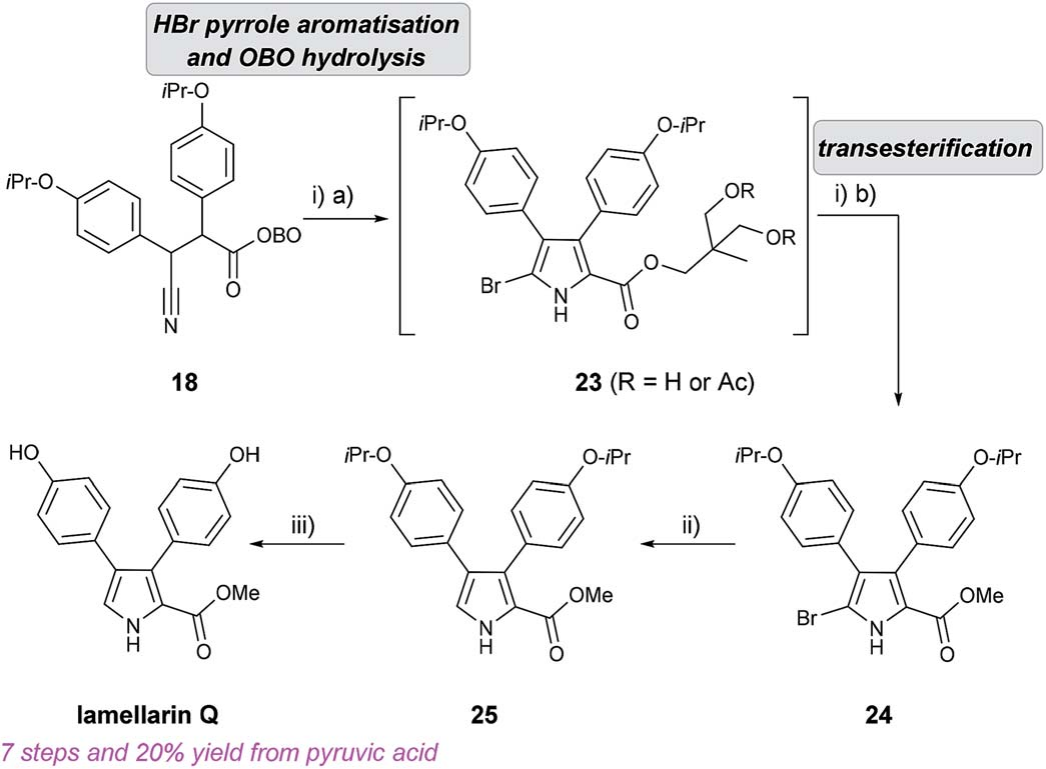
Completion of the synthesis of lamellarin Q. Reagents and conditions: (i) (a) 33% HBr in AcOH, DCM/Et_2_O (1 : 1), 0 °C to rt, 3.5 h; (b) K_2_CO_3_ (2 eq.), MeOH, 65 °C, 62% over 2 steps; (ii) H_2_, 10% Pd/C, NaOAc (2.1 eq.), MeOH, rt, 1 h, >99%; (iii) BBr_3_ (3 eq.), DCM, –78 °C to rt, >99%.

With β-cyano ketone intermediate **18** in hand, the key HBr-assisted pyrrole aromatisation was examined.^19^ Treatment of **18** with 33% HBr in AcOH in DCM/Et_2_O as solvent system led to consumption of starting material (by TLC analysis) in under 20 min, to give a complex mixture of unidentified intermediates which was simplified after stirring at rt for 3 h. After neutralisation using K_2_CO_3_ and work up, the obtained crude mixture (which consisted of a mixture of OBO ring-opened bromo-pyrroles **23**) was exposed to MeOH/K_2_CO_3_ to give the methyl bromopyrrole carboxylate **24** in 62% yield, after purification. Partial hydrogenolysis with H_2_ and Pd/C cleanly removed the bromine atom to give pyrrole **25** and final removal of both isopropyl ethers using BBr_3_ gave lamellarin Q as an unstable solid (Scheme 8).

## Conclusions

To conclude, we have demonstrated the synthetic utility of OBO-ester masked α-keto acid **1** as a valuable building block for convenient access to important type I and type II lamellarin natural products. The bioinspired strategy was accomplished *via* the one-pot Pd-mediated arylation and alkylation reaction of ketone **1** to give either 1,4-dicarbonyl (**7**) or β-cyano ketone (**18**) intermediates. Efficient single step double annulation of **7**, followed by lactone formation and Pd-catalysed direct arylation completed the type I skeleton of lamellarin D, while HBr promoted aromatisation of **18** provided rapid access to the type II skeleton of lamellarin Q. These syntheses can be regarded as short and efficient: lamallarin D and Q were both prepared in seven steps from pyruvic acid with 22% and 20% yields, respectively.

## Conflicts of interest

There are no conflicts to declare.

## Supplementary Material

Supplementary information
