# Supplementary material for: Synthesis of lamellarin alkaloids using orthoester-masked α-keto acids
Source: Chem Sci. 2019 Mar 19;10(15):4334–8. doi: 10.1039/c8sc05678a (PMC6471603; doi:10.1039/c8sc05678a)
Supplement: Supplementary file 1 [file SC-010-C8SC05678A-s001.pdf]

Electronic Supplementary Material (ESI) for Chemical Science.
This journal is © The Royal Society of Chemistry 2019

Electronic Supplementary Information

Synthesis of Lamellarin Alkaloids Using Orthoester-Masked α-Keto Acids

Harry J. Shirley,[†] *Maria Koyioni,*[†] *Filip Muncan and Timothy J. Donohoe**

Department of Chemistry, University of Oxford, Chemistry Research Laboratory, Mansfield Road, Oxford, OX1 3TA (UK)

*E-mail: timothy.donohoe@chem.ox.ac.uk

Table of Contents **Page**

S1. Alternative Synthesis of 1-(Benzyloxy)-2-bromo-5-isopropoxy-4-methoxybenzene (**10**)	3
S2. General Methods and Materials	3
S3. Experimental Procedures and Spectroscopic Data	4
S4. ^{13}C NMR Data Lamellarin D and Q Compared to Previously Reported Data	19
S5. References	20
S6. NMR Spectra	21

S1. Alternative Synthesis of 1-(Benzyloxy)-2-bromo-5-isopropoxy-4-methoxybenzene (10)

An alternative synthesis of 1-(benzyloxy)-2-bromo-5-isopropoxy-4-methoxybenzene (**10**) is feasible from **isovanillin**. Initial phenol protection with 2-bromopropane to give **S1**[1] was followed by Baeyer-Villiger oxidation with *m*-CBPA and a $B_{Ac}2$ cleavage with K_2CO_3 to give phenol **S2** in 83% yield.[1] Protection of the phenol moiety with benzyl bromide gave **S3** in 99% yield,[2] and subsequent bromination with NBS gave the desired aryl bromide **10** (97% yield).[1]

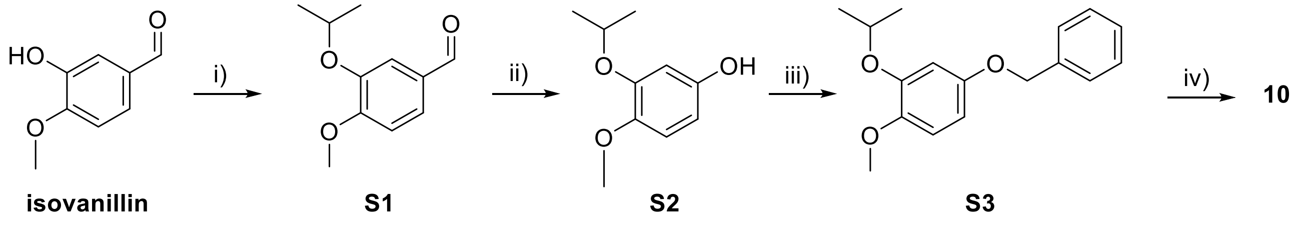

i) K_2CO_3 (2 eq.), *i*PrBr (1.5 eq.), DMSO, 55 °C, 2.5 h, 96%; ii) *m*-CPBA (1.1 eq.), CH_2Cl_2, 0 °C, 4 h, then K_2CO_3 (2.4 eq.), MeOH, rt, 13 h, 83%; iii) K_2CO_3 (2 eq.), BnBr (1.5 eq.), DMF, 0 °C to rt, 13 h, 99%; iv) NBS (1.1 eq.), DMF, 0 °C to rt, 1 h, 97%.

Scheme S1. Alternative synthesis of 1-(benzyloxy)-2-bromo-5-isopropoxy-4-methoxybenzene (**10**)

S2. General Methods and Materials

Solvents were dried over 4 Å molecular sieves or activated alumina columns. All commercially available reagents were used without further purification unless otherwise specified. [1,1′-Bis(diphenylphosphino)ferrocene]dichloropalladium(II) was purchased from Alfa Aesar. NaO*t*Bu solution (2 M in THF) was purchased from Sigma-Aldrich (grade ZerO2®). All anhydrous reactions were carried out in flame-dried glassware and under an inert atmosphere of argon unless otherwise specified. Brine refers to a saturated aqueous solution of NaCl. ^{1}H, ^{13}C and ^{19}F NMR experiments were conducted on a Bruker AVII400 or AVIII400 instrument at 400, 101 and 377 MHz respectively. Chemical shifts, δ, are reported relative to residual solvent peaks and quoted in parts per million (ppm) to the nearest 0.01 ppm with splitting patterns recorded as singlet (s), doublet (d), triplet (t), quartet (q), quintet (quint), heptet (hept), doublet of doublets (dd), multiplet (m) and broad singlet (br s). Scalar coupling constants, *J*, are measured in Hz accurate to one decimal place. Infrared spectra were recorded as thin films or neat samples on a Bruker Tensor 27 FT-IR spectrometer equipped with Attenuated Total Reflectance sampling accessories. High-resolution mass spectra are given to five decimal places and were recorded on a Bruker MicroTof (resolution = 10000 FWHM) under conditions of electrospray ionization (ESI), electronic ionization (EI), chemical ionization (CI) and atmospheric-pressure chemical ionization (APCI). Melting points were obtained from recrystallized samples using a Leica VMTG heated-stage microscope. The solvent(s) used for recrystallization are quoted in

parentheses after the melting point. Flash column chromatography was performed using silica gel (60 Å, 0.033-0.070 mm, BDH). TLC analysis was performed on Merck Kiesegel 60 F_{254} 0.25 mm pre-coated silica plates. 1-(4-Methyl-2,6,7-trioxabicyclo[2.2.2]octan-1-yl)ethan-1-one (**1**),[3] and 1-bromo-4-isopropoxybenzene (**19**)[4] were prepared according to literature procedures.

Systematic names were generated by the computer program ChemDraw. However, the numbering on the structures does not correspond to this systematic name. The NMR assignments follow the numbering system shown on the structures for straightforward comparison of data

S3. Experimental Procedures and Spectroscopic Data

3-Isopropoxy-4-methoxybenzaldehyde (S1)

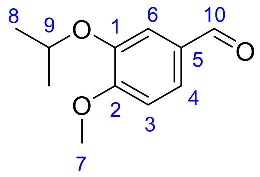

Isovanillin (15.0 g, 98.5 mmol, 1.0 eq.) was dissolved in DMSO (295 mL) in a flame-dried round-bottom flask flushed with argon. Anhydrous K_2CO_3 (27.3 g, 197 mmol, 2.0 eq.) was added to the stirred solution and subsequently, 2-bromopropane (13.8 mL, 148 mmol, 1.5 eq.) was added in one portion at rt, before the reaction mixture was heated at 55 °C for 2.5 h. The reaction mixture was cooled to rt and diluted with water. The mixture was extracted with Et_2O (3 × 100 mL) and the combined organic extracts were washed successively with 10% aq. NaOH (150 mL), water (150 mL) and brine (100 mL), before being dried over Na_2SO_4. The solvent was evaporated *in vacuo* and the residue was recrystallized to give the *title compound* **S1** (18.4 g, 96%) as a pale-yellow solid. **m.p.** 32–35 °C (*n*-pentane/Et_2O); **IR** ν_{max} (thin film): 2977, 1687, 1584, 1508, 1435, 1396, 1338, 1264, 1239, 1160, 1132, 1023, 914, 810 cm^{-1}; **^{1}H NMR (400 MHz, $CDCl_3$)** δ = 9.83 (1H, s, C(10)*H*O), 7.44 (1H, d, *J* = 8.1 Hz, *H*C(4)$_{Ar}$), 7.41 (1H, s, *H*C(6)$_{Ar}$), 6.97 (1H, d, *J* = 8.1 Hz, *H*C(3)$_{Ar}$), 4.64 (1H, hept, *J* = 6.1 Hz, OC(9)*H*), 3.94 (3H, s, OC(7)H_3), 1.40 (6H, d, *J* = 6.1 Hz, C(8)H_3); **^{13}C NMR (101 MHz, $CDCl_3$)** δ = 191.1, 155.7, 148.0, 130.2, 126.7, 112.7, 111.0, 71.4, 56.3, 22.0. Spectroscopic data are consisted with those previously reported in the literature.[5]

3-Isopropoxy-4-methoxyphenol (S2)

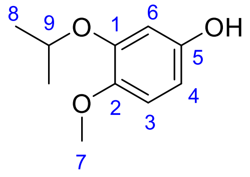

Benzaldehyde **S1** (10.0 g, 51.5 mmol, 1.0 eq.) was dissolved in DCM (100 mL), cooled to 0 °C under argon atmosphere and *m*-chloroperbenzoic acid (25% w/w, 13.2 g, 57.1 mmol, 1.1 eq.) was added in 6 portions every 10 minutes to the stirred solution. After being stirred for 3 h, an additional *m*-chloroperbenzoic acid (0.6 g, 5 mol%, 2.6 mmol) was added. The reaction mixture was left to stir for an additional 1 h after which time it was quenched by the addition of saturated aq. $NaHCO_3$ (30 mL). The mixture was

diluted with water and the aqueous layer was extracted with CH_2Cl_2 (3 × 100 mL). The combined organic extracts were washed with brine, dried over Na_2SO_4, and evaporated *in vacuo*. The residue was dissolved in methanol (200 mL) and K_2CO_3 (17.3 g, 125 mmol, 2.4 eq.) was added in 4 portions over 20 mins to the solution at rt. After being stirred for 13 h, the solvent was evaporated *in vacuo* before water (100 mL) was added to the residue and the product was extracted with Et_2O (3 × 100 mL). The combined organic extracts were washed with brine, dried over Na_2SO_4, filtered and evaporated *in vacuo*. Recrystallization gave the *title compound* **S2** (7.8 g, 83%) as a colorless solid. **m.p.** 120–124 °C (*n*-pentane/Et_2O) [lit.[1] 121–122 °C (*n*-pentane/Et_2O)]; **IR** ν_{max} (thin film): 3418 (O-H), 2980, 2836, 1605, 1509, 1462, 1374, 1285, 1222, 1163, 1122, 1029, 995, 921, 834 cm^{-1}; **^{1}H NMR (400 MHz, $CDCl_3$)** δ = 6.73 (1H, d, *J* = 8.5 Hz, *H*C(3)$_{Ar}$), 6.48 (1H, d, *J* = 2.1 Hz, *H*C(6)$_{Ar}$), 6.34 (1H, dd, *J* = 8.5, 2.1 Hz, *H*C(4)$_{Ar}$), 4.56 (1H, s, O*H*), 4.49 (1H, hept, *J* = 6.1 Hz, OC(9)*H*), 3.79 (3H, s, OC(7)H_3), 1.37 (6H, d, *J* = 6.1 Hz, C(8)H_3); **^{13}C NMR (101 MHz, $CDCl_3$)** δ = 150.3, 148.1, 144.2, 113.5, 106.6, 104.1, 71.4, 56.8, 22.0. Physical and spectroscopic data are consistent with those previous reported in the literature.[1]

4-(Benzyloxy)-2-isopropoxy-1-methoxybenzene (S3)

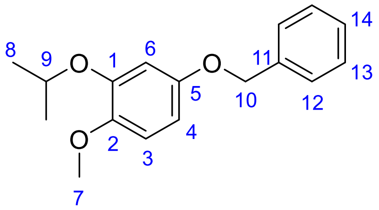

Phenol **S2** (2.0 g, 11 mmol, 1.0 eq.) was dissolved in DMF (22 mL) and cooled to 0 °C before K_2CO_3 (2.3 g, 16.5 mmol, 1.5 eq.) and benzyl bromide (2.2 mL, 18.7 mmol, 1.7 eq.) were subsequently added to the stirred solution. The stirred solution was left to warm to rt over 13 h, after which time it was quenched with saturated aq. NH_4Cl (20 mL) and the product was extracted with Et_2O (3 × 50 mL). The combined organic extracts were washed with brine, dried over Na_2SO_4 and filtered. The solvent was evaporated *in vacuo* and the residue was recrystallized to give the *title compound* **S3** (2.94 g, 99%) as colorless needles. **m.p.** 56–57 °C (*n*-pentane/Et_2O); **IR** ν_{max} (thin film): 2981, 2889, 1608, 1508, 1457, 1382, 1258, 1227, 1164, 1137, 1080, 1044, 1028, 955 cm^{-1}; **^{1}H NMR (400 MHz, $CDCl_3$)** δ = 7.28 - 7.45 (5H, m, *H*C(12, 13, 14)$_{Ar}$), 6.79 (1H, d, *J* = 9.1 Hz, *H*C(3)$_{Ar}$), 6.60 (1H, s, *H*C(6)$_{Ar}$), 6.49 (1H, d, *J* = 9.1 Hz, *H*C(4)$_{Ar}$), 5.00 (2H, s, OC(10)H_2Ph), 4.49 (1H, hept, *J* = 6.1 Hz, OC(9)*H*), 3.81 (3H, s, OC(7)H_3), 1.35 (6H, d, *J* = 6.1 Hz, C(8)H_3); **^{13}C NMR (101 MHz, $CDCl_3$)** δ = 153.4, 148.2, 145.0, 137.2, 128.6, 128.0, 127.6, 112.8, 105.0, 104.7, 71.3, 70.7, 56.7, 22.1; **HRMS** (CI^+) Found $[M+H]^+$ = 273.1480; $C_{17}H_{21}O_3$ requires 273.1485, Δ–1.91 ppm.

1-Bromo-2-fluoro-4-isopropoxy-5-methoxybenzene (S4)

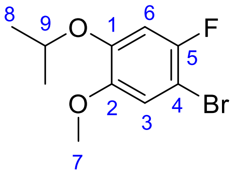

To a solution of 4-bromo-5-fluoro-2-methoxyphenol (**11**) (1.0 g, 4.5 mmol, 1.0 eq.) in DMSO (10 mL) was added K_2CO_3 (1.9 g, 14 mmol, 3.0 eq.) and 2-bromopropane (1.3 mL, 14 mmol, 3.0 eq.) before a reflux condenser was attached and the stirred mixture was heated at 70 °C for 5 h. After this time, water (100 mL) and DCM (50 mL) were added after cooling to rt, before the organic layer was separated and dried with $MgSO_4$. The residue was dry loaded onto SiO_2 and purified by flash column chromatography (SiO_2; *n*-pentane/acetone, 95:5 to 90:10) to give the *title compound* **S4** (1.15 g, 97%) as pale-yellow needles. **m.p.** 52–57 °C (*n*-pentane/acetone); **IR** ν_{max} (thin film): 3115, 2978, 1601, 1500, 1334, 1176, 1109, 1008 cm^{-1}; **^{1}H NMR (400 MHz, $CDCl_3$)** δ = 6.96 (1H, d, $^4J_{H\text{-}F}$ = 6.7 Hz, C(3)*H*), 6.70 (1H, d, $^3J_{H\text{-}F}$ = 10.1 Hz, C(6)*H*), 4.51-4.42 (1H, m, C(9)*H*), 3.81 (3H, s, C(7)H_3), 1.36 (6H, d, *J* = 6.0 Hz, C(8)H_3); **^{13}C NMR (101 MHz, $CDCl_3$)** δ = 153.5 (d, J_{CF} = 238 Hz), 147.6 (d, J_{CF} = 8.6 Hz), 147.2 (d, J_{CF} = 2.3 Hz), 115.8, 104.2 (d, J_{CF} = 25.9 Hz), 97.8 (d, J_{CF} = 23.3 Hz), 72.1, 56.7, 21.9; **^{19}F NMR (377 MHz, $CDCl_3$)** δ −115.9; **HRMS** expected ions not observed.

1-(Benzyloxy)-2-bromo-5-isopropoxy-4-methoxybenzene (10)

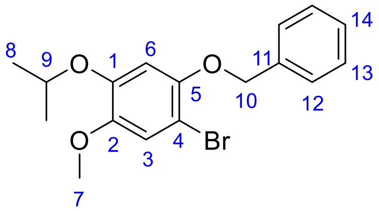

Method A: A solution of **S3** (500 mg, 1.84 mmol, 1.0 eq.) in DMF (2 mL) was cooled to 0 °C before a solution of NBS (340 mg, 1.92 mmol, 1.1 eq.) in DMF (2.5 mL) was added over 30 min *via* a syringe. After being stirred for 1 h, the reaction mixture was diluted with water at 0 °C and then allowed to warm to rt. The product was extracted with Et_2O (3 × 30 mL) and the combined organic extracts were washed with brine, dried over Na_2SO_4 and filtered. The solvent was evaporated *in vacuo*, and recrystallization from *c*-hexane gave the *title compound* **10** (620 mg, 97%) as pale orange needles. **m.p.** 72–73 °C (*c*-hexane); **IR** ν_{max} (thin film): 3034, 2976, 2932, 1581, 1501, 1456, 1441, 1394, 1379, 1311, 1268, 1213, 1177, 1110, 1030, 925, 842 cm^{-1}; **H NMR (400 MHz, $CDCl_3$)** δ = 7.28–7.50 (5H, m, *H*C(12, 13, 14)$_{Ar}$), 7.04 (1H, s, *H*C(3)$_{Ar}$), 6.57 (1H, s, *H*C(6)$_{Ar}$), 5.08 (2H, s, OC(10)H_2Ph), 4.39 (1H, hept, *J* = 6.1 Hz, OC(9)*H*), 3.80 (3H, s, OC(7)H_3), 1.28 (6H, d, *J* = 6.1 Hz, C(8)H_3); **^{13}C NMR (101 MHz, $CDCl_3$)** δ = 149.1, 147.0, 145.9, 136.8, 128.6, 128.0, 127.4, 117.0, 106.0, 103.2, 72.3, 72.1, 56.7, 22.0; **HRMS** (APCI$^+$) Found $[M+H]^+$ = 351.0583; $C_{17}H_{20}BrO_3$ requires 351.0590, Δ–2.1 ppm.

Method B: To a solution of benzyl alcohol (1.3 mL, 12.5 mmol, 4.0 eq.) in anhydrous NMP (13 mL), NaH (568 mg, 60% in mineral oil, 14.2 mmol, 4.6 eq.) was cautiously added portion wise at rt. After 20 mins the evolution of H_2 had subsided and the mixture was transferred using a syringe, equipped

with a short 10-gauge needle, to an RBF containing a stirred solution of **S4** (808 mg, 3.1 mmol, 1.0 eq.) in NMP (16 mL). The reaction was heated to 100 °C, gradually gaining a dark blue color, with continued stirring for 2 h. After this time the reaction was poured onto a mixture of crushed ice (200 g) and PhMe (200 mL) and allowed to warm to rt. The organic layer was separated, dry loaded onto SiO_2 and flash column chromatography (SiO_2; *n*-pentane/*t*-BuOMe, 95:5 to 90:10) gave the *title compound* **10** (940 mg, 86%) as pale-yellow needles. Data are consistent with those obtained *via* method A.

1-(4-Isopropoxy-3-methoxyphenyl)ethan-1-one (S5)

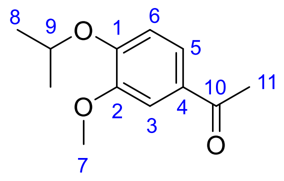

Acetovanillone (500 mg, 3.0 mmol, 1.0 eq.) was dissolved in DMSO (8.7 mL). Anhydrous K_2CO_3 (830 mg, 6.0 mmol, 2.0 eq.) was added to the stirred solution, and subsequently 2-bromopropane (420 µL, 4.5 mmol, 1.5 eq.) was added in one portion at rt, before the reaction mixture was heated at 55 °C for 3.5 h. The reaction mixture was cooled to rt and diluted with water. The biphasic mixture was extracted with Et_2O (3 × 50 mL) and the combined organic extracts were washed successively with 10% aq. NaOH (30 mL), water (30 mL), and brine (30 mL), before being dried over Na_2SO_4. The solvent was evaporated *in vacuo* and recrystallization of the residue gave the *title compound* **S5** (600 mg, 96%) as a pale yellow waxy solid. **m.p.** 31–32 °C (*n*-pentane/Et_2O); **IR** ν_{max} (thin film): 2978, 2935, 1672 (C=O), 1585, 1507, 1464, 1416, 1385, 1374, 1356, 1264, 1218, 1175, 1149, 1133, 1108, 1078, 1032, 951, 876, 847, 810cm^{-1}; **^{1}H NMR (400 MHz, $CDCl_3$)** δ = 7.55 (1H, d, *J* = 8.3 Hz, *H*C(5)$_{Ar}$), 7.53 (1H, s, *H*C(3)$_{Ar}$), 6.88 (1H, d, *J* = 8.3 Hz, *H*C(6)$_{Ar}$), 4.66 (1H, hept, *J* = 6.1 Hz, OC(9)*H*), 3.91 (3H, s, OC(7)H_3), 2.56 (3H, s, C(11)H_3), 1.41 (6H, d, *J* = 6.1 Hz, C(8)H_3); **^{13}C NMR (101 MHz, $CDCl_3$)** δ = 197.0, 152.0, 150.0, 130.3, 123.3, 112.8, 110.8, 71.3 , 56.2 , 26.4, 22.0; **HRMS** (ESI$^+$) Found $[M+H]^+$ = 209.11729; $C_{12}H_{17}O_3$ requires 209.11722, Δ+0.33 ppm.

2-Bromo-1-(4-isopropoxy-3-methoxyphenyl)ethan-1-one (8)

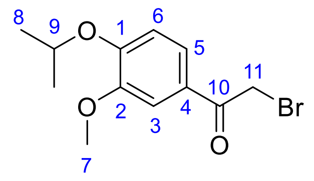

Acetophenone **S5** (139 mg, 0.67 mmol, 1.0 eq.) and (±)-10-camphorsulfonic acid (300 mg, 1.29 mmol, 1.9 eq.) were added to a flame-dried RBF containing acetonitrile (5 mL) over flame-dried 3Å molecular sieves (*ca*. 500 mg). To the strongly stirred mixture was added NBS (119 mg, 0.67 mmol, 1.0 eq.) before refluxing at 85 °C for 1.5 h. After this time the mixture was cooled to rt before SiO_2 (*ca.* 3.0 g) was added and the solvent was removed *in vacuo*. The solid mixture was loaded onto a column for purification (SiO_2, *n*-pentane/acetone, 80:20) to give the *title compound* **8** (175 mg, 91%) as pale-yellow crystals. **m.p.** 70–71 °C (*c*-hexane); **IR** ν_{max} (thin film): 2976, 1666, 1583, 1514, 1469,

1421, 1388, 1290, 1239, 1153, 1111, 1024, 956, 860 cm^{-1}; **^{1}H NMR (400 MHz, $CDCl_3$)** δ = 7.57 (1H, d, *J* = 8.3 Hz, *H*C(5)$_{Ar}$), 7.53 (1H, s, *H*C(3)$_{Ar}$), 6.89 (1H, d, *J* = 8.3 Hz, *H*C(6)$_{Ar}$), 4.67 (1H, hept, *J* = 6.1 Hz, OC(9)*H*), 4.40 (2H, s, C(11)H_2Br), 3.90 (3H, s, OC(7)H_3), 1.41 (6H, d, *J* = 6.1 Hz, C(8)H_3); **^{13}C NMR (101 MHz, $CDCl_3$)** δ = 190.1, 152.8, 150.1, 126.7, 123.9 , 112.6, 111.5, 71.4, 56.2, 30.6, 22.0; **HRMS** (ESI$^+$) Found [M+H]$^+$ = 287.02780; $C_{12}H_{16}O_3Br$ requires 287.02773, Δ+0.24 ppm.

2-[2-(Benzyloxy)-4-isopropoxy-5-methoxyphenyl]-1-(4-methyl-2,6,7-trioxabicyclo[2.2.2]octan-1-yl)ethan-1-one (9)

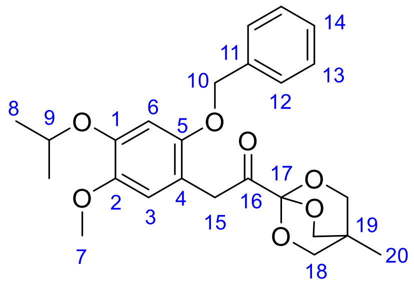

In a flame-dried Biotage® microwave vial flushed with argon, was added **10** (225 mg, 0.64 mmol, 1.1 eq.) methyl-OBO-ketone **1** (150 mg, 0.87 mmol, 1.5 eq.), Pd(dtbpf)Cl_2 (19 mg, 29 µmol, 5 mol%) and THF (5.1 mL). After this NaO*t*Bu (2M in THF, 720 µL, 1.45 mmol, 2.5 eq.) was added to the stirred solution and the vial was flushed with argon for 5 mins after which time the vial was sealed with an aluminium microwave vial cap (Reseal™ septum). The solution was heated at 50 °C for 24 h. The resulting mixture was filtered through a short plug of SiO_2 using EtOAc as eluent, concentrated *in vacuo*, dry-loaded onto SiO_2 and purified by column chromatography (*n*-pentane/EtOAc, 60:40) to give the *title compound* **9** (212 mg, 75%) as pale-yellow needles. **m.p.** 122–124 °C (*n*-pentane/Et_2O); **IR** ν_{max} (thin film): 3657, 2981, 2886, 1753 (C=O), 1509, 1462, 1383, 1252, 1220, 1154, 1075, 1051, 1004, 955 cm^{-1}; **^{1}H NMR (400 MHz, $CDCl_3$)** δ = 7.27–7.43 (5H, m, *H*C(12, 13, 14)$_{Ar}$), 6.64 (1H, s, *H*C(3)$_{Ar}$), 6.52 (1H, s, *H*C(6)$_{Ar}$), 4.97 (2H, s, OC(10)H_2Ph), 4.38 (1H, hept, *J* = 5.9 Hz, OC(9)*H*), 4.01 (6H, s, OC(18)H_2), 3.99 (2H, s, C(15)H_2), 3.77 (3H, s, OC(7)H_3), 1.29 (6H, d, *J* = 6.1 Hz, C(8)H_3), 0.87 (3H, s, C(20)H_3); **^{13}C NMR (101 MHz, $CDCl_3$)** δ = 195.9, 150.9, 146.9, 144.6, 137.5, 128.4, 127.6, 127.3, 115.9, 115.0, 103.9, 103.8, 73.1, 71.9, 71.4, 56.7, 37.9, 30.9, 22.2, 14.3; **HRMS** (APCI$^+$) Found [M+H]$^+$ = 443.20633; $C_{25}H_{31}O_7$ requires 443.20643, Δ–0.23 ppm.

2-[2-(Benzyloxy)-4-isopropoxy-5-methoxyphenyl]-4-(4-isopropoxy-3-methoxyphenyl)-1-(4-methyl-2,6,7-trioxabicyclo[2.2.2]octan-1-yl)butane-1,4-dione (7)

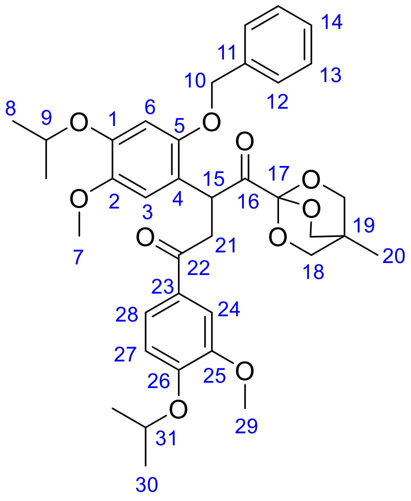

Method A: In a flame-dried RBF flushed with argon, **9** (44.3 mg, 0.10 mmol, 1.0 eq.) was dissolved in THF (1.0 mL) before NaO*t*Bu (2M in THF, 60 μL, 0.12 mmol, 1.2 eq.) was added and the resulted solution was left stirring for 20 mins. A solution of **8** (34.5 mg, 0.12 mmol, 1.2 eq.) in THF (1.0 mL) was added dropwise to the stirred solution of **9** *via* a syringe (addition rate: 6 mL/h). After complete addition, the solution was left to stir for an additional 1.5 h, after which time it was quenched with saturated aq. NH_4Cl (5 mL) and extracted with DCM (3 × 30 mL). The combined organic extracts were dried with Na_2SO_4, filtered, and the solvent was evaporated *in vacuo*. The residue was dry-loaded onto SiO_2 and purified by column chromatography (*n*-pentane/acetone, 80:20) to give the *title compound* **7** (64.9 mg, 100%) as colorless needles.

Method B (one-pot): To a flame-dried Biotage® microwave vial flushed with argon was added **10** (112.4 mg, 0.32 mmol, 1.1 eq.), methyl-OBO-ketone **1** (75 mg, 0.44 mmol, 1.5 eq.), Pd(dtbpf)Cl_2 (9.5 mg, 14.5 μmol, 5 mol%) and THF (2.5 mL). Subsequently, NaO*t*Bu (2 M in THF, 360 μL, 0.73 mmol, 2.5 eq.) was added *via* a syringe and the vial was flushed with argon for 5 mins after which time the vial was sealed with an aluminium microwave vial cap (Reseal™ septum). The solution was heated at 50 °C for 52 h, after which time a solution of **8** (184 mg, 0.64 mmol, 2.0 eq.) in anhydrous THF (5 mL) was added dropwise *via* a syringe (addition rate: 5 mL/h). After complete addition, the solution was left to stir for an additional 30 min at 50 °C, after which time it was left to cool to rt, quenched with saturated aq. NH_4Cl (10 mL) and extracted with DCM (3 × 30 mL). The combined organic extracts were dried with $MgSO_4$, filtered, and the solvent was evaporated *in vacuo*. The residue was dry-loaded onto SiO_2 and purified by column chromatography (*n*-pentane/acetone, 75:25) to give the *title compound* **7** (161 mg, 78%) as colorless needles. **m.p.** 151–154 °C (*n*-pentane/Et_2O); **IR** ν_{max} (thin film): 2976, 2935, 2880, 1741, 1674, 1593, 1508, 1464, 1417, 1263, 1213, 1106, 1069, 1035, 1002, 950 cm^{-1}; **^{1}H NMR (400 MHz, $CDCl_3$)** δ = 7.52 (1H, d, *J* = 8.3 Hz, *H*C(28)$_{Ar}$), 7.50 (1H, s, *H*C(24)$_{Ar}$), 7.27 – 7.45 (5H, m, *H*C(12, 13, 14)$_{Ar}$), 6.80 (1H, d, *J* = 8.3 Hz, *H*C(27)$_{Ar}$), 6.65 (1H, s, *H*C(3)$_{Ar}$), 6.53 (1H, s, *H*C(6)$_{Ar}$), 5.37 (1H, dd, *J* = 9.9, 4.2 Hz, C(15)*H*), 5.05 (2H, s, OC(10)H_2), 4.62 (1H, hept, *J* = 6.1 Hz, OC(31)*H*), 4.41 (1H, hept, *J* = 6.1 Hz, OC(9)*H*), 3.91 (6H, s, OC(18)H_2), 3.84 (3H, s, OC(29)H_3), 3.80 (1H, dd, *J* = 17.8, 9.9 Hz, C(21)H_2), 3.75 (3H, s, OC(7)H_3), 3.17 (1H, dd, *J* = 17.8, 4.2 Hz, C(21)H_2), 1.39 (6H,

d, *J* = 6.1 Hz, C(30)*H*$_3$), 1.30 (6H, dd, *J* = 6.1, 3.8 Hz, C(8)*H*$_3$), 0.78 (3H, s, C(20)*H*$_3$); **^{13}C NMR (101 MHz, CDCl$_3$)** δ = 198.2, 196.1, 151.7, 150.0, 149.7, 146.6, 144.9, 137.5, 129.5, 128.5, 127.7, 127.2, 122.7, 118.9, 113.1, 112.7, 111.0, 104.4, 104.1, 73.0, 71.8, 71.7, 71.1, 56.5, 56.0, 41.9, 40.8, 30.8, 22.2, 22.1, 22.0, 21.9 , 14.3; **HRMS** (ESI$^+$) Found [M+H]$^+$ = 649.30023; $C_{37}H_{45}O_{10}$ requires 649.30072, Δ–0.76 ppm.

7-Isopropoxy-2-(4-isopropoxy-3-methoxyphenyl)-8-methoxychromeno[3,4-*b*]pyrrol-4(3*H*)-one (13a)

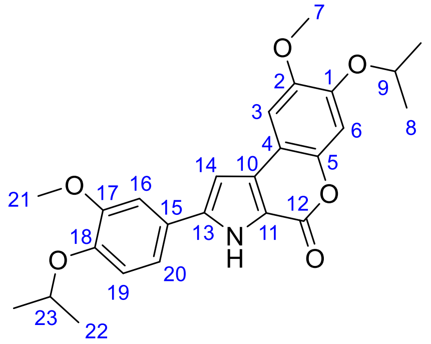

In a flame-dried RBF flushed with argon were added the 1,4-dicarbonyl **7** (65 mg, 0.10 mmol, 1.0 eq.) and NH_4OAc (77.1 mg, 1.0 mmol, 10.0 eq.) before the addition of AcOH (3 mL). A reflux condenser was attached, and the solution was heated at 110 °C for 30 min. The reaction mixture was left to cool to rt and then poured onto crushed ice. The mixture was neutralised by the careful addition of solid K_2CO_3 (as judged by litmus paper). The aqueous layer was extracted with DCM (3 × 30 mL) and the combined organic extracts were dried with $MgSO_4$, filtered and the solvent was evaporated *in vacuo*. The residue was transferred to an RBF and dissolved in EtOH (5 mL). To the stirred solution was added Pd/C (10% wt. loading, 24.0 mg) and the flask was flushed H_2 and left to stir under an atmosphere of H_2 (balloon) at rt for 6.5 h. After complete consumption of the starting material (as observed by TLC), the solution was filtered through filter paper using EtOH as eluent, and the solvent was evaporated *in vacuo*. The residue was dissolved in EtOH (4 mL) and powdered K_2CO_3 (28.0 mg, 0.20 mmol, 2 eq.) was added to the stirred solution and heated at 90 °C for 2 h. The solution was left to cool to rt and the solvent was evaporated *in vacuo*. The product was extracted with DCM (3 × 30 mL) and the extract was washed with brine (30 mL), dried over $MgSO_4$ and evaporated *in vacuo* to give the *title compound* **13a** (42 mg, 95%) as colorless needles. **m.p.** 263–264 °C (EtOH); **IR** ν_{max} (thin film): 3264, 2975, 2934, 2872, 2840, 1697, 1623, 1607, 1595, 1519, 1494, 1472, 1447, 1415, 1384, 1276, 1258, 1231, 1220, 1206, 1169, 1148, 1110, 1067, 1027, 1001, 941, 868, 842 cm^{-1}; **^{1}H NMR (400 MHz, CDCl$_3$)** δ = 11.05 (1H, s, N*H)*, 7.58 (1H, d, *J* = 2.1 Hz, *H*C(16)$_{Ar}$), 7.37 (1H, dd, *J* = 8.4, 2.1 Hz, *H*C(20)$_{Ar}$), 7.22 (1H, s, *H*C(3)$_{Ar}$), 6.99 (1H, d, *J* = 8.4 Hz, *H*C(19)$_{Ar}$), 6.92 (1H, s, *H*C(6)$_{Ar}$), 6.87 (1H, d, *J* = 2.3 Hz, C(14)*H*), 4.62 (2H, hept, *J* = 6.1 Hz, OC(9, 23)*H*), 4.13 (3H, s, OC(21)*H*$_3$), 3.98 (3H, s, OC(7)*H*$_3$), 1.43 (12H, d, *J* = 6.1 Hz, C(8, 22)*H*$_3$); **^{13}C NMR (101 MHz, CDCl$_3$)** δ = 156.2, 150.8, 148.2, 148.0, 147.6, 146.2, 143.5, 132.1, 123.9, 118.8, 116.4, 115.4, 110.4, 109.6, 105.1, 103.9, 98.9, 71.8, 71.5, 56.6, 56.5, 22.2, 22.0; **HRMS** (APCI$^+$) Found [M+H]$^+$ = 438.19061; $C_{25}H_{28}O_6N$ requires 438.19111, Δ–1.14 ppm.

3-(2,2-Diethoxyethyl)-7-isopropoxy-2-(4-isopropoxy-3-methoxyphenyl)-8-methoxychromeno[3,4-*b*]pyrrol-4(3*H*)-one (13b)

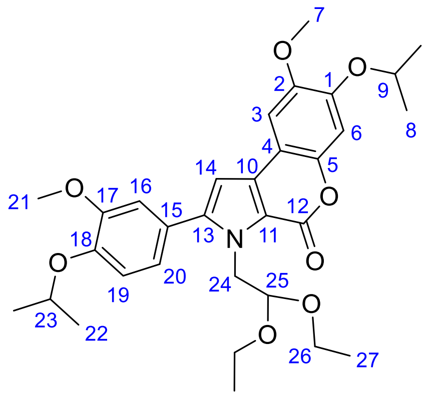

In a flame-dried Biotage® microwave vial flushed with argon, **13a** (100 mg, 0.23 mmol, 1.0 eq.) was dissolved in DMF (5 mL). To the stirred solution, Cs_2CO_3 (483 mg, 1.48 mmol, 6.5 eq.) and 2-bromo-1,1-diethoxyethane (225 μL, 1.50 mmol, 6.6 eq.) were added and the vial was then flushed with argon for 5 min after which time the vial was sealed with a microwave vial cap (Reseal™ septum). The solution was heated at 110 °C for 24 h. The resulting mixture was allowed to cool to rt and the product was extracted with DCM (3 × 30 mL) and the extract was washed with brine (30 mL), dried over $MgSO_4$ and evaporated *in vacuo*. The residue was dry-loaded onto SiO_2 and purified by column chromatography (SiO_2; *n*-pentane/acetone, 50:10) to yield the *title compound* **13b** (108 mg, 86%) as an unstable yellow oil. Installation of the acetal was confirmed by **^{1}H NMR (400 MHz, $CDCl_3$)** Diagnostic resonances δ = 5.05 (1H, t, *J* = 5.6 Hz, *H*C(25)), 4.49 (2H, d, *J* = 5.6 Hz, H_2C(24)), 3.67 (2H, q, *J* = 7.0 Hz, OC(26)H_2), 3.42 (2H, q, *J* = 7.0 Hz, OC(26)H_2) and 1.10 (6H, t, *J* = 7.0 Hz, H_3C(27)).

2-[({2-[2-(Benzyloxy)-4-isopropoxy-5-methoxyphenyl]-8-isopropoxy-9-methoxypyrrolo[2,1-*a*]-isoquinoline-3-carbonyl}oxy)methyl]-2-methylpropane-1,3-diyl diacetate (14)

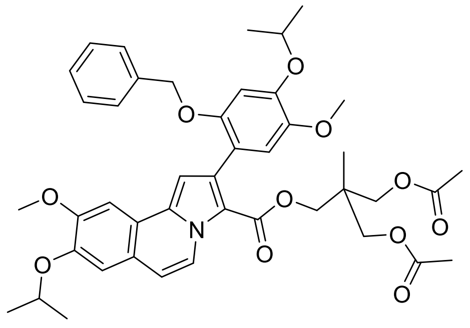

To an RBF containing **7** (100 mg, 0.16 mmol, 1.0 eq.) and aminoacetaldehyde diethyl acetal (**6**) (200 mg, 1.50 mmol, 9 eq.) was added a solution of AcOH (10 mL, with added 1 mol% water and *ca.* 0.001 mol% formic acid) *via* syringe, before a reflux condenser was attached and the stirred solution was heated at 100 °C over 16 h. After this time the solution was allowed to cool to rt and poured onto crushed ice, before adjustment to pH 7-8 by the portion wise addition of K_2CO_3 (as judged by litmus paper). The aqueous layer was extracted with EtOAc (20 mL), the organic layer was collected, dried over $MgSO_4$ and filtered. The filtrate was directly dry-loaded onto SiO_2 and purified by flash column chromatography (SiO_2, *n*-pentane/acetone, 80:20) to give the *title compound* **14** as yellow oil (108 mg, 89%). **IR** ν_{max} (thin film) 2978, 2930, 1700, 1616, 1509, 1486, 1466, 1418, 1385, 1324, 1223, 1179, 1137, 1038, 1018, 945 cm^{-1}; **^{1}H NMR (400 MHz, $CDCl_3$)** δ = 9.29 (1H, d, *J* = 7.5 Hz), 7.42 (1H, s), 7.22-7.14 (5H, m), 7.09 (1H, s), 6.94 (1H, d, *J* = 7.5 Hz), 6.88 (1H, s), 6.79 (1H, s), 6.68 (1H, s), 4.93 (2H, s) 4.71 (1H, hept, *J* = 6.1 Hz, O*i*Pr) 4.50 (1H, hept, *J* = 6.1 Hz, O*i*Pr), 4.05 (2H, s, CH_2), 3.98 (3H, s, OMe), 3.82 (3H, s, OMe), 3.68 (4H, s, 2 x CH_2), 1.97 (6H, s, 2 x OAc), 1.46 (6H, d, *J* = 6.1 Hz, O*i*Pr), 1.36 (6H, d, *J* = 6.1 Hz, O*i*Pr), 0.66 (3H, s, Me); **^{13}C NMR (101 MHz, $CDCl_3$)** δ = 170.8, 162.1, 151.1, 150.1, 148.5, 147.2, 145.0, 137.7, 134.5, 132.9, 128.4, 127.6,

127.2, 123.9, 123.1, 120.1, 119.2, 115.6, 113.2, 112.1, 110.7, 105.4, 104.4, 103.4, 72.0, 71.5, 66.0, 65.7, 56.8, 56.2, 37.9, 22.3, 22.1, 20.8, 16.8. **HRMS** (ESI$^+$) Found $[M+H]^+$ = 756.33699; $C_{43}H_{50}O_{11}N$ requires 756.33784, Δ–1.14 ppm.

3,11-Diisopropoxy-2,12-dimethoxy-6*H*-chromeno[4',3':4,5]pyrrolo[2,1-*a*]isoquinolin-6-one (5)

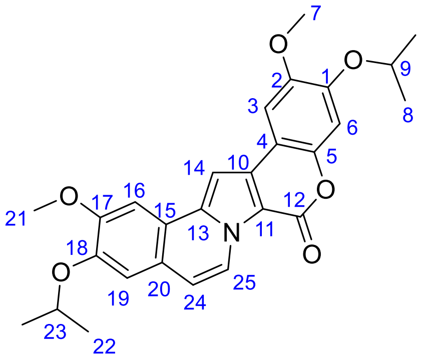

Method A: In a flame-dried RBF flushed with argon, **13b** (63 mg, 0.11 mmol, 1.0 eq.) was dissolved in DCM (5 mL). In a flame-dried pear-shaped flask flushed with argon, an emulsion of TfOH in DCM (1.0 M, 1 mL) was made and stirred vigorously at rt. Two drops of the TfOH emulsion were added *via* a syringe to the stirred solution containing **13b** at –10 °C. After 6 h, a further two drops of TfOH emulsion was added and the solution was allowed to warm to –5 °C over 18 h. After complete consumption of the starting material as observed by TLC (25 h), the reaction was quenched by addition of $NaHCO_3$ (139 mg, 1.65 mmol, 15 eq.) and EtOH (0.6 mL) before warming to rt and then the solvent was evaporated *in vacuo*. The residue was dry-loaded onto SiO_2 and purified by column chromatography (*n*-pentane/acetone, 80:20) to give the *title compound* **5** (46.1 mg, 88%) as colorless needles.

Method B: To a RBF containing **14** (100 mg, 0.13 mmol, 1.0 eq.) and 1,4-cyclohexadiene (0.3 mL, 3.2 mmol, 25 eq.) in MeOH (5 mL) and EtOAc (0.5 mL) was added 20% $Pd(OH)_2$ on carbon (Pearlman's catalyst) (88 mg). After flushing with N_2, the mixture was heated to 60 °C over 6 h. After this time K_2CO_3 (1.0 g) was added before cooling to rt and mixture was filtered over a 5 cm pad of diatomaceous earth, eluting with DCM (50 mL) into an RBF. The filtrate was directly dry-loaded onto SiO_2 and purified by flash column chromatography (SiO_2, *n*-pentane/acetone, 80:20) to give the *title compound* **5** (54 mg, 89%) as an off-white solid. **IR** ν_{max} (thin film): 3469, 3375, 2978, 2950, 2349, 2154, 1695 (C=O), 1666, 1619, 1594, 1499, 1438, 1420, 1365, 1336, 1317, 1297, 1235, 1190, 1144, 1100, 1031, 1009, 991, 964, 924, 855cm^{-1}; **^{1}H NMR (400 MHz, CDCl$_3$)** δ = 9.07 (1H, d, *J* = 7.3 Hz, *H*C(25)*)*, 7.54 (1H, s, *H*C(16)$_{Ar}$), 7.32 (1H, s, *H*C(3)$_{Ar}$), 7.13 (1H, s, *H*C(14)$_{Ar}$), 7.12 (1H, s, *H*C(19)$_{Ar}$), 6.99 (1H, d, *J* = 7.3 Hz, *H*C(24)$_{Ar}$) overlaps with 6.99 (1H, s, *H*C(6)$_{Ar}$), 4.74 (1H, hept, *J* = 6.1 Hz, OC(23)*H*), 4.61 (1H, hept, *J* = 6.1 Hz, OC(9)*H*), 4.07 (3H, s, OC(21)H_3), 4.00 (3H, s, OC(7)H_3), 1.48 (6H, d, *J* = 6.1 Hz, C(22)H_3), 1.45 (6H, d, *J* = 6.1 Hz, C(8)H_3); **^{13}C NMR (101 MHz, CDCl$_3$)** δ = δ 155.6, 151.3, 149.4, 148.7, 147.4, 146.8, 138.6, 132.4, 124.3, 123.5, 118.5, 112.3, 110.7, 109.8, 109.3, 105.4, 104.8, 103.8, 91.4, 77.5, 77.2, 76.8, 71.7, 71.5, 56.7, 56.3, 27.1, 22.1, 22.0. **HRMS** (ESI$^+$) Found $[M+H]^+$ = 462.19095;

$C_{27}H_{28}O_6N$ requires 462.19111, Δ–0.36 ppm. Spectroscopic data are consistent with those previously reported.[6]

4-Bromo-1-isopropoxy-2-methoxybenzene (4)

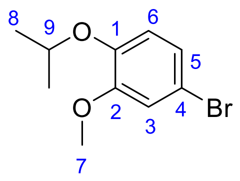

To a suspension of KO*t*Bu (898 mg, 8.0 mmol, 4.0 eq.) in PhMe (2 mL) was added 2-propanol (690 µL, 9.0 mmol, 4.5 eq.) in anhydrous DMPU (2 mL) before the mixture was heated at 80 °C for 30 mins. After this time, 5-bromo-2-fluoroanisole **15** (410 mg, 2.0 mmol, 1.0 eq.) in anhydrous DMPU (1 mL) was added (syringe washed with further 0.2 mL DMPU), before a reflux condenser was immediately attached and the solution was stirred at 80 °C for a further 3 h. After this time, the mixture was cooled to rt before brine (20 mL) and *n*-pentane (20 mL) were sequentially added. After vigorous shaking of the biphasic mixture in a separating funnel, the organic layer was separated and filtered through a 5 cm plug of SiO_2 and washed with *n*-pentane (20 mL). The solvent was evaporated to give the *title compound* **4** (401 mg, 82%) as colorless oil. **IR** ν_{max} (thin film): 2975, 2933, 2830, 1585, 1493, 1464, 1444, 1397, 1383, 1372, 1322, 1290, 1248, 1221, 1178, 1133, 1107, 1028, 951, 868, 835, 813 cm^{-1}; **^{1}H NMR (400 MHz, $CDCl_3$)** δ = 6.97 (1H, dd, *J* = 9.1, 2.3 Hz, *H*C(5)$_{Ar}$) overlaps with 6.96 (1H, d, *J* = 2.3 Hz, *H*C(3)$_{Ar}$), 6.74 (1H, d, *J* = 9.1 Hz, *H*C(6)$_{Ar}$), 4.45 (1H, hept, *J* = 6.1 Hz, OC(9)*H*), 3.81 (3H, s, OC(7)H_3), 1.32 (6H, d, *J* = 6.1 Hz, C(8)H_3); **^{13}C NMR (101 MHz, $CDCl_3$)** δ = 151.3, 146.5, 123.4, 117.2, 115.4, 113.2, 71.8, 56.1, 22.0. Spectroscopic data are consistent with those previously reported.[1]

3,11-Diisopropoxy-14-(4-isopropoxy-3-methoxyphenyl)-2,12-dimethoxy-6*H*-chromeno[4',3':4,5]-pyrrolo[2,1-*a*]isoquinolin-6-one (16)

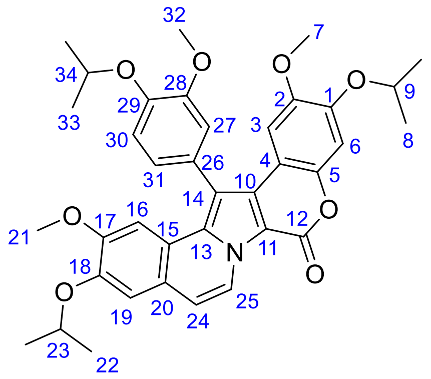

In a flame-dried Biotage® microwave vial containing DMA (0.3 mL) were added **5** (23.8 mg, 51.6 µmol, 1.0 eq.), $Pd(PPh_3)_2Cl_2$ (2 mg, 2.8 µmol, 5 mol%), anhydrous KOAc (10 mg, 102 µmol, 2.0 eq.), and **4** (23 µL, 129 µmol, 2.5 eq.) under argon. The vial was then flushed with argon for 5 min after which time the vial sealed with an aluminium microwave vial cap (Reseal™ septum) and the solution was heated at 150 °C for 22 h. The resulting mixture was allowed to cool to rt and diluted with water (10 mL). The aqueous layer was extracted with DCM (3 × 20 mL) and the combined organic extracts were washed with brine (30 mL), dried over $MgSO_4$, and evaporated *in vacuo*. The residue was dry-loaded onto SiO_2 and purified by column chromatography (*n*-pentane/acetone, 85:15) to yield the *title compound* **16** (25.7 mg, 80%) as off-white needles. **m.p.** 192–194 °C [lit.[6] 191–192 °C (DCM/Et_2O)]; **IR** ν_{max} (thin film): 2976, 2934, 1704 (C=O), 1614, 1534,

1509, 1486, 1465, 1431, 1418, 1385, 1334, 1266, 1223, 1179, 1162, 1137, 1110, 1038, 1018, 945, 857 cm^{-1}; **^{1}H NMR (400 MHz, $CDCl_3$)** δ = 9.20 (1H, d, *J* = 7.3 Hz, *H*C(25)), 7.17 (1H, s, *H*C(16)$_{Ar}$), 7.16 (1H, s, *H*C(31)$_{Ar}$) overlaps with 7.16 (1H, s, *H*C(30)$_{Ar}$), 7.13 (1H, s, *H*C(27)$_{Ar}$), 7.09 (1H, s, *H*C(19)$_{Ar}$), 7.01 (1H, d, *J* = 7.3 Hz, *H*C(24)$_{Ar}$), 6.95 (1H, s, *H*C(6)$_{Ar}$), 6.75 (1H, s, *H*C(3)$_{Ar}$), 4.69 (1H, hept, *J* = 6.1 Hz, OC(23)*H*), 4.63 (1H, hept, *J* = 6.1 Hz, OC(34)*H*), 4.56 (1H, hept, *J* = 6.1z, OC(9)*H*), 3.84 (3H, s, OC(32)H_3), 3.44 (3H, s, OC(7)H_3), 3.43 (3H, s, OC(21)H_3), 1.43 (12H, d, *J* = 6.1 Hz, C(22, 33)H_3), 1.39 (6H, d, *J* = 6.1 Hz, C(8)H_3); **^{13}C NMR (101 MHz, $CDCl_3$)** δ = 155.6, 151.4, 150.2, 148.5, 147.9, 147.2, 146.6, 146.5, 134.4, 129.4, 128.8, 124.7, 123.9, 123.2, 119.0, 116.9, 115.0, 112.3, 111.0, 110.4, 110.0, 107.8, 105.7, 105.5, 103.4, 71.8, 71.5, 71.2, 56.2, 55.5, 55.2, 21.8–21.9 (6 × CHCH_3); **HRMS** (ESI$^+$) Found $[M+H]^+$ = 626.27460; $C_{37}H_{40}O_8N$ requires 626.27484, Δ–0.39 ppm. Spectroscopic data are consistent with those previously reported.[6]

3,11-Dihydroxy-14-(4-hydroxy-3-methoxyphenyl)-2,12-dimethoxy-6*H*-chromeno[4',3':4,5]pyrrolo-[2,1-*a*]isoquinolin-6-one, Lamellarin D

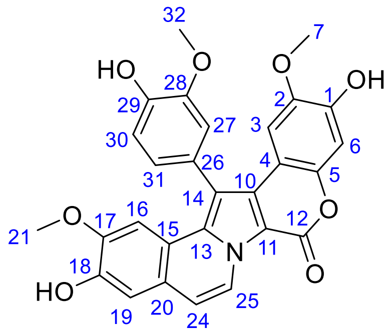

In a flame-dried RBF flushed with argon, **16** (25.7 mg, 41.1 μmol, 1.0 eq.) was dissolved in DCM (6 mL) and then cooled to –78 °C. BCl_3 (1.0 M in *n*-heptane, 370 μL, 9.0 eq.) was then added *via* a syringe to the stirred solution. After 30 minutes, the solution was allowed to warm to rt and left to stir for a further 3 h, after which time it was quenched by the addition of saturated aq. $NaHCO_3$ (2 mL). The product was extracted with EtOAc (3 × 20 mL) and the combined organic extracts were dried over $MgSO_4$ and evaporated *in vacuo*. The residue was dry-loaded onto SiO_2 and purified by column chromatography (SiO_2; EtOAc) to yield **lamellarin D** (20.4 mg, 99%) as a pale green solid. **^{1}H NMR (700 MHz, DMSO-*d*$_6$)** δ = 9.94 (1H, s), 9.84 (1H, s), 9.34 (1H, s), 8.99 (1H, d, *J* = 7.4 Hz), 7.20 (1H, d, *J* = 7.4 Hz), 7.18 (1H, s), 7.15 (1H, d, *J* = 2.0 Hz), 7.13 (1H, s), 7.09 (1H, d, *J* = 7.9 Hz), 7.00 (1H, dd, *J* = 2.0, 7.9 Hz), 6.86 11(1H, s), 6.71 (1H, s), 3.77 (3H, s), 3.38 (3H, s) and 3.38 (3H, s) overlap with H_2O resonance; **^{13}C NMR (101 MHz, DMSO-*d*$_6$)** δ = 154.4, 148.7, 148.5, 148.4, 147.9, 146.9, 146.4, 144.6, 134.1, 129.0, 125.5, 124.7, 123.9, 122.1, 117.6, 116.5, 115.1, 112.4, 111.6, 110.9, 108.4, 106.4, 105.8, 105.4, 103.8, 56.0, 55.1, 54.6; **HRMS** (ESI$^+$) Found $[M+Na]^+$ = 522.11591; $C_{28}H_{21}O_8N^{23}Na$ requires 522.11594, Δ–0.06 ppm. Spectroscopic data are consistent with those previously reported.[5]

2-(4-Isopropoxyphenyl)acetonitrile (22)

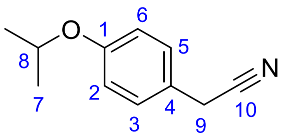

2-Bromopropane (14 mL, 0.15 mol, 1.5 eq.) was added to a mixture of 4-hydroxyphenylacetonitrile (13.3 g, 0.1 mol, 1.0 eq.) and K_2CO_3 (28 g, 0.2 mol, 2.0 eq.) in DMSO (50 mL). The reaction mixture was stirred at 60 °C for 21 h and then left to cool to rt. Water (100 mL) was added and the aqueous layer extracted with DCM (4 × 80 mL). The combined organic extracts were washed with brine, dried with Na_2SO_4, filtered and concentrated *in vacuo* to give the *title compound* **22** (17 g, >99%) as pale-yellow needles. **m.p.** 26-28 °C (*n*-pentane/Et_2O); **IR** ν_{max} 3042, 2976 and 2906, 2248, 1611, 1581, 1508, 1469, 1452, 1416, 1380, 1373, 1352, 1333, 1304, 1286, 1246, 1205, 1180, 1137, 1117, 1103, 1013, 953, 925, 902, 863, 832, 806 cm^{-1}; **^{1}H NMR (400 MHz, $CDCl_3$)** δ = 7.21 (2H, d, *J* = 8.7 Hz, HC_{Ar}), 6.88 (2H, d, *J* = 8.7 Hz, HC_{Ar}), 4.54 (1H, hept, *J* = 6.1 Hz, OC(8)*H*), 3.67 (2H, s, C(9)H_2CN), 1.33 (6H, d, *J* = 6.0 Hz, C(7)H_3); **^{13}C NMR (101 MHz, $CDCl_3$)** δ = 157.6, 129.1, 121.5, 118.2, 116.4, 70.0, 22.8, 21.9; **HRMS**: Found $[M+Na]^+$ = 198.08925, $C_{11}H_{13}ON^{23}Na$ requires 198.08894, Δ = +1.58 ppm.

2-Bromo-2-(4-isopropoxyphenyl)acetonitrile (20)

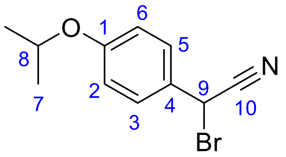

To a two-neck RBF was added dibenzoyl peroxide (97 mg, 0.04 mmol, 4 mol%), 2-(4-isopropoxyphenyl)acetonitrile **22** (1.75 g, 10 mmol, 1.0 eq.) and NBS (2.67 g, 15 mmol, 1.5 eq.). Diethyl carbonate (which had been degassed by freeze-pump-thaw cycles) (50 mL) was added under argon before heating to 100 °C, over 24 h. The reaction mixture was left to cool to rt and concentrated *in vacuo*. The residue was triturated with pentane/Et_2O, filtered and washed with Et_2O. The filtrate was concentrated *in vacuo* and flash column chromatography (SiO_2; *n*-pentane/acetone, 96:4) gave the *title compound* **20** (1.53 g, 60%) as orange oil. **IR** ν_{max} 2978 and 2935, 2247, 1606, 1582, 1509, 1466, 1454, 1428, 1385, 1375, 1331, 1305, 1250, 1180, 1120, 1106, 950, 863, 834 cm^{-1}; **^{1}H NMR (400 MHz, $CDCl_3$)** δ = 7.46 (2H, d, *J* = 8.8 Hz, HC_{Ar}), 6.90 (2H, d, *J* = 8.8 Hz, HC_{Ar}), 5.49 (1H, s, C(9)*H*), 4.58 (1H, hept, *J* = 6.0 Hz, C(8)*H*), 1.35 (6H, d, *J* = 6.0 Hz, C(7)H_3); **^{13}C NMR (101 MHz, $CDCl_3$)** δ = 159.5, 129.4, 124.9, 116.5, 116.3, 70.2, 27.8, 21.9; **HRMS**: Expected ions were not observed.

2,3-Bis(4-isopropoxyphenyl)-4-(4-methyl-2,6,7-trioxabicyclo[2.2.2]octan-1-yl)-4-oxobutanenitrile (18)

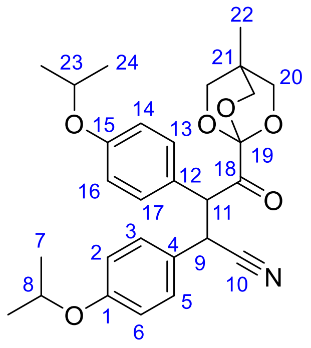

To a stirring solution of **1** (100 mg, 0.58 mmol, 1.0 eq.), **19** (201 mg, 0.93 mmol, 1.6 eq.) and Pd(dtbpf)Cl_2 (38 mg, 0.058 mmol, 0.1 eq.) in THF (5 mL) was added NaO*t*Bu (2M in THF, 0.72 mL, 2.5 eq.) at rt. The solution was then heated at 55 °C over 20 h. After this time, a solution of freshly prepared **20** (223 mg, 0.79 mmol, 1.4 eq.) in THF (1 mL) was added dropwise and the mixture was left stirring for additional 3 h. After this time Et_2O (7 mL), saturated aq. NH_4Cl (1 mL) and water (1.5 mL) were added. The aqueous layer was extracted with Et_2O (2 x 10 mL), dried over $MgSO_4$ and filtered. The filtrate was dry loaded onto SiO_2 and purification by flash column chromatography (SiO_2; *n*-pentane/EtOAc, 80:20) gave the *title compound* **18** as a single diastereoisomer (208 mg, 75%), as pale-yellow crystals. **m.p.** 151-153 °C (*n*-pentane/Et_2O); **IR** ν_{max} 3070, 3038, 2977, 2929, 2887, 2236, 1751, 1610, 1581, 1508, 1469, 1399, 1384, 1372, 1350, 1301, 1243, 1178, 1119, 1106, 1083, 1065, 103, 1003, 954, 879, 858, 843 cm^{-1}; **^{1}H NMR (400 MHz, $CDCl_3$)** δ = 7.20 (2H, d, *J* = 8.8 Hz, HC_{Ar}), 7.19 (2H, d, *J* = 8.8 Hz, HC_{Ar}), 6.84 (2H, d, *J* = 8.7 Hz, HC_{Ar}), 6.81 (2H, d, *J* = 8.7 Hz, HC_{Ar}), 4.62 (1H, d, *J* = 10.0 Hz, C*H*), 4.58–4.49 (2H, m, C(8)*H* & C(23)*H*), 4.46 (1H, d, *J* = 10.0 Hz, C*H*), 3.80 (6H, s, C(20)H_2), 1.38–1.24 (12H, m, OCH(C(24)H_3)$_2$ & OCH(C(7)H_3)$_2$), 0.74 (3H, s, C(22)H_3); **^{13}C NMR (101 MHz, $CDCl_3$)** δ = 194.4, 158.0, 157.7, 130.2, 129.6, 125.8, 125.4, 119.7, 116.1, 115.9, 103.6, 72.9, 69.9, 69.8, 56.0, 40.6 , 30.8, 22.07, 22.06, 22.0, 14.1; **HRMS**: Found $[M+H]^+$ = 480.23810, $C_{28}H_{34}NO_6$, requires 480.23806 Δ+0.07 ppm.

Methyl 5-bromo-3,4-bis(4-isopropoxyphenyl)-1*H*-pyrrole-2-carboxylate (24)

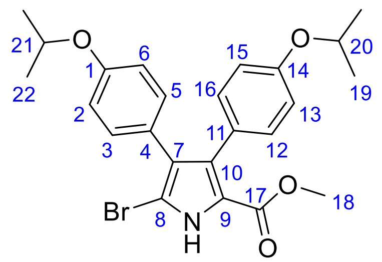

A solution of **18** (143.9 mg, 0.3 mmol) in DCM/Et_2O (1:1, 2 mL) was added dropwise to a solution of 33% HBr in AcOH (920 µL) at 0 °C and the mixture left stirring at this temperature for 30 min. After this time, the mixture left to warm to rt. After 3 h the reaction mixture was poured onto crushed ice and adjusted to *ca.* pH 7-8 (as judged by pH indicator paper) by slow addition of K_2CO_3. The solution was then extracted with DCM (4 × 50 mL), and the combined organic extracts dried with Na_2SO_4, filtered and concentrated *in vacuo*. The remaining residue was dissolved in MeOH (9 mL) and K_2CO_3 (82.9 mg, 0.6 mmol, 2 eq.) was added. The mixture heated at 65 °C for 4 h. Then the mixture left to cool to rt, dry loaded onto SiO_2 and purified by flash column chromatography (SiO_2; *n*-pentane/Et_2O, 80:20) to give the *title compound* **24** (87.3 mg, 62%) as colorless plates. **m.p.** 153-154 °C (*c*-hexane); **IR** ν_{max} 3247

(N-H), 2975 and 2933 (alkyl C-H), 1678, 1610, 1573, 1549, 1530, 1507, 1461, 1441, 1417, 1385, 1335, 1305, 1283, 1238, 1181, 1171, 1136, 1118, 1020, 1004, 953, 859, 843 cm^{-1}; **^{1}H NMR (400 MHz, $CDCl_3$)** δ = 9.13 (1H, s, N*H*), 7.07 (2H, d, *J* = 8.7 Hz, HC_{Ar}), 7.01 (2H, d, *J* = 8.8 Hz, HC_{Ar}), 6.75 (4H, d, *J* = 8.7 Hz, HC_{Ar}), 4.57–4.45 (2H, hept, *J* = 6.0 Hz, $OCH(CH_3)_2$), 3.74 (3H, s, $C(18)H_3$), 1.32 (6H, d, *J* = 6.1 Hz, $OCH(CH_3)_2$), 1.32 (6H, d, *J* = 6.1 Hz, $OCH(CH_3)_2$); **^{13}C NMR (101 MHz, $CDCl_3$)** δ = 160.6, 157.0, 156.7, 131.8, 131.3, 131.1, 125.8, 125.1, 124.8, 119.4, 115.1, 114.8, 104.2, 69.70, 69.67, 51.4, 22.1; **HRMS**: Found $[M+Na]^+$ = 494.09372, $C_{24}H_{26}BrNO_4Na$ requires 494.09374, Δ–0.05 ppm.

Methyl 3,4-bis(4-isopropoxyphenyl)-1*H*-pyrrole-2-carboxylate (25)

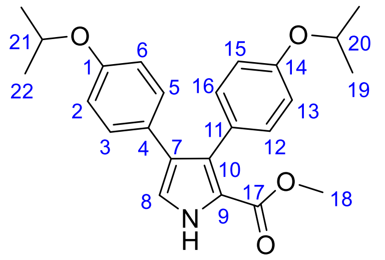

A mixture of methyl 5-bromo-3,4-bis(4-isopropoxyphenyl)-1*H*-pyrrole-2-carboxylate (**24**) (47.2 mg, 0.1 mmol, 1.0 eq.), NaOAc (20 mg, 0.21 mmol, 2.1 eq.) and 10 mol% Pd/C (16 mg) in MeOH (5 mL) was purged with H_2 and stirred under an atmosphere of H_2 (balloon) at rt for 1 h. The reaction mixture was filtered, and the filtrate was concentrated *in vacuo* to give the *title compound* **25** (33 mg, >99%) as colorless plates. **m.p.** 143-144 °C (*n*-pentane/Et_2O) [lit.[7] 141-141.5 °C (*n*-hexane/EtOAc)]; **IR** ν_{max} 3357 (NH), 2975 and 2939 (alkyl C-H), 1700, 1611, 1572, 1554, 1536, 1506, 1481, 1438, 1419, 1370, 1306, 1282, 1238, 1182, 1150, 1135, 1118, 1104, 1087, 1025, 1008, 947, 921, 859 cm^{-1}; **^{1}H NMR (400 MHz, $CDCl_3$)** δ = 9.12 (1H, br s, N*H*), 7.17 (2H, d, *J* = 8.7 Hz, HC_{Ar}), 7.02 (1H, d, *J* = 3.1 Hz, C(8)*H*), 7.00 (2H, d, *J* = 8.8 Hz, HC_{Ar}), 6.82 (2H, d, *J* = 8.7 Hz, HC_{Ar}), 6.72 (2H, d, *J* = 8.7 Hz, HC_{Ar}), 4.55 (1H, hept, *J* = 6.1 Hz, $OCH(CH_3)_2$), 4.48 (1H, hept, *J* = 6.1 Hz, $OCH(CH_3)_2$), 3.73 (3H, s, $OC(18)H_3$), 1.35 (6H, d, *J* = 6.0 Hz, $OCH(CH_3)_2$), 1.31 (6H, d, *J* = 6.1 Hz, $OCH(CH_3)_2$); **^{13}C NMR (101 MHz, $CDCl_3$)** δ = 161.5, 156.8, 156.3, 131.9, 129.4, 129.1, 126.8, 126.5, 126.1, 120.0, 119.3, 115.5, 115.0, 69.8, 69.7, 51.2, 22.14, 22.08; **HRMS**: Found $[M+Na]^+$ = 416.18304, $C_{24}H_{27}NO_4{}^{23}Na$, requires 416.18323, Δ–0.44 ppm. Spectroscopic data are consistent with those previously reported in the literature.[7]

Methyl 3,4-bis(4-hydroxyphenyl)-1*H*-pyrrole-2-carboxylate, Lamellarin Q

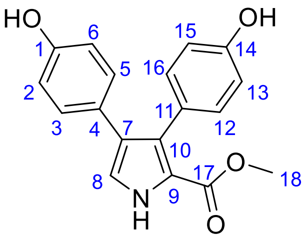

To a stirred solution of methyl 3,4-bis(4-isopropoxyphenyl)-1*H*-pyrrole-2-carboxylate (**25**) (20 mg, 0.06 mmol, 1.0 eq.) in DCM (5 mL) under argon atmosphere at –78 °C was added BBr_3 (1M in DCM, 0.2 mL, 0.18 mmol, 3.0 eq.). After complete addition the solution was left to warm to rt. After 3 h the reaction was quenched by the addition of saturated aq. $NaHCO_3$ (1 mL) and extracted with DCM (2 × 5 mL). The combined organic extracts were dried with $MgSO_4$, filtered and evaporated *in vacuo*. Flash column chromatography (SiO_2, *n*-pentane/acetone, 80:20) gave

lamellarin Q (16 mg, >99%) as unstable yellow needles. **m.p.** 218-221 °C with decomposition (lit.[8] 220-222 °C with decomposition). **^{1}H NMR (400 MHz, acetone-*d*$_6$)** δ = 10.88 (1H, br s, N*H*), 8.21 (1H, s, O*H*), 8.13 (1H, s, O*H*), 7.13 (1H, d, *J* = 3.2 Hz, C(8)*H*), 7.05 (2H, d, *J* = 8.6 Hz, *H*C$_{Ar}$), 6.95 (2H, d, *J* = 8.6 Hz, *H*C$_{Ar}$), 6.75 (2H, d, *J* = 8.5 Hz, *H*C$_{Ar}$), 6.66 (2H, d, *J* = 8.6 Hz, *H*C$_{Ar}$), 3.64 (3H, s, OC(8)*H*$_3$). **^{13}C NMR (100 MHz, acetone-*d*$_6$)** 161.9, 157.0, 156.5, 132.8, 130.2, 129.7, 127.4, 126.93, 126.88, 121.4, 120.0, 115.8, 115.2, 51.0; **HRMS** (ESI$^+$) Found [M+H]$^+$ = 310.10748; $C_{18}H_{16}O_4N$ requires 310.10739, Δ+0.32 ppm. Physical and spectroscopic data are consistent with those previously reported.[8]

S4. ^{13}C NMR Data Lamellarin D and Q Compared to Previously Reported Data

Table 1. Comparison of ^{13}C NMR data of lamellarin D in DMSO-d_6 with a synthetic sample reported by Jia *et al.*[5]

Literature δ_C[5]	This report δ_C	$\Delta\delta_C$
54.5	54.6	+0.1
55.1	55.1	
56.0	56.0	
103.7	103.8	+0.1
105.4	105.4	
105.8	105.8	
106.4	106.4	
108.3	108.4	+0.1
110.7	110.9	+0.2
111.5	111.6	+0.1
112.3	112.4	+0.1
115.1	115.1	
116.4	116.5	+0.1
117.5	117.6	+0.1
121.9	122.1	+0.2
123.8	123.9	+0.1
124.6	124.7	+0.1
125.5	125.5	
128.9	129.0	+0.1
134.0	134.1	+0.1
144.5	144.6	+0.1
146.3	146.4	+0.1
146.8	146.9	+0.1
147.8	147.9	+0.1
148.2	148.4	+0.2
148.4	148.5	+0.1
148.7	148.7	
154.3	154.4	+0.1

Table 2. Comparison of ^{13}C NMR data of lamellarin Q in acetone-d_6 with a synthetic sample reported by Vazquez *et al.*[8]

Literature δ_C[8]	This report δ_C	$\Delta\delta_C$
**161.9**	161.9	
**157.0**	157.0	
**156.5**	156.5	
**132.8**	132.8	
**130.2**	130.2	
**129.7**	129.7	
**127.4**	127.4	
**127.0**	126.9	-0.1
**126.8**	126.9	+0.1
**121.4**	121.4	
**120.0**	120.0	
**115.9**	115.8	-0.1
**115.2**	115.2	
**51.0**	51.0	

S5. References

1. N. Fujikawa, T. Ohta, T. Yamaguchi, T. Fukuda, F. Ishibashi, M. Iwao, *Tetrahedron*, 2006, **62**, 594.
2. J. A. Gladding, J.P. Bacci, S. A. Shaw, A. B. Smith, *Tetrahedron*, 2011, **67**, 6697.
3. H. A. Esteves, C. J. J. Hall, P. D. Smith and T. J. Donohoe, *Org. Lett.*, 2017, **19**, 5248.
4. M. Andreini, E. Gabellieri, W. Guba, G. Marconi, R. Narquizian, E. Power, M. Travagli, T. Woltering, W. Wostl, *US Patent*, US2009/209529, A1, 2009.
5. Q. Li, J. Jiang, A. Fan, Y. Cui, Y. Jia, *Org. Lett.*, 2011, **13**, 312.
6. T. Ohta, T. Fukuda, F. Ishibashi, M. Iwao, *J. Org. Chem.*, 2009, **74**, 8143.
7. T. Fukuda, E.-I. Sudo, K. Shimokawa, M. Iwao, *Tetrahedron*, 2008, **64**, 328.
8. A. Ramírez-Rodríguez, J. M. Méndez, C.C. Jiménez, F. León, A. Vazquez, *Synthesis*, 2012, **44**, 3321.

S6. NMR Spectra

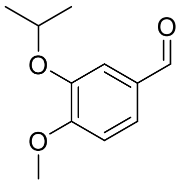

S1

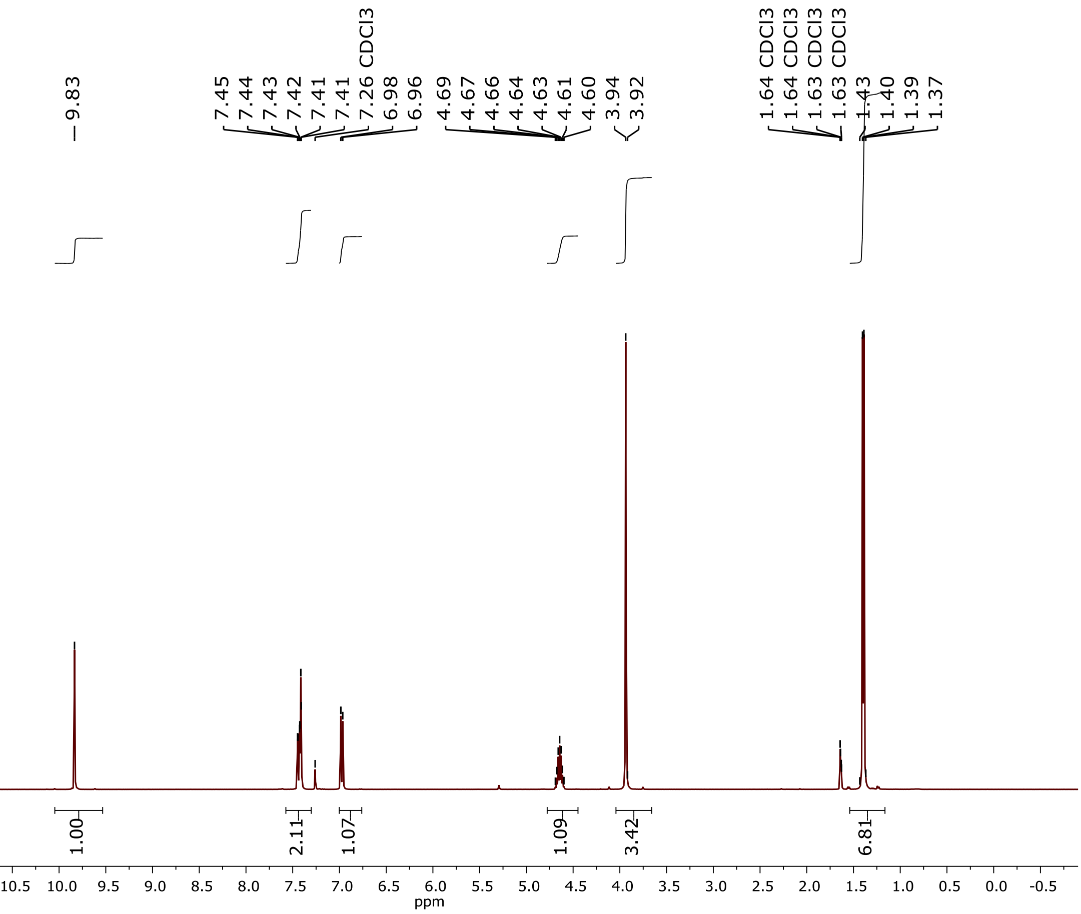

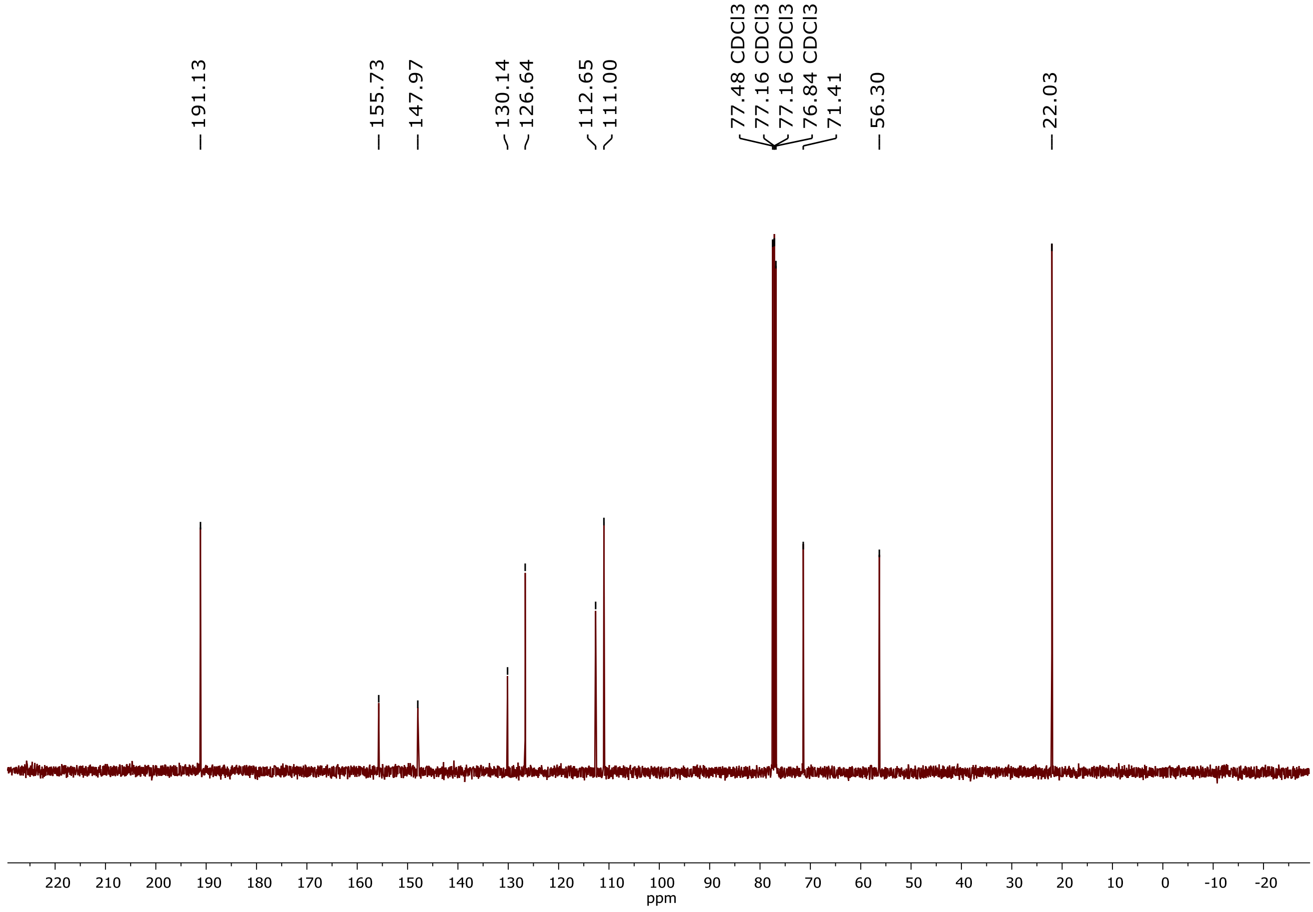

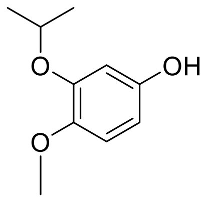

S2

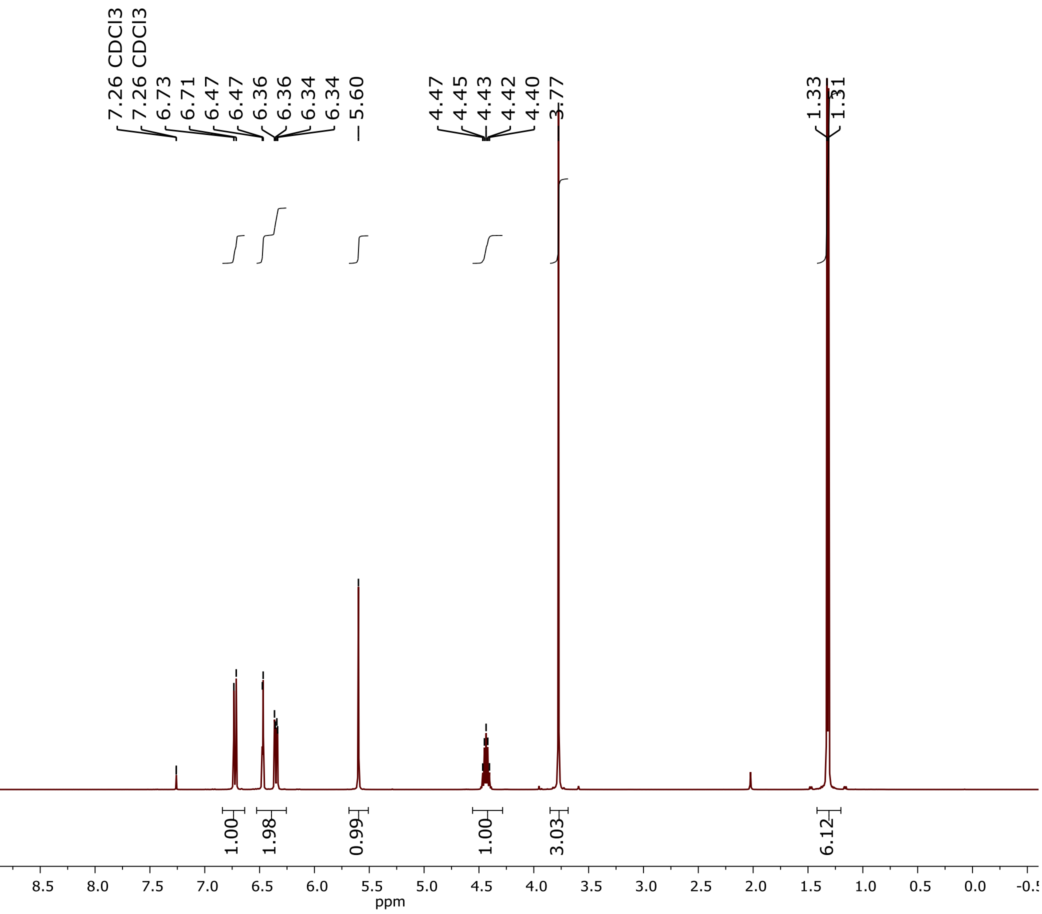
7.26 CDCl3
7.26 CDCl3
6.73
6.71
6.47
6.47
6.36
6.36
6.34
6.34
5.60
4.47
4.45
4.43
4.42
4.40
3.77
1.33
1.31
1.00
1.98
0.99
1.00
3.03
6.12
11.0 10.5 10.0 9.5 9.0 8.5 8.0 7.5 7.0 6.5 6.0 5.5 5.0 4.5 4.0 3.5 3.0 2.5 2.0 1.5 1.0 0.5 0.0
ppm

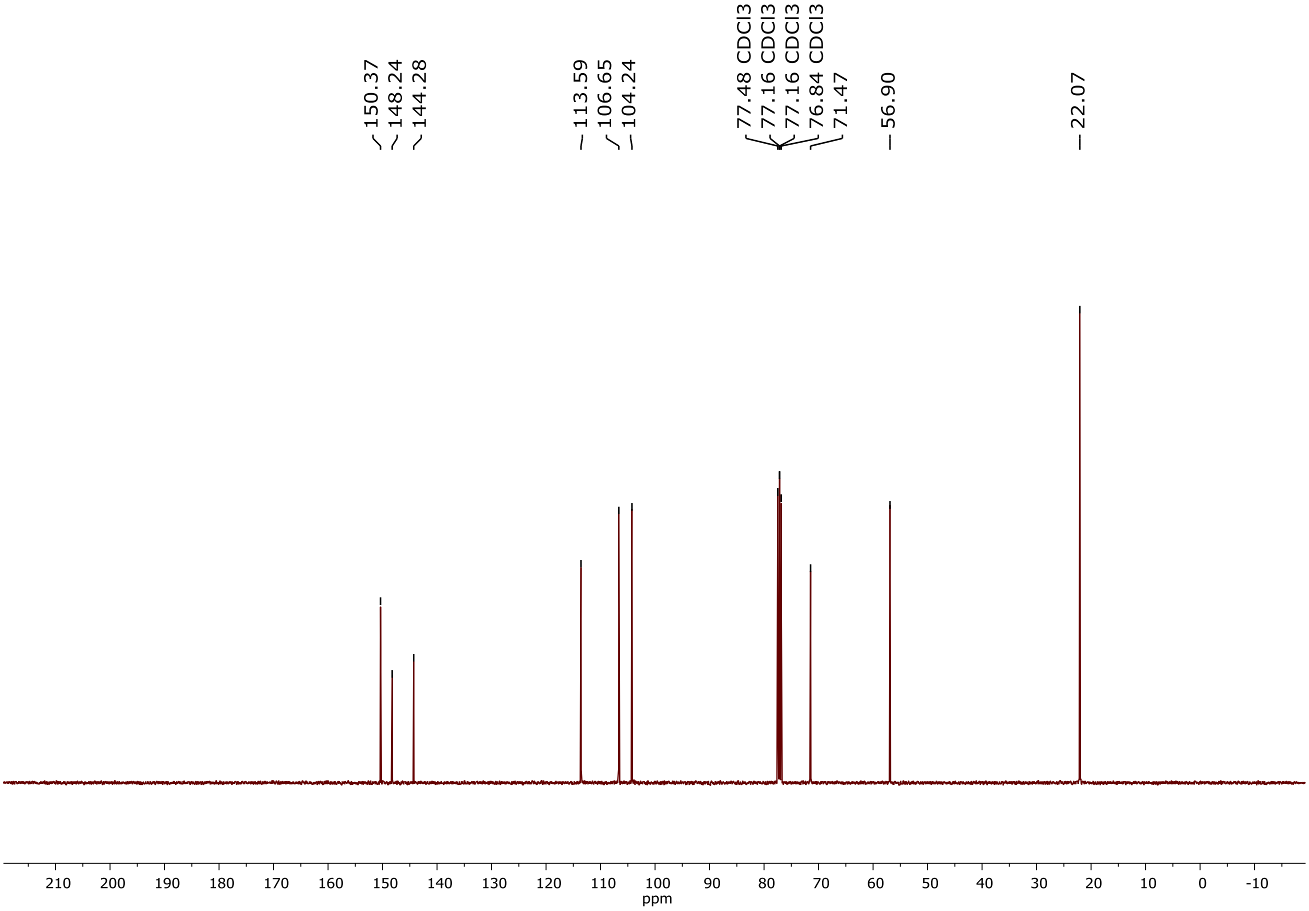
150.37
148.24
144.28
113.59
106.65
104.24
77.48 CDCl3
77.16 CDCl3
77.16 CDCl3
76.84 CDCl3
71.47
56.90
22.07
210 200 190 180 170 160 150 140 130 120 110 100 90 80 70 60 50 40 30 20 10 0 -10
ppm

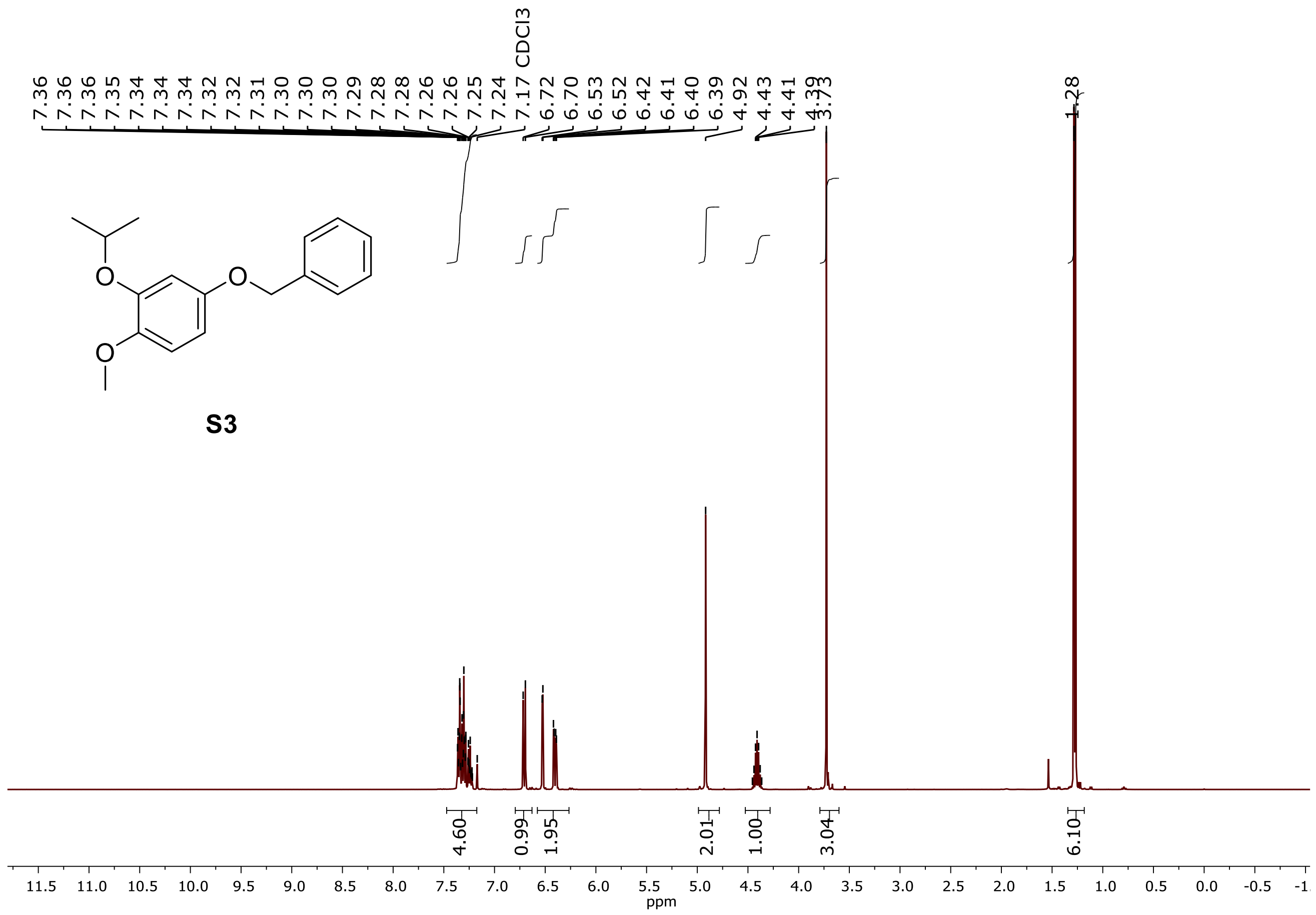
7.36
7.36
7.36
7.35
7.34
7.34
7.34
7.32
7.32
7.31
7.30
7.30
7.30
7.29
7.28
7.28
7.26
7.26
7.25
7.24
7.17 CDCl3
6.72
6.70
6.53
6.52
6.42
6.41
6.40
6.39
4.92
4.43
4.41
4.39
3.73
1.28
S3
4.60
0.99
1.95
2.01
1.00
3.04
6.10
ppm

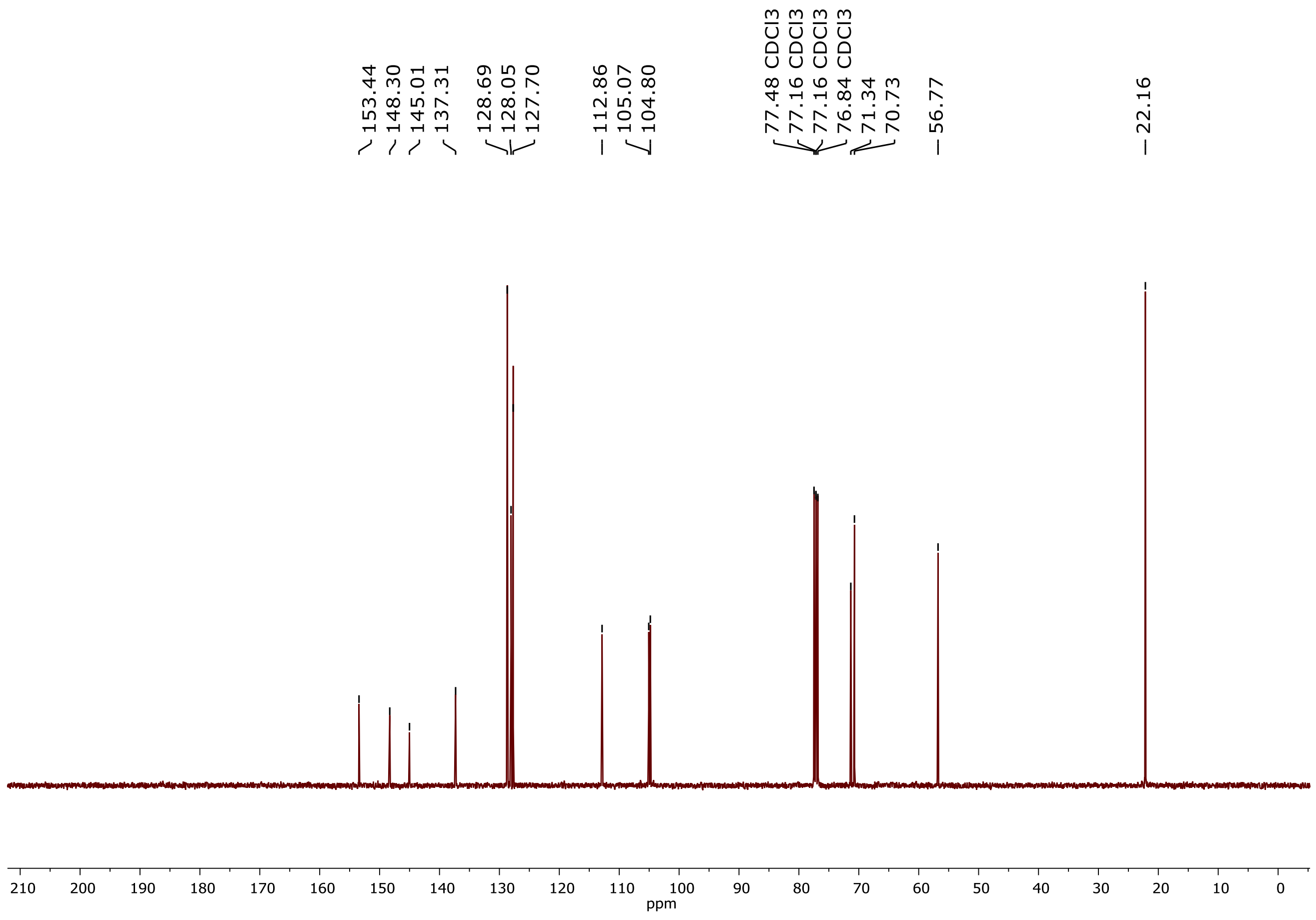
153.44
148.30
145.01
137.31
128.69
128.05
127.70
112.86
105.07
104.80
77.48 CDCl3
77.16 CDCl3
77.16 CDCl3
76.84 CDCl3
71.34
70.73
56.77
22.16
ppm

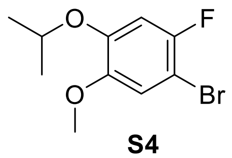
O
F
O
Br

S4

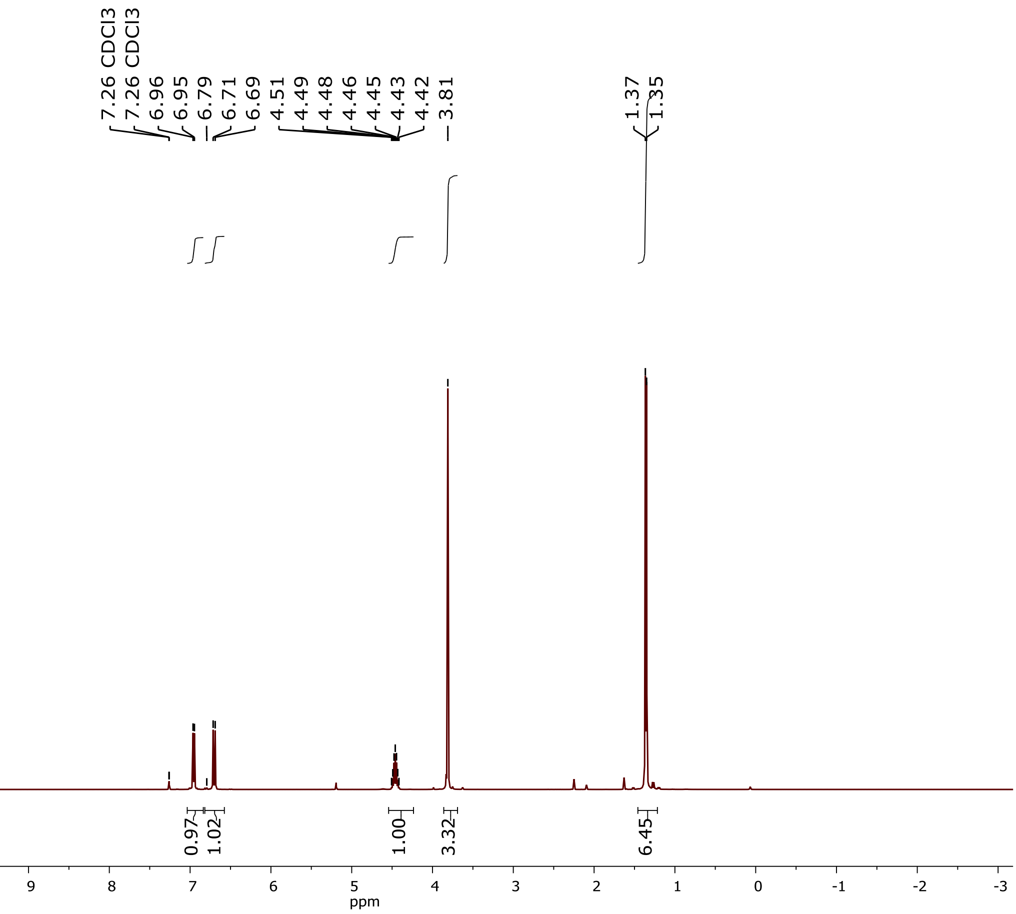
7.26 CDCl3
7.26 CDCl3
6.96
6.95
6.79
6.71
6.69
4.51
4.49
4.48
4.46
4.45
4.43
4.42
3.81
1.37
1.35
0.97
1.02
1.00
3.32
6.45
12
11
10
9
8
7
6
5
4
3
2
1
0
-1
-2
-3
ppm

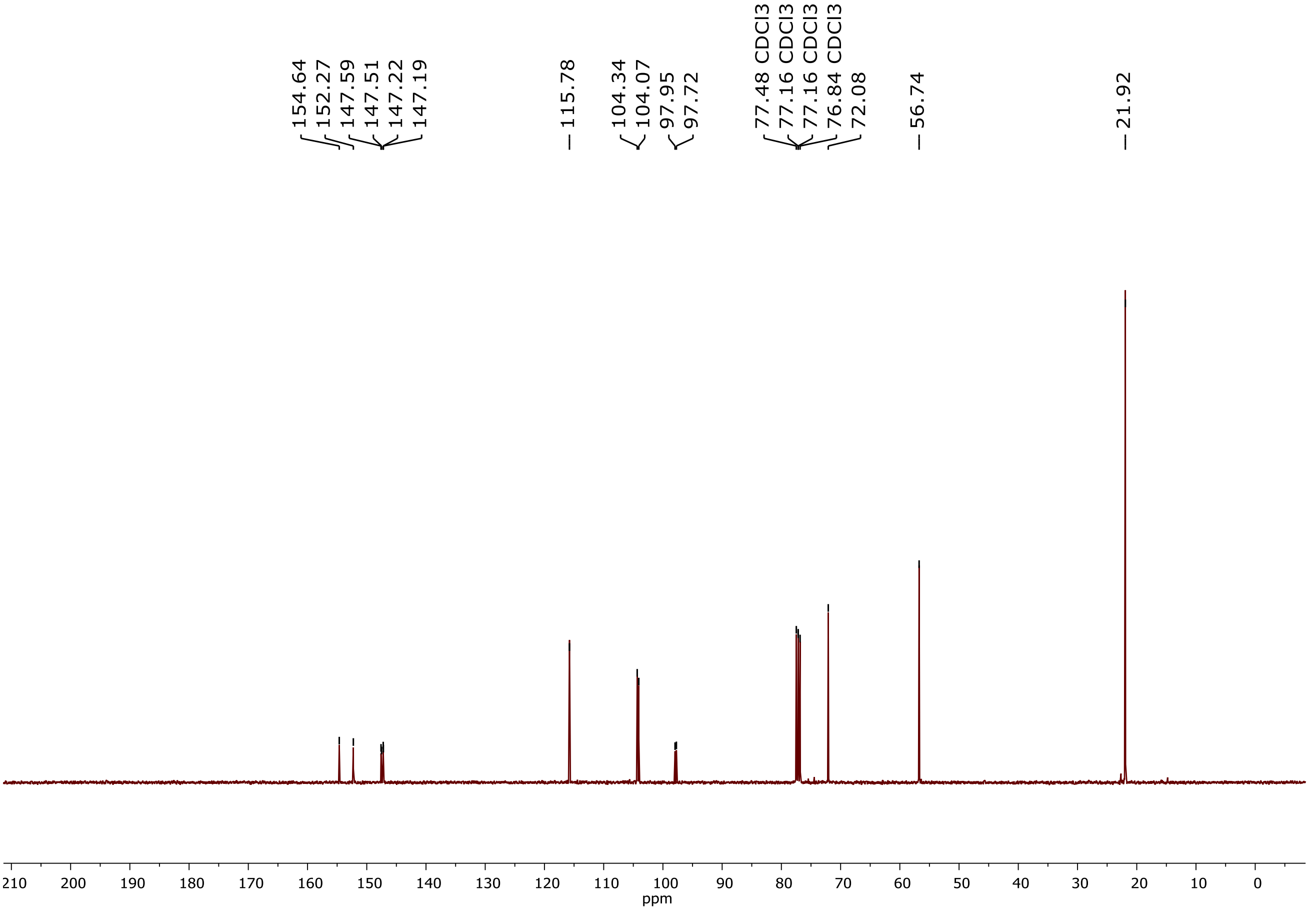
154.64
152.27
147.59
147.51
147.22
147.19
115.78
104.34
104.07
97.95
97.72
77.48 CDCl3
77.16 CDCl3
77.16 CDCl3
76.84 CDCl3
72.08
56.74
21.92
210
200
190
180
170
160
150
140
130
120
110
100
90
80
70
60
50
40
30
20
10
0
ppm

[19]F

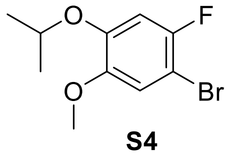

S4

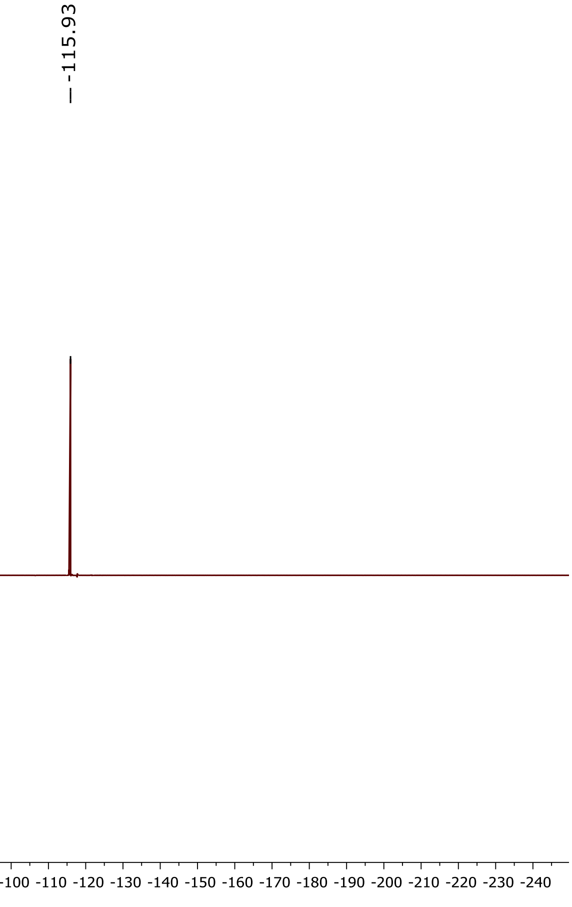
-115.93
90 80 70 60 50 40 30 20 10 0 -10 -20 -30 -40 -50 -60 -70 -80 -90 -100 -110 -120 -130 -140 -150 -160 -170 -180 -190 -200 -210 -220 -230 -240
ppm

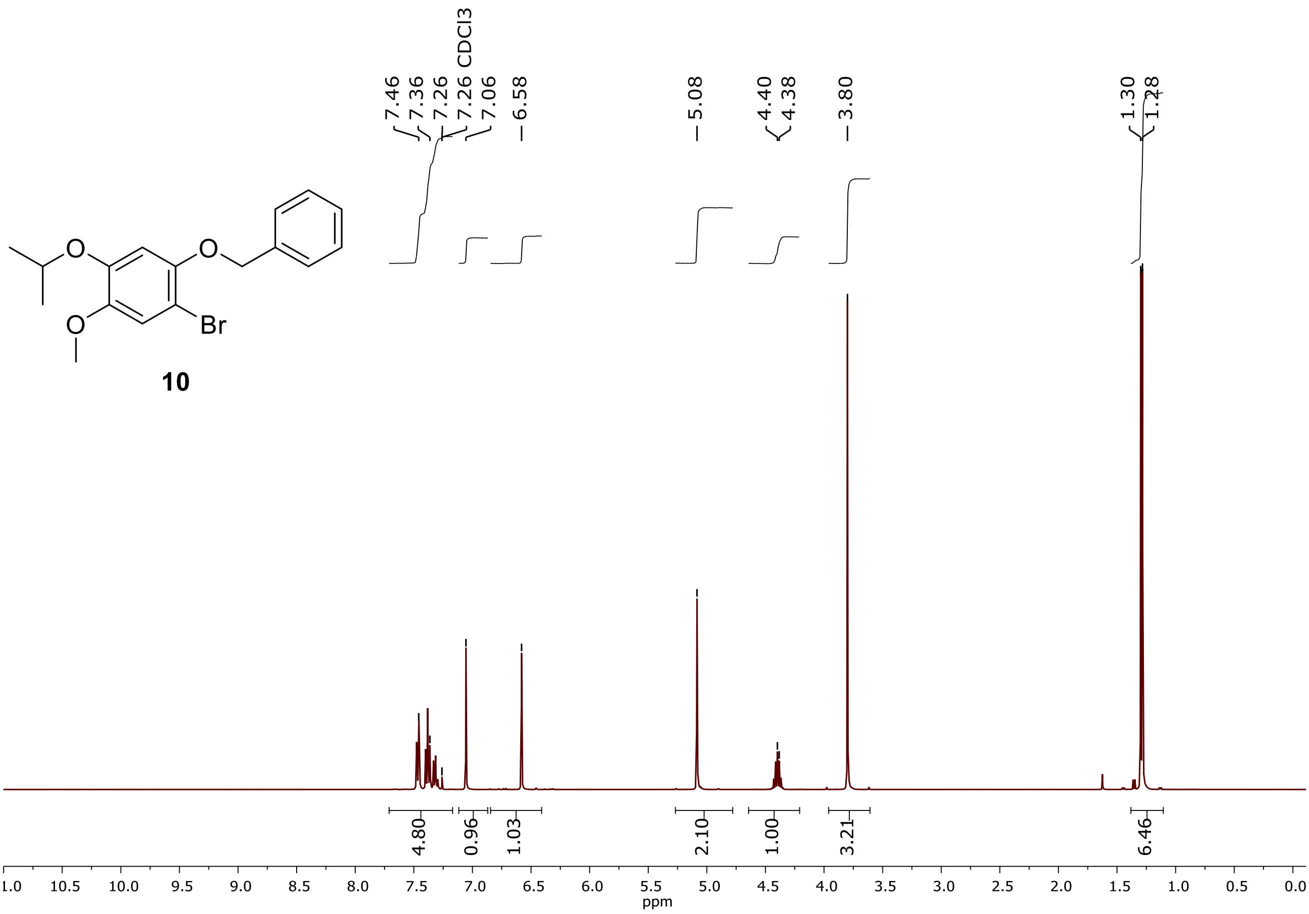
10
7.46
7.36
7.26
7.26 CDCl3
7.06
6.58
5.08
4.40
4.38
3.80
1.30
1.28
4.80
0.96
1.03
2.10
1.00
3.21
6.46
ppm

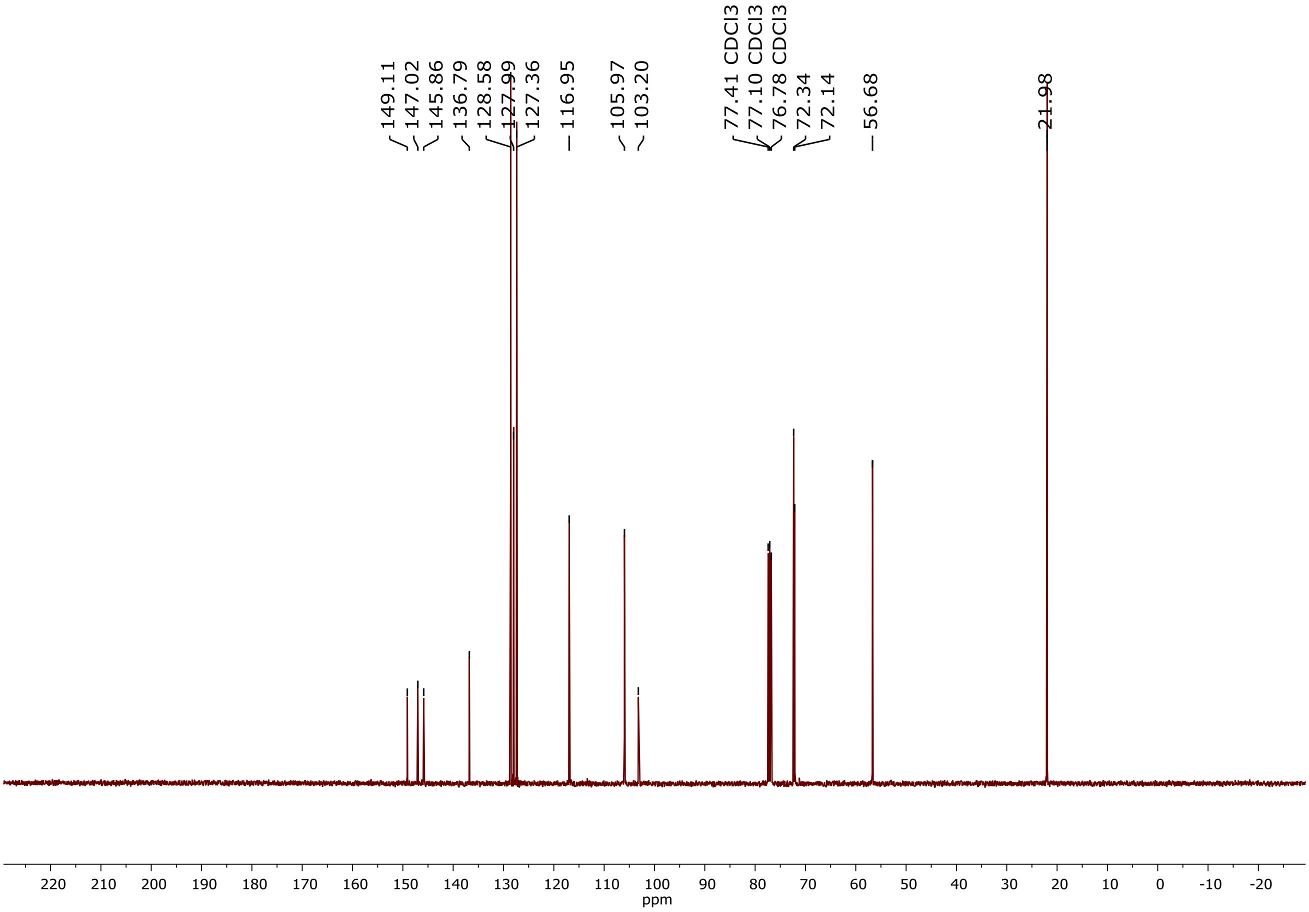
149.11
147.02
145.86
136.79
128.58
127.99
127.36
116.95
105.97
103.20
77.41 CDCl3
77.10 CDCl3
76.78 CDCl3
72.34
72.14
56.68
21.98
ppm

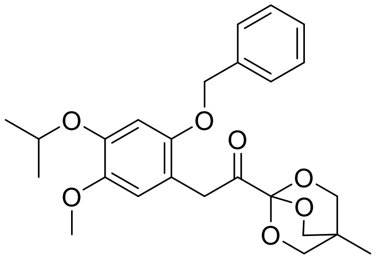

9

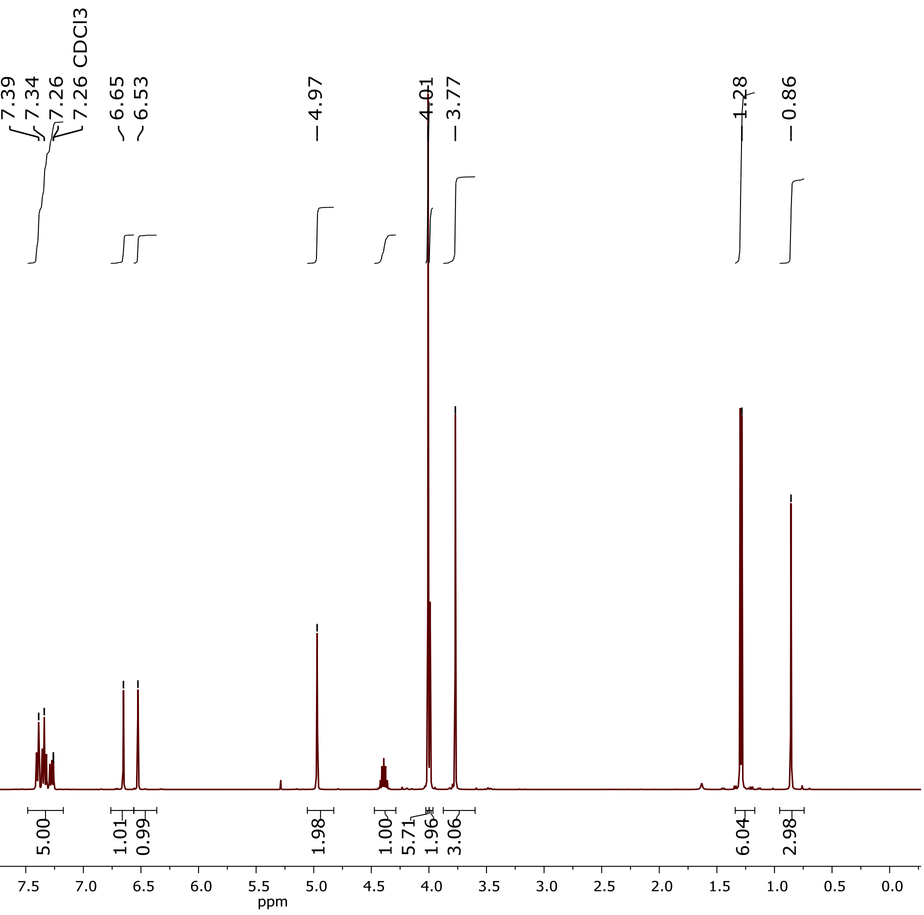
7.39
7.34
7.26
7.26 CDCl3
6.65
6.53
4.97
4.01
3.77
1.28
0.86
5.00
1.01
0.99
1.98
1.00
5.71
1.96
3.06
6.04
2.98
ppm

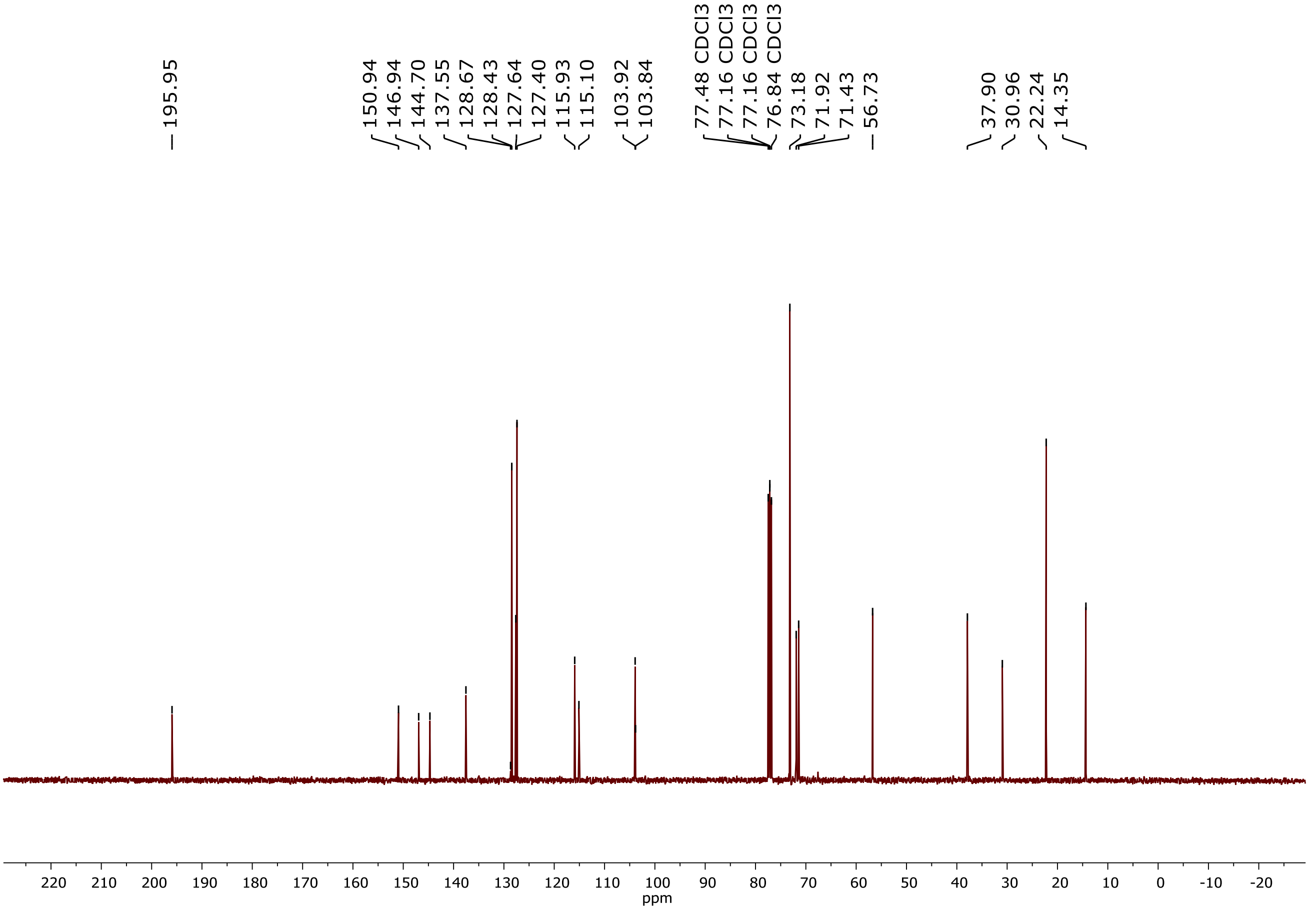
195.95
150.94
146.94
144.70
137.55
128.67
128.43
127.64
127.40
115.93
115.10
103.92
103.84
77.48 CDCl3
77.16 CDCl3
77.16 CDCl3
76.84 CDCl3
73.18
71.92
71.43
56.73
37.90
30.96
22.24
14.35
ppm

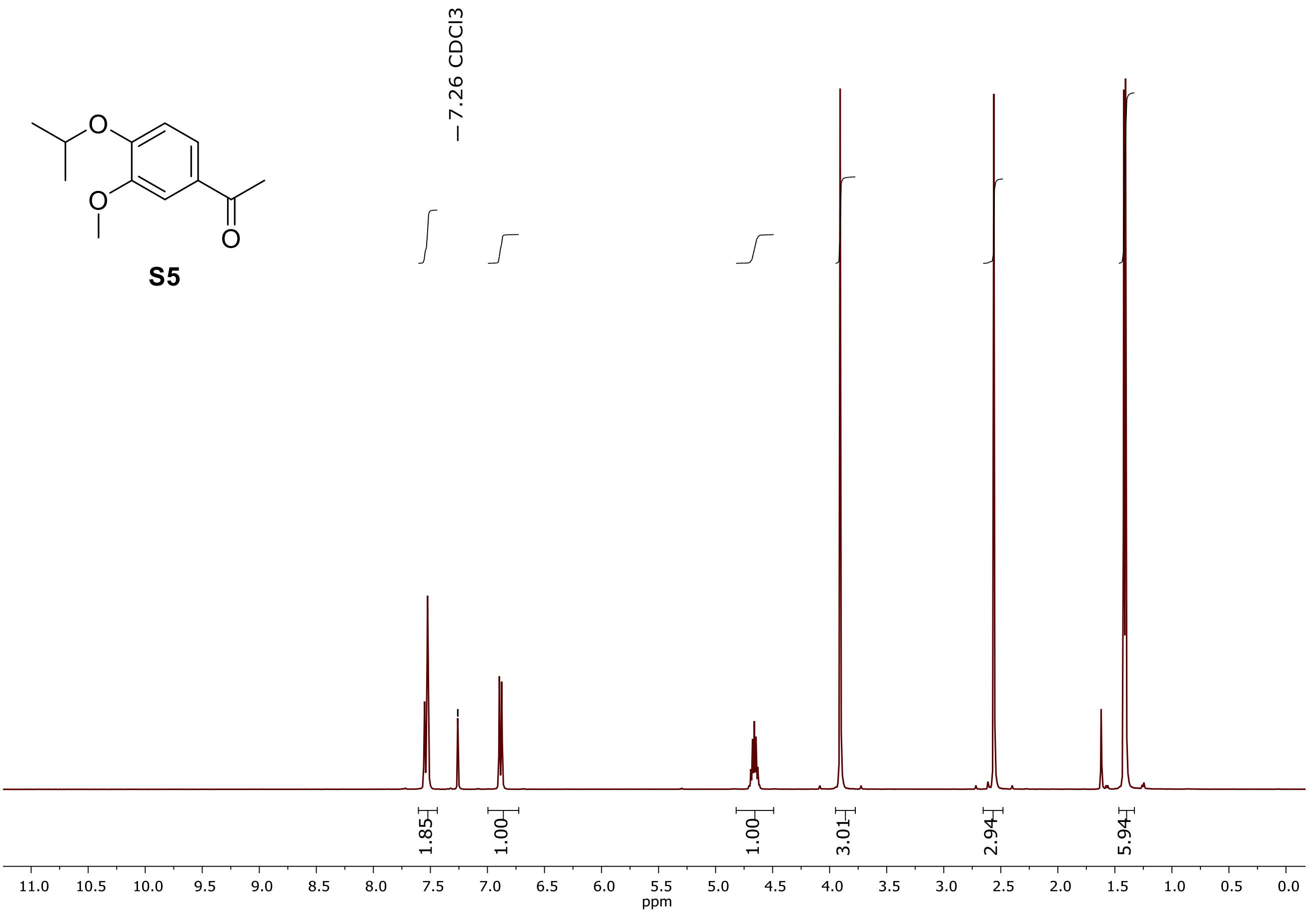
O
O
O
S5
7.26 CDCl3
1.85
1.00
1.00
3.01
2.94
5.94
11.0 10.5 10.0 9.5 9.0 8.5 8.0 7.5 7.0 6.5 6.0 5.5 5.0 4.5 4.0 3.5 3.0 2.5 2.0 1.5 1.0 0.5 0.0
ppm

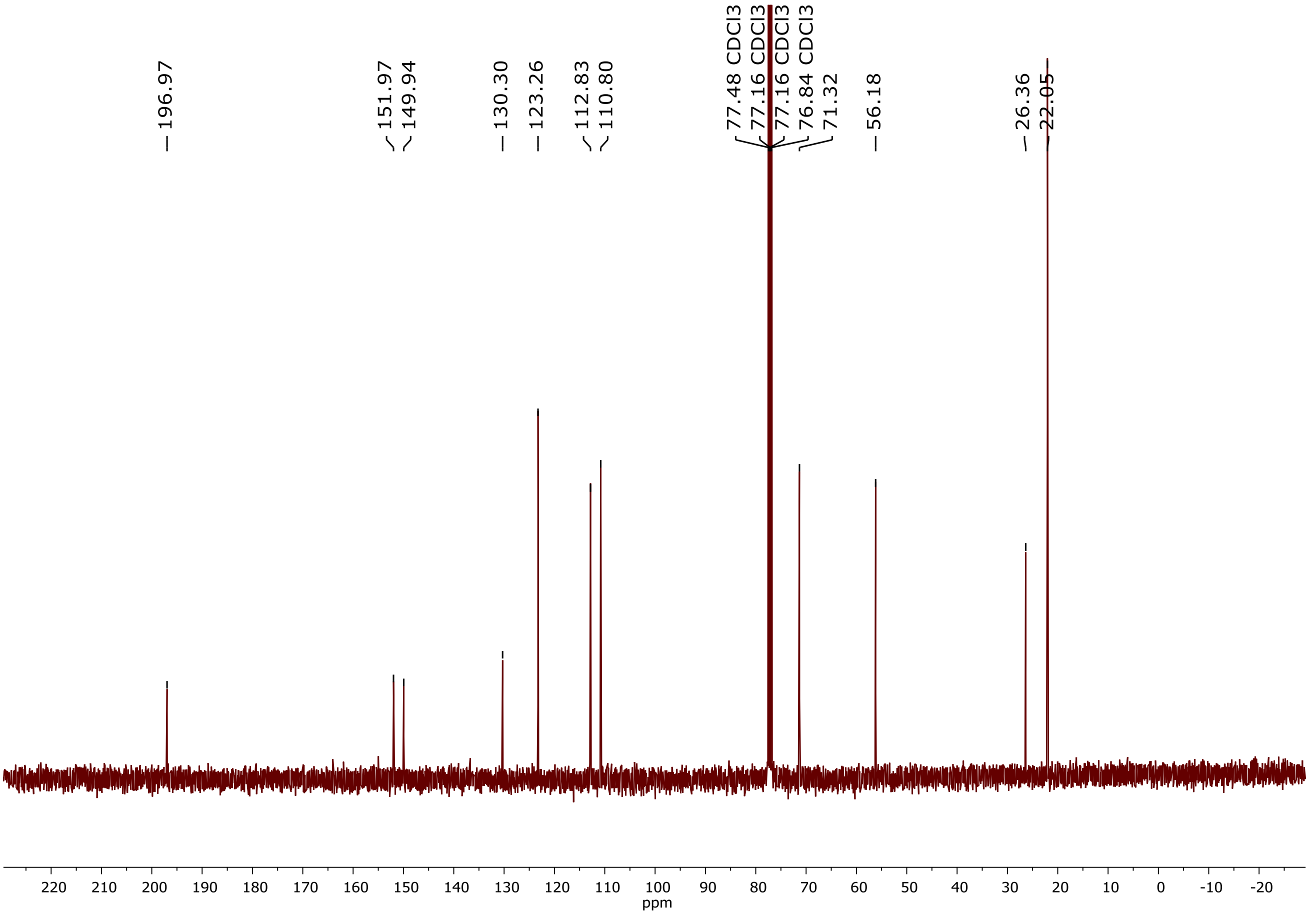
196.97
151.97
149.94
130.30
123.26
112.83
110.80
77.48 CDCl3
77.16 CDCl3
77.16 CDCl3
76.84 CDCl3
71.32
56.18
26.36
22.05
220 210 200 190 180 170 160 150 140 130 120 110 100 90 80 70 60 50 40 30 20 10 0 -10 -20
ppm

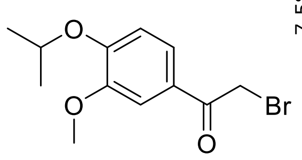
Br
O
O
O

8

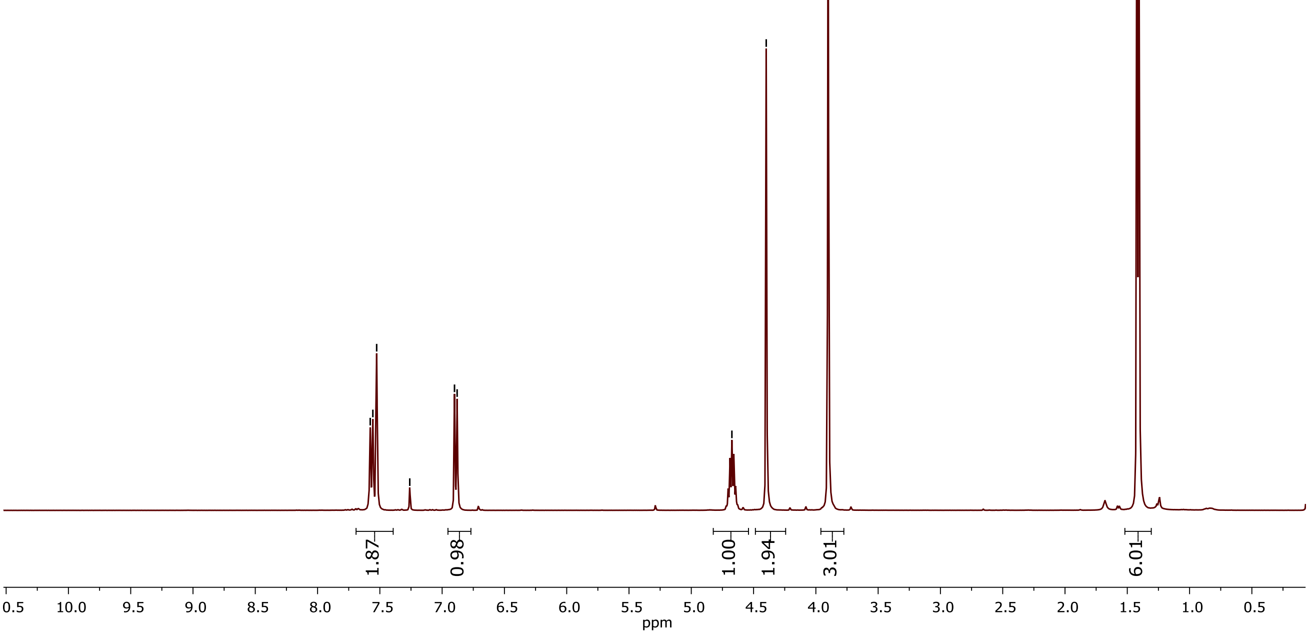
7.58
7.56
7.53
7.26 CDCl3
6.90
6.88
4.67
4.40
3.90
1.42
1.41
1.87
0.98
1.00
1.94
3.01
6.01
ppm

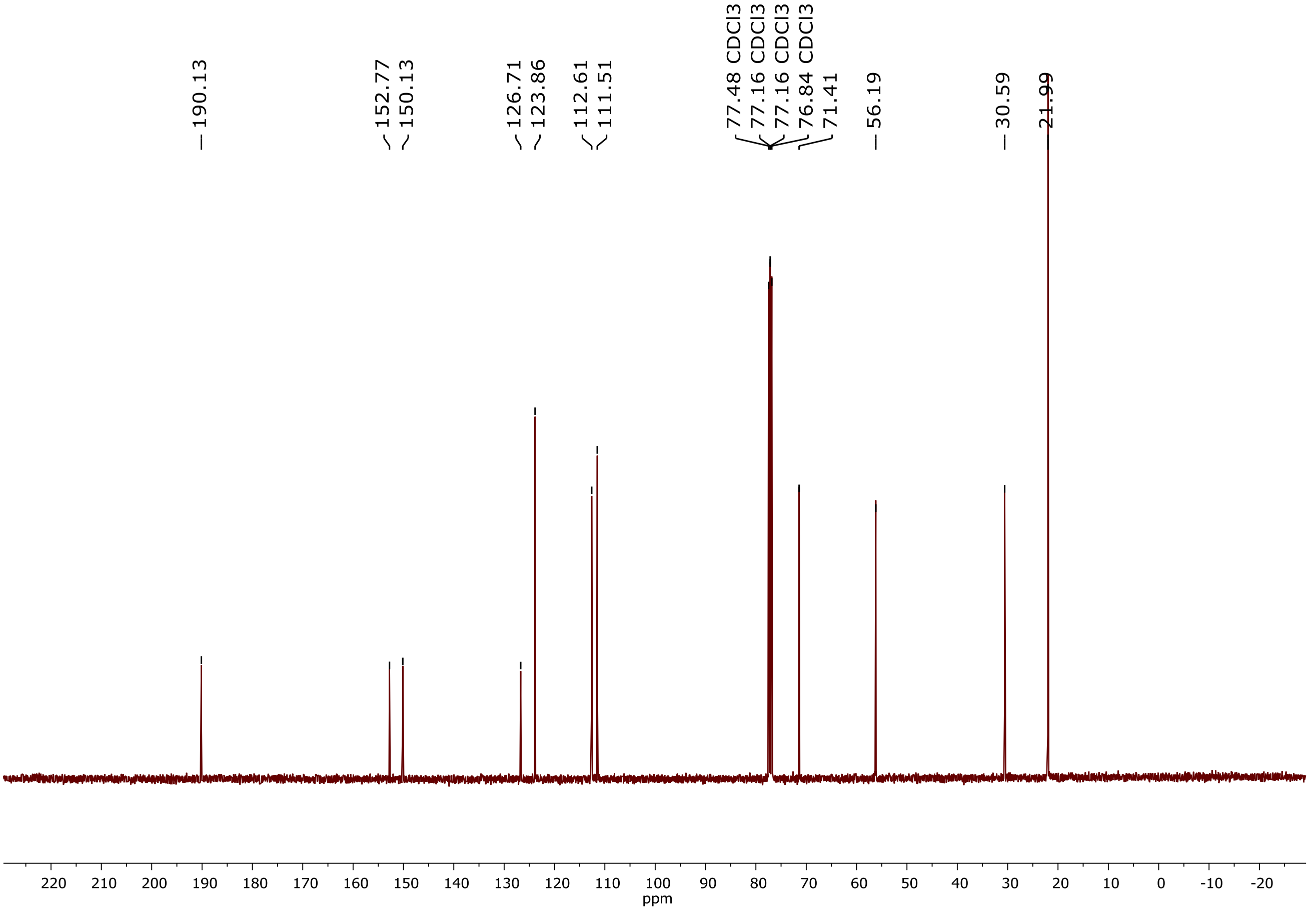
190.13
152.77
150.13
126.71
123.86
112.61
111.51
77.48 CDCl3
77.16 CDCl3
77.16 CDCl3
76.84 CDCl3
71.41
56.19
30.59
21.99
ppm

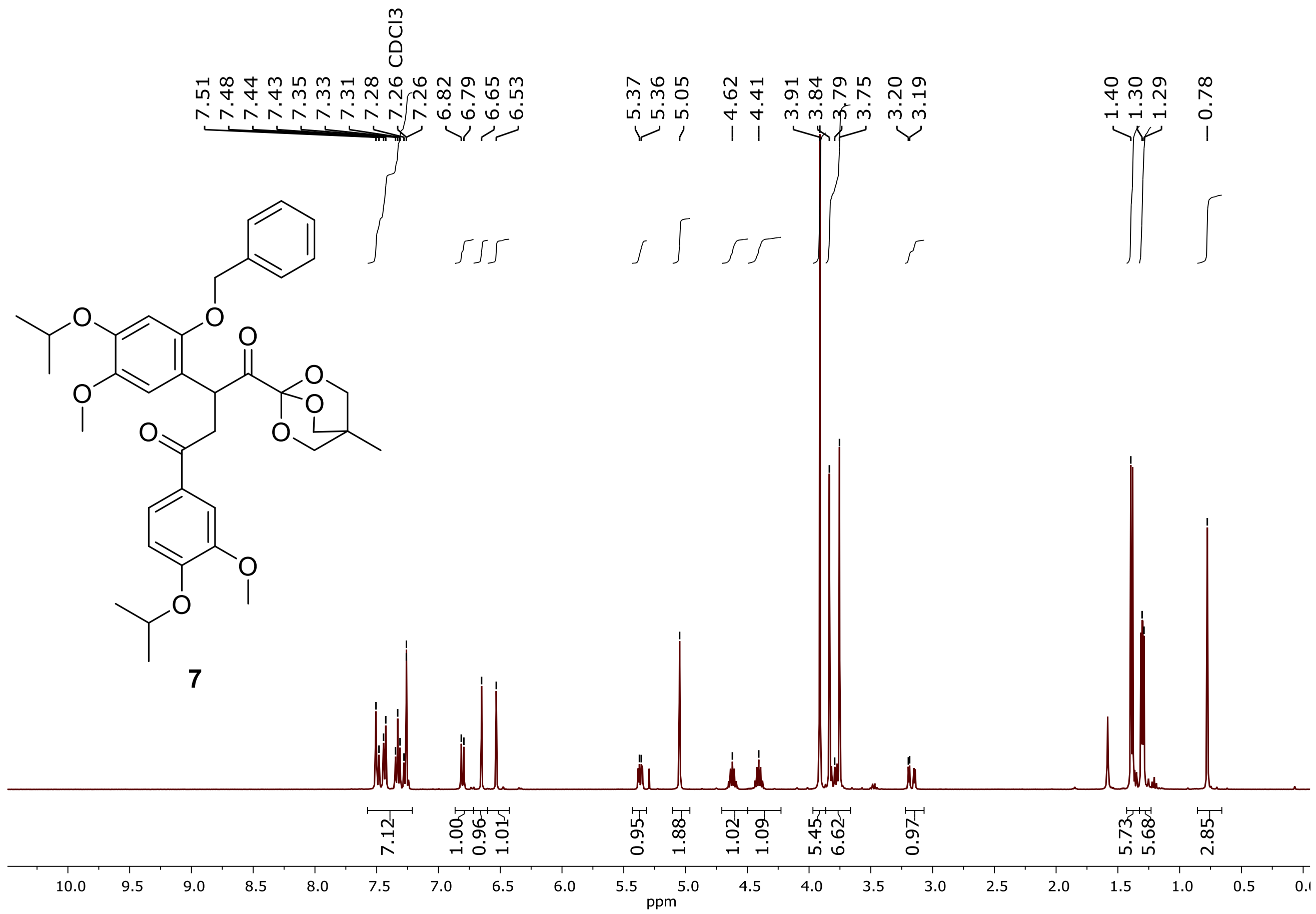
7.51
7.48
7.44
7.43
7.35
7.33
7.31
7.28
7.26 CDCl3
7.26
6.82
6.79
6.65
6.53
5.37
5.36
5.05
4.62
4.41
3.91
3.84
3.79
3.75
3.20
3.19
1.40
1.30
1.29
0.78
7
7.12
1.00
0.96
1.01
0.95
1.88
1.02
1.09
5.45
6.62
0.97
5.73
5.68
2.85
ppm

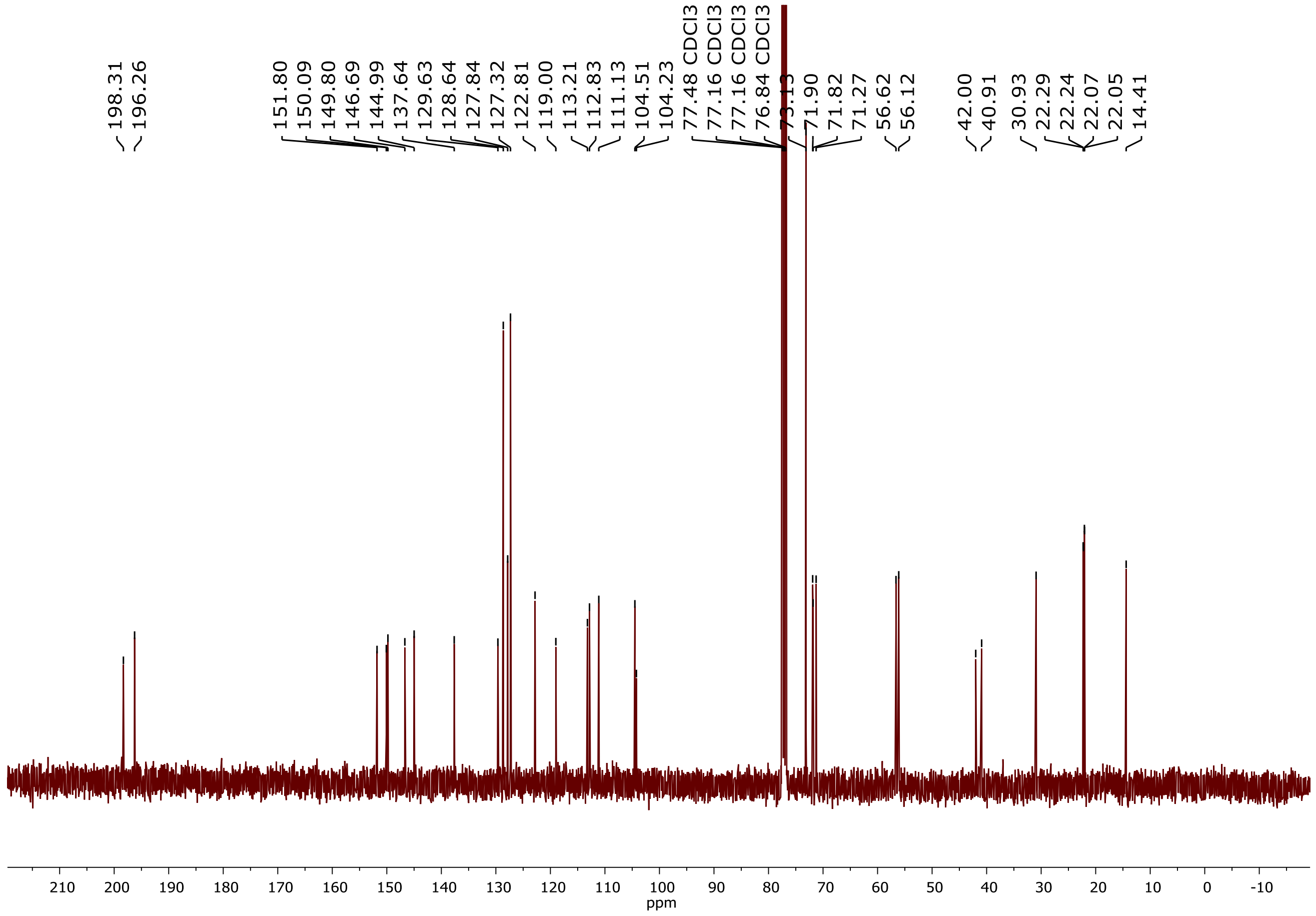
198.31
196.26
151.80
150.09
149.80
146.69
144.99
137.64
129.63
128.64
127.84
127.32
122.81
119.00
113.21
112.83
111.13
104.51
104.23
77.48 CDCl3
77.16 CDCl3
77.16 CDCl3
76.84 CDCl3
73.13
71.90
71.82
71.27
56.62
56.12
42.00
40.91
30.93
22.29
22.24
22.07
22.05
14.41
ppm

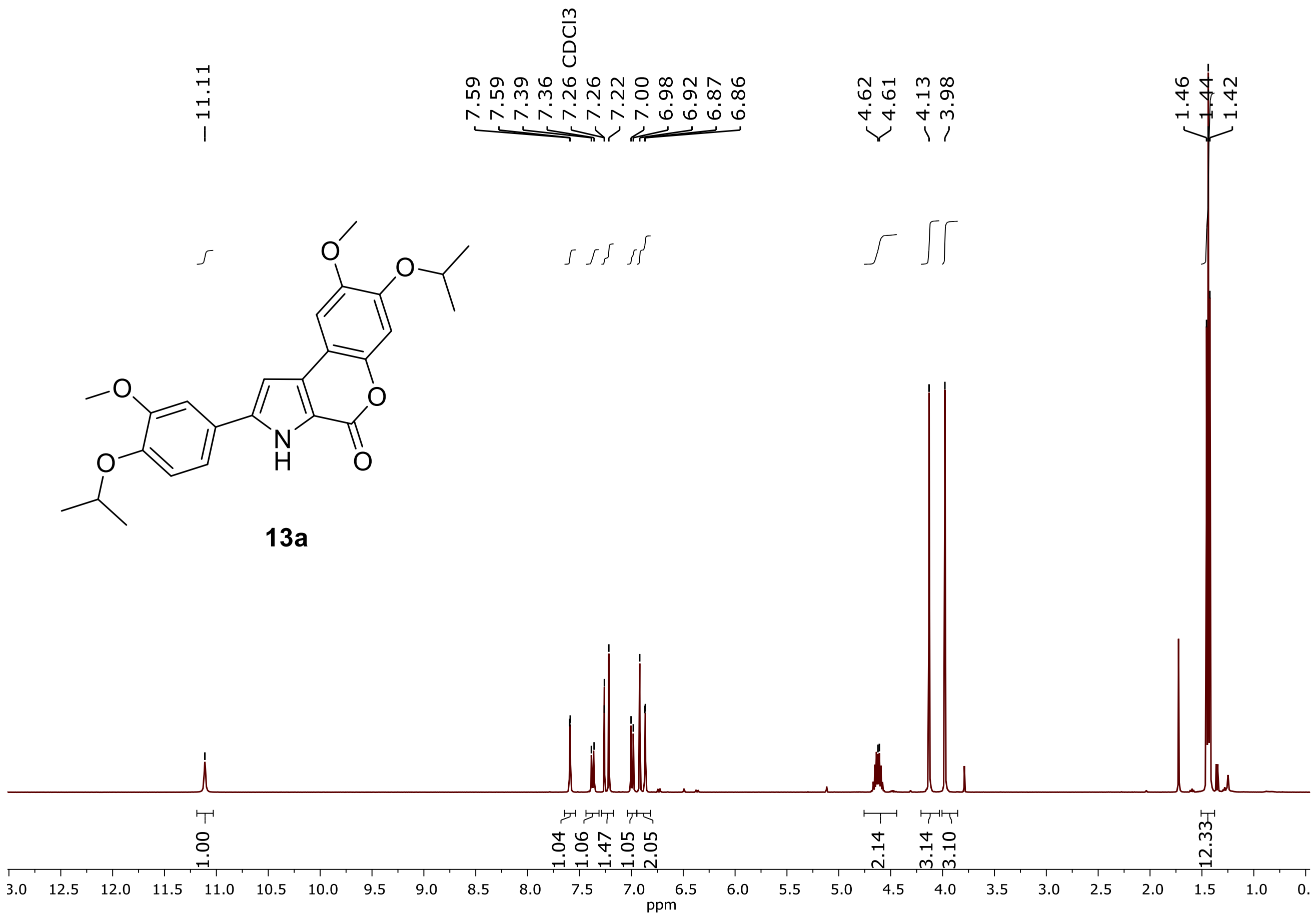
11.11
7.59 7.59 7.39 7.36 7.26 CDCl3 7.26 7.22 7.00 6.98 6.92 6.87 6.86
4.62 4.61 4.13 3.98
1.46 1.44 1.42
O
O
O
O
O
O
N
H
13a
1.00 1.04 1.06 1.47 1.05 2.05 2.14 3.14 3.10 12.33
ppm

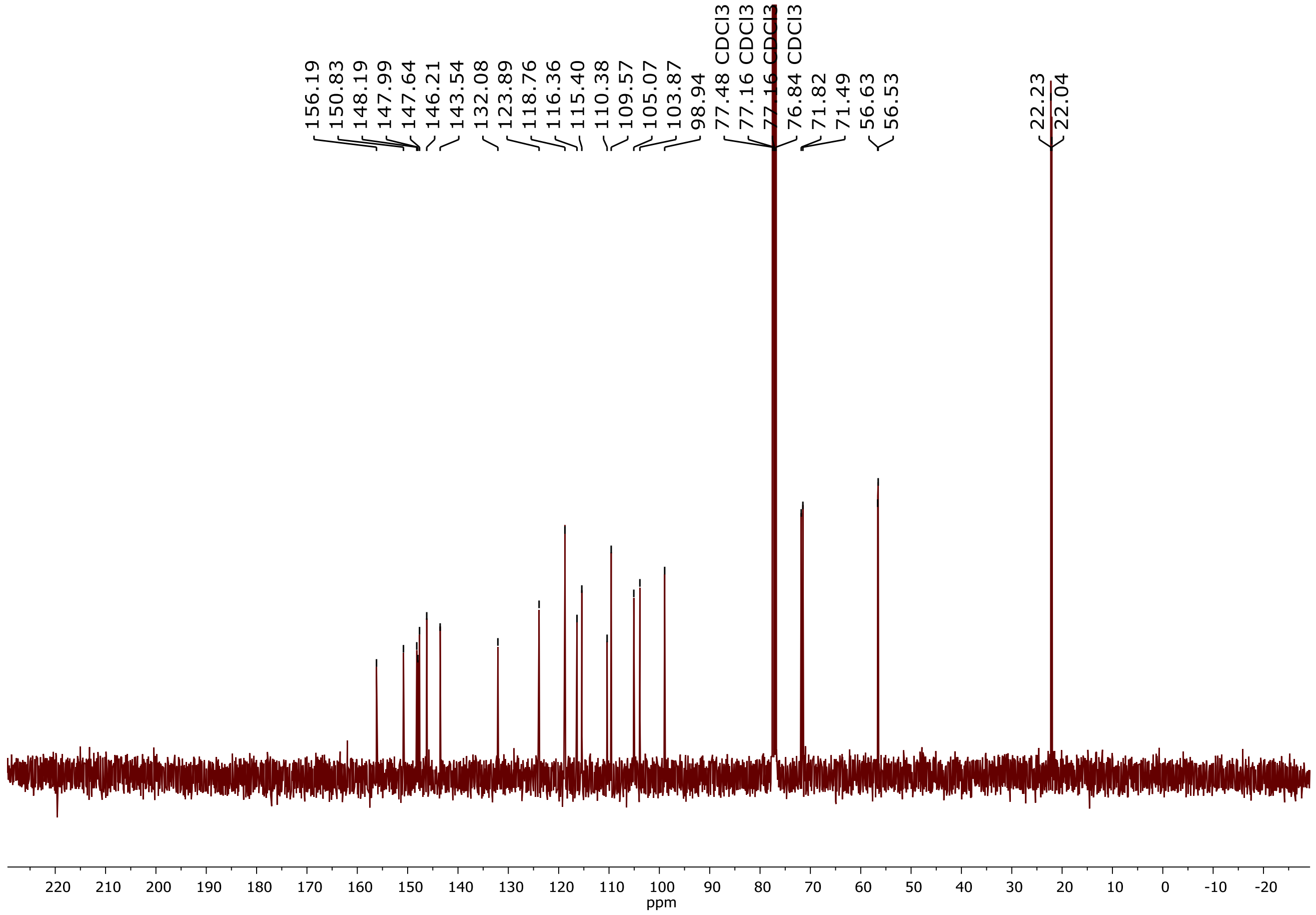
156.19 150.83 148.19 147.99 147.64 146.21 143.54 132.08 123.89 118.76 116.36 115.40 110.38 109.57 105.07 103.87 98.94 77.48 CDCl3 77.16 CDCl3 77.16 CDCl3 76.84 CDCl3 71.82 71.49 56.63 56.53
22.23 22.04
ppm

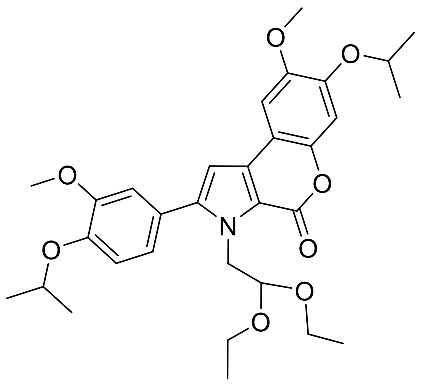

13b
unstable oil

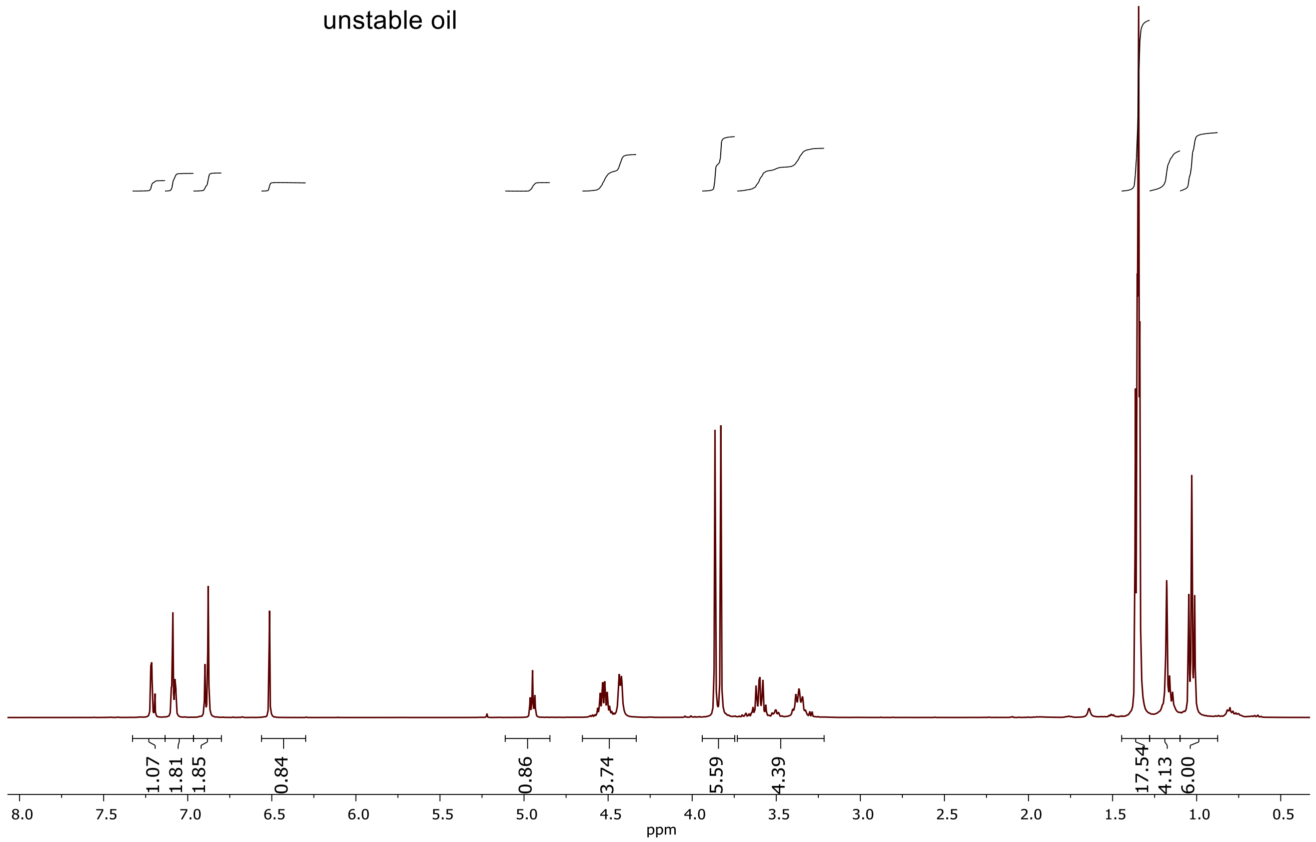

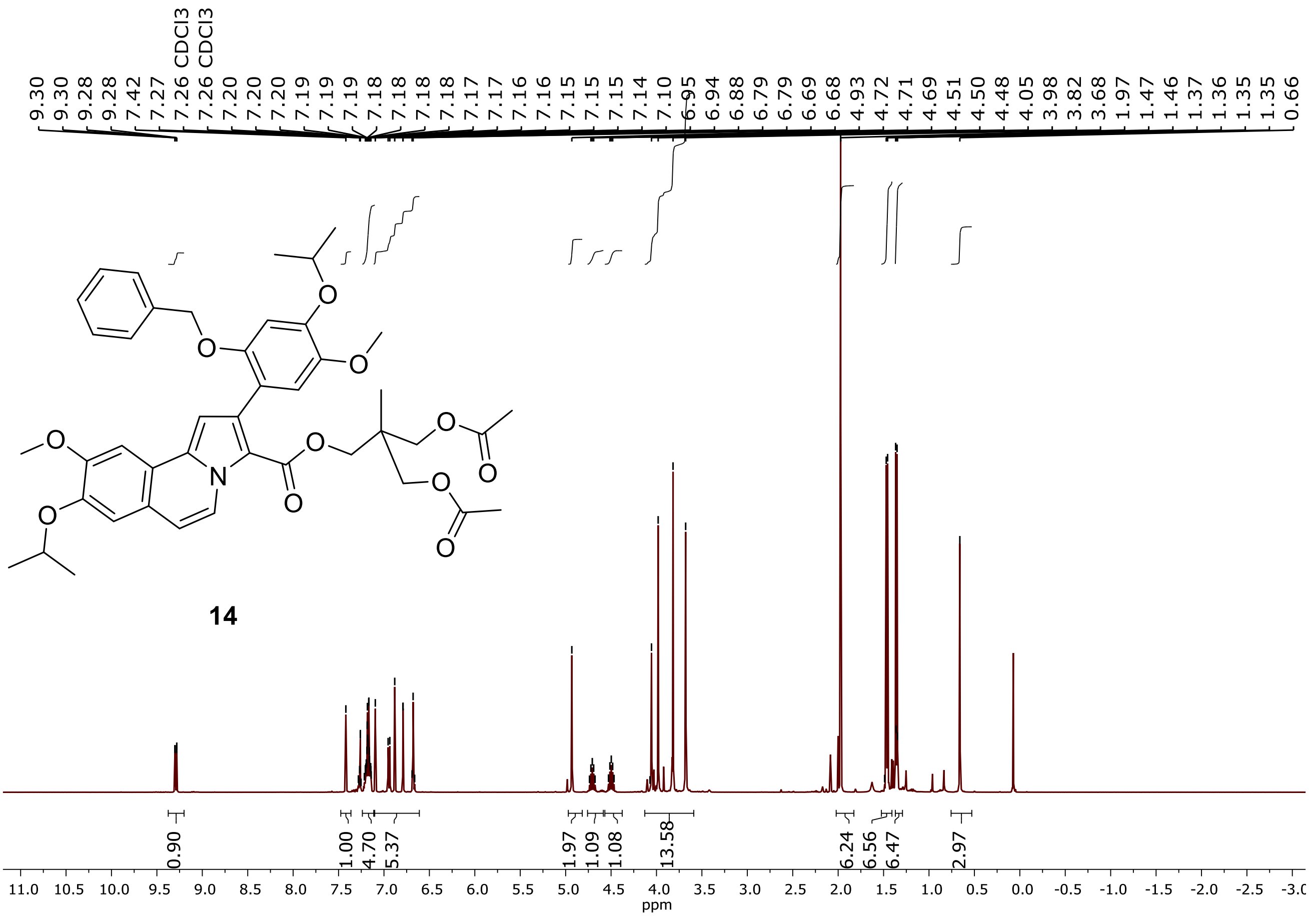
9.30 9.30 9.28 9.28 7.42 7.27 7.26 CDCl3 7.26 CDCl3 7.20 7.20 7.20 7.19 7.19 7.19 7.18 7.18 7.18 7.18 7.17 7.17 7.16 7.16 7.15 7.15 7.15 7.14 7.10 6.95 6.94 6.88 6.79 6.79 6.69 6.68 4.93 4.72 4.71 4.69 4.51 4.50 4.48 4.05 3.98 3.82 3.68 1.97 1.47 1.46 1.37 1.36 1.35 1.35 0.66
0.90 1.00 4.70 5.37 1.97 1.09 1.08 13.58 6.24 6.56 6.47 2.97
11.0 10.5 10.0 9.5 9.0 8.5 8.0 7.5 7.0 6.5 6.0 5.5 5.0 4.5 4.0 3.5 3.0 2.5 2.0 1.5 1.0 0.5 0.0 -0.5 -1.0 -1.5 -2.0 -2.5 -3.0
ppm

14

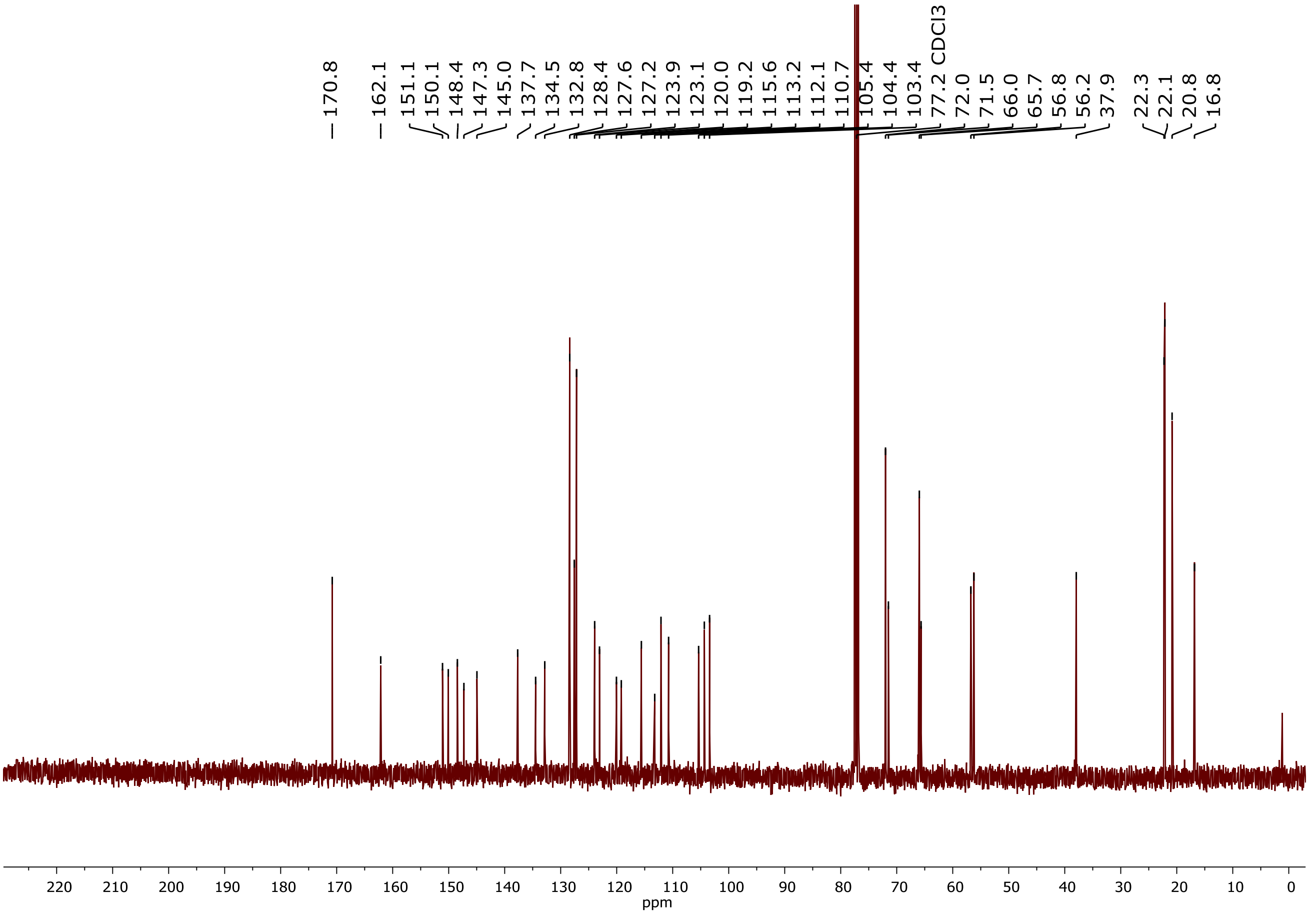
170.8 162.1 151.1 150.1 148.4 147.3 145.0 137.7 134.5 132.8 128.4 127.6 127.2 123.9 123.1 120.0 119.2 115.6 113.2 112.1 110.7 105.4 104.4 103.4 77.2 CDCl3 72.0 71.5 66.0 65.7 56.8 56.2 37.9 22.3 22.1 20.8 16.8
220 210 200 190 180 170 160 150 140 130 120 110 100 90 80 70 60 50 40 30 20 10 0
ppm

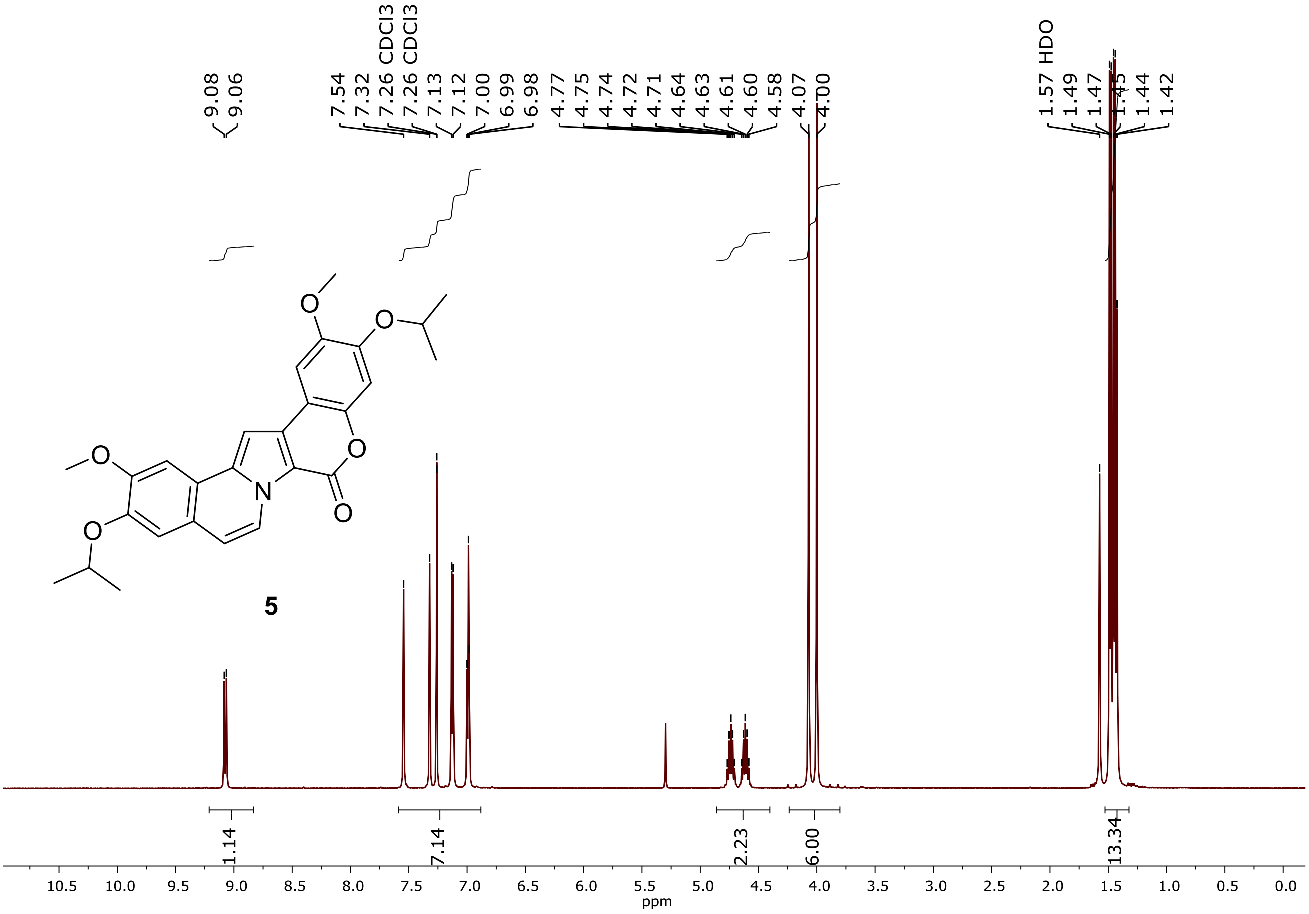
9.08
9.06
7.54
7.32
7.26 CDCl3
7.26 CDCl3
7.13
7.12
7.00
6.99
6.98
4.77
4.75
4.74
4.72
4.71
4.64
4.63
4.61
4.60
4.58
4.07
4.00
1.57 HDO
1.49
1.47
1.45
1.44
1.42
5
1.14
7.14
2.23
6.00
13.34
ppm

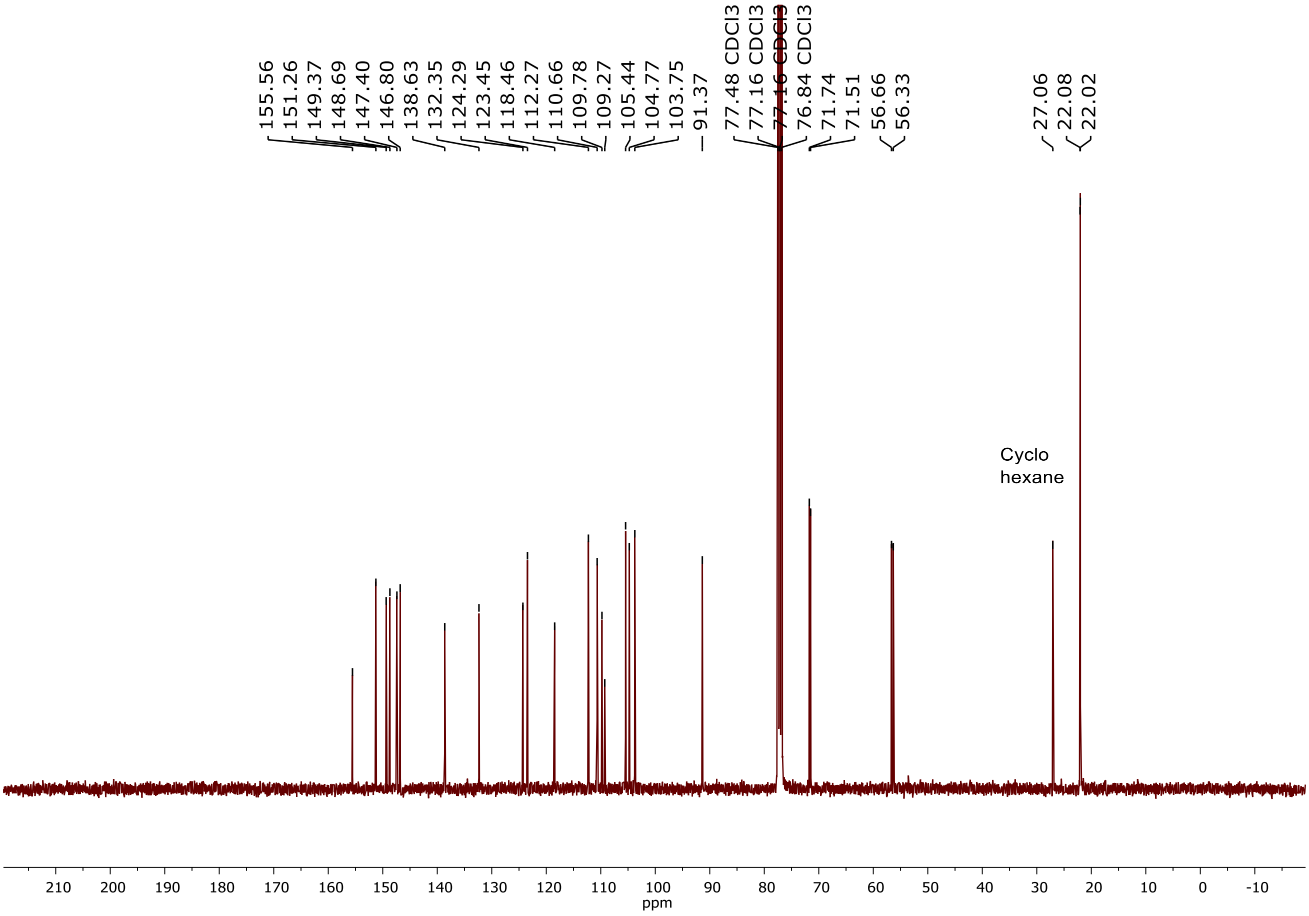
155.56
151.26
149.37
148.69
147.40
146.80
138.63
132.35
124.29
123.45
118.46
112.27
110.66
109.78
109.27
105.44
104.77
103.75
91.37
77.48 CDCl3
77.16 CDCl3
77.16 CDCl3
76.84 CDCl3
71.74
71.51
56.66
56.33
27.06
22.08
22.02
Cyclo
hexane
ppm

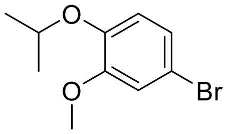

4

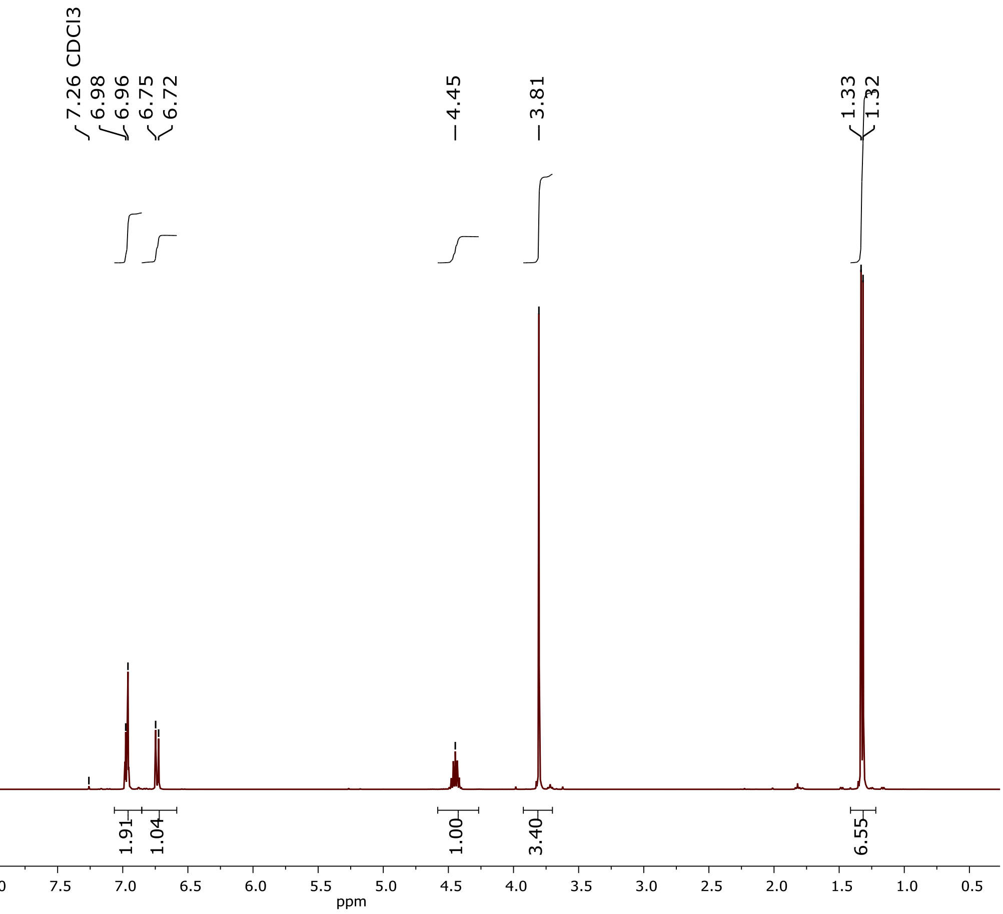

7.26 CDCl3
6.98
6.96
6.75
6.72
4.45
3.81
1.33
1.32
1.91
1.04
1.00
3.40
6.55
10.0 9.5 9.0 8.5 8.0 7.5 7.0 6.5 6.0 5.5 5.0 4.5 4.0 3.5 3.0 2.5 2.0 1.5 1.0 0.5
ppm

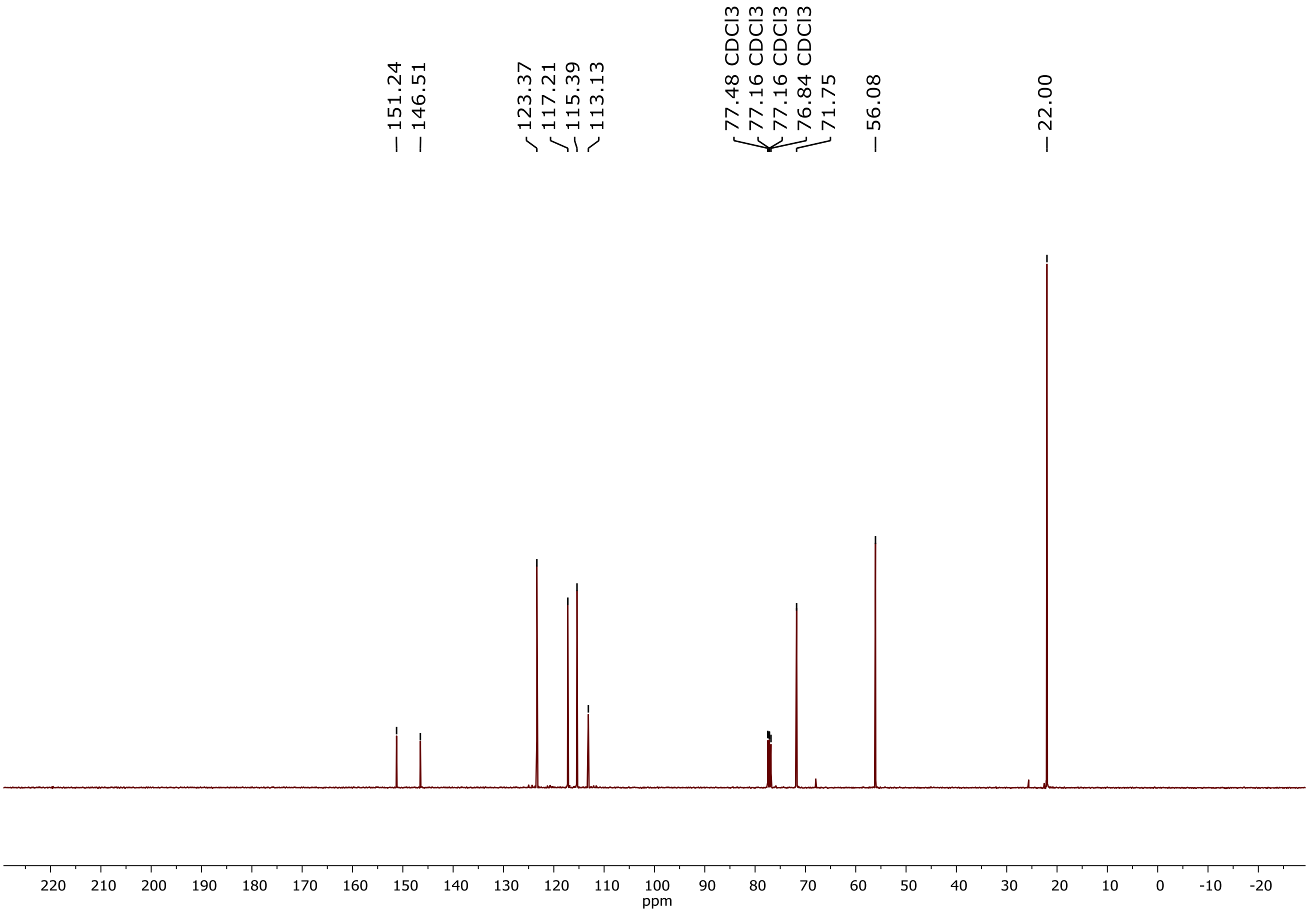

151.24
146.51
123.37
117.21
115.39
113.13
77.48 CDCl3
77.16 CDCl3
77.16 CDCl3
76.84 CDCl3
71.75
56.08
22.00
220 210 200 190 180 170 160 150 140 130 120 110 100 90 80 70 60 50 40 30 20 10 0 -10 -20
ppm

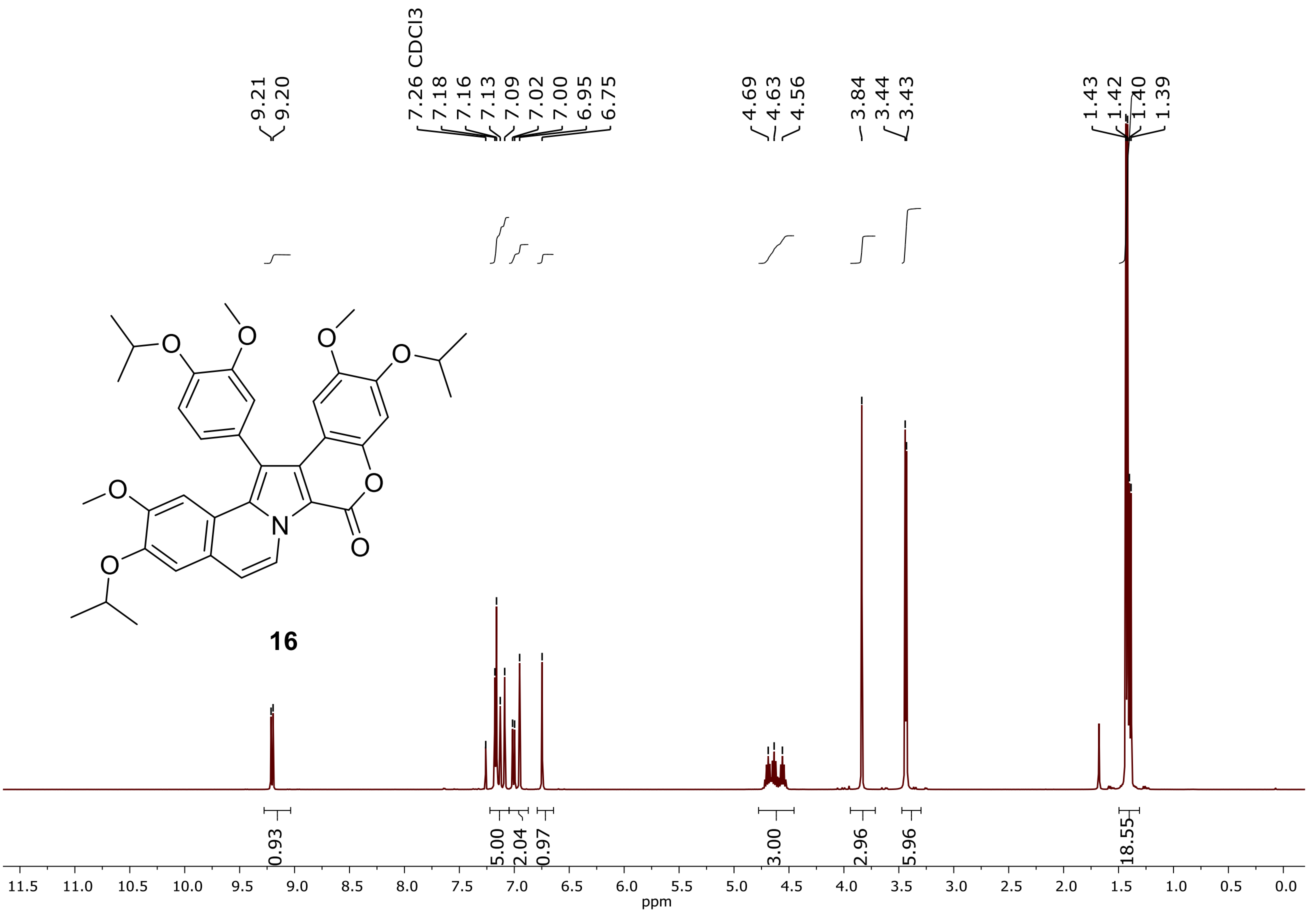

9.21
9.20
7.26 CDCl3
7.18
7.16
7.13
7.09
7.02
7.00
6.95
6.75
4.69
4.63
4.56
3.84
3.44
3.43
1.43
1.42
1.40
1.39
16
0.93
5.00
2.04
0.97
3.00
2.96
5.96
18.55
ppm

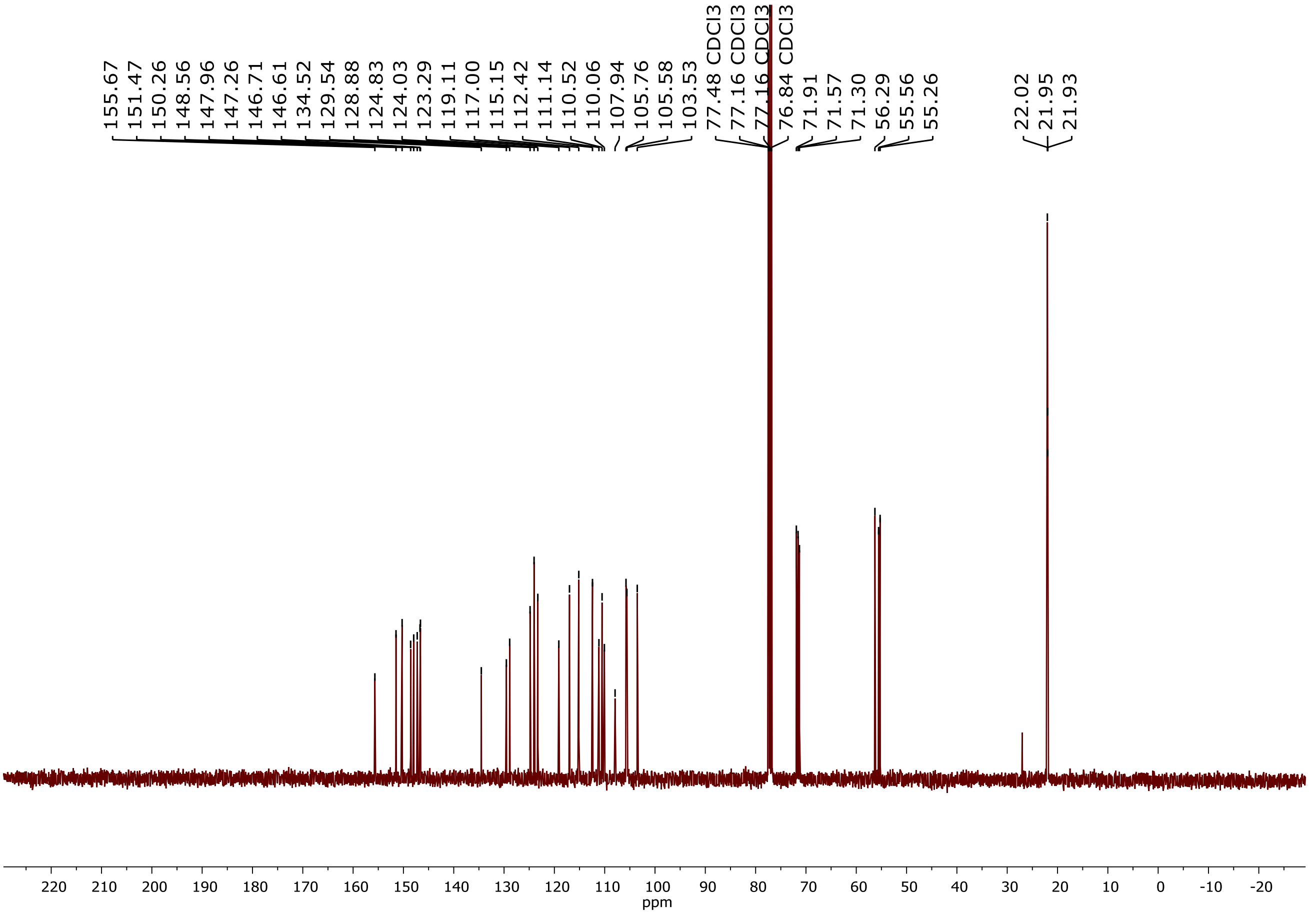

155.67
151.47
150.26
148.56
147.96
147.26
146.71
146.61
134.52
129.54
128.88
124.83
124.03
123.29
119.11
117.00
115.15
112.42
111.14
110.52
110.06
107.94
105.76
105.58
103.53
77.48 CDCl3
77.16 CDCl3
77.16 CDCl3
76.84 CDCl3
71.91
71.57
71.30
56.29
55.56
55.26
22.02
21.95
21.93
ppm

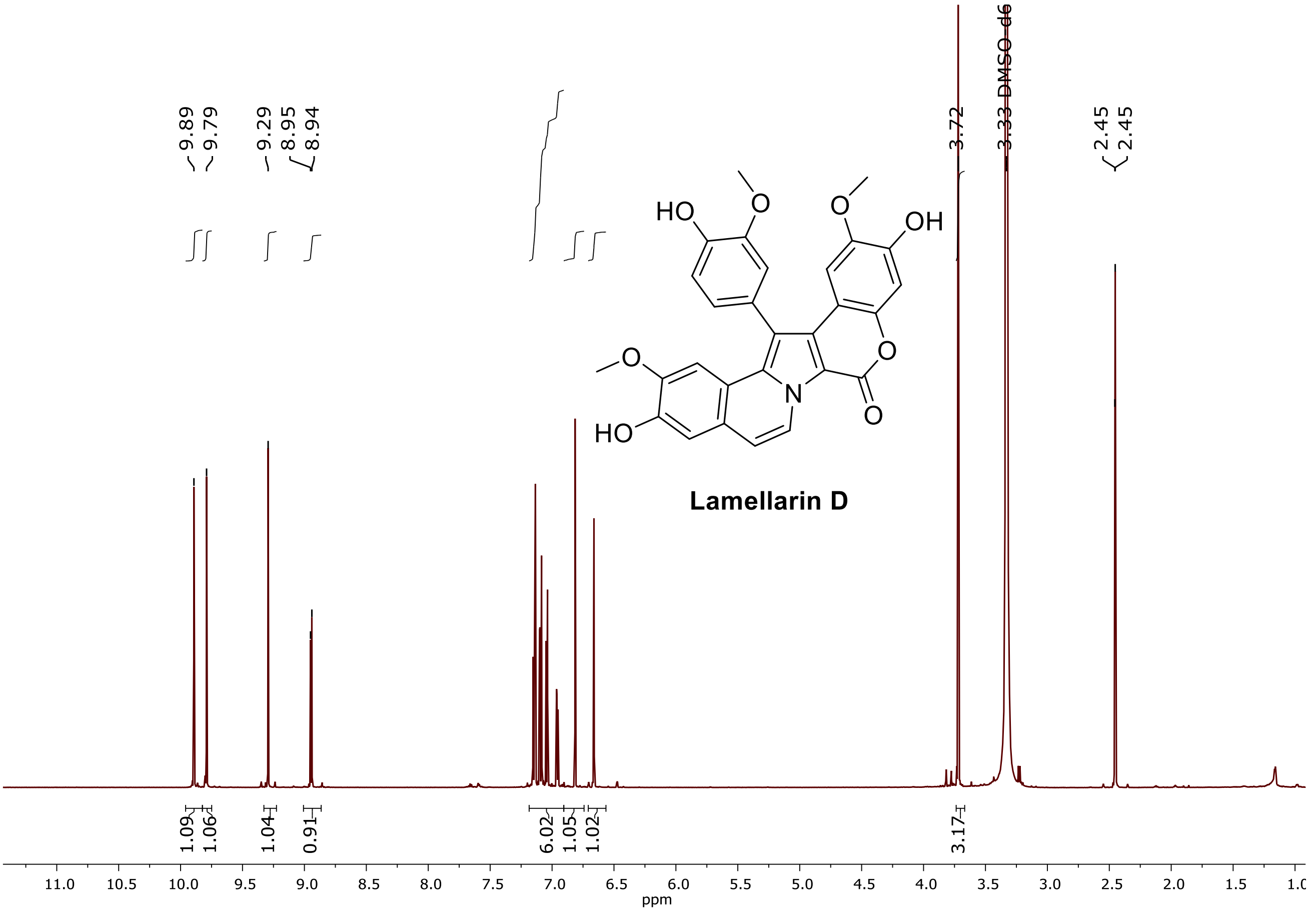
9.89
9.79
9.29
8.95
8.94
3.72
3.33 DMSO-d6
2.45
2.45
1.09
1.06
1.04
0.91
6.02
1.05
1.02
3.17
HO
O
O
OH
O
N
O
HO
O
Lamellarin D
11.0 10.5 10.0 9.5 9.0 8.5 8.0 7.5 7.0 6.5 6.0 5.5 5.0 4.5 4.0 3.5 3.0 2.5 2.0 1.5
ppm

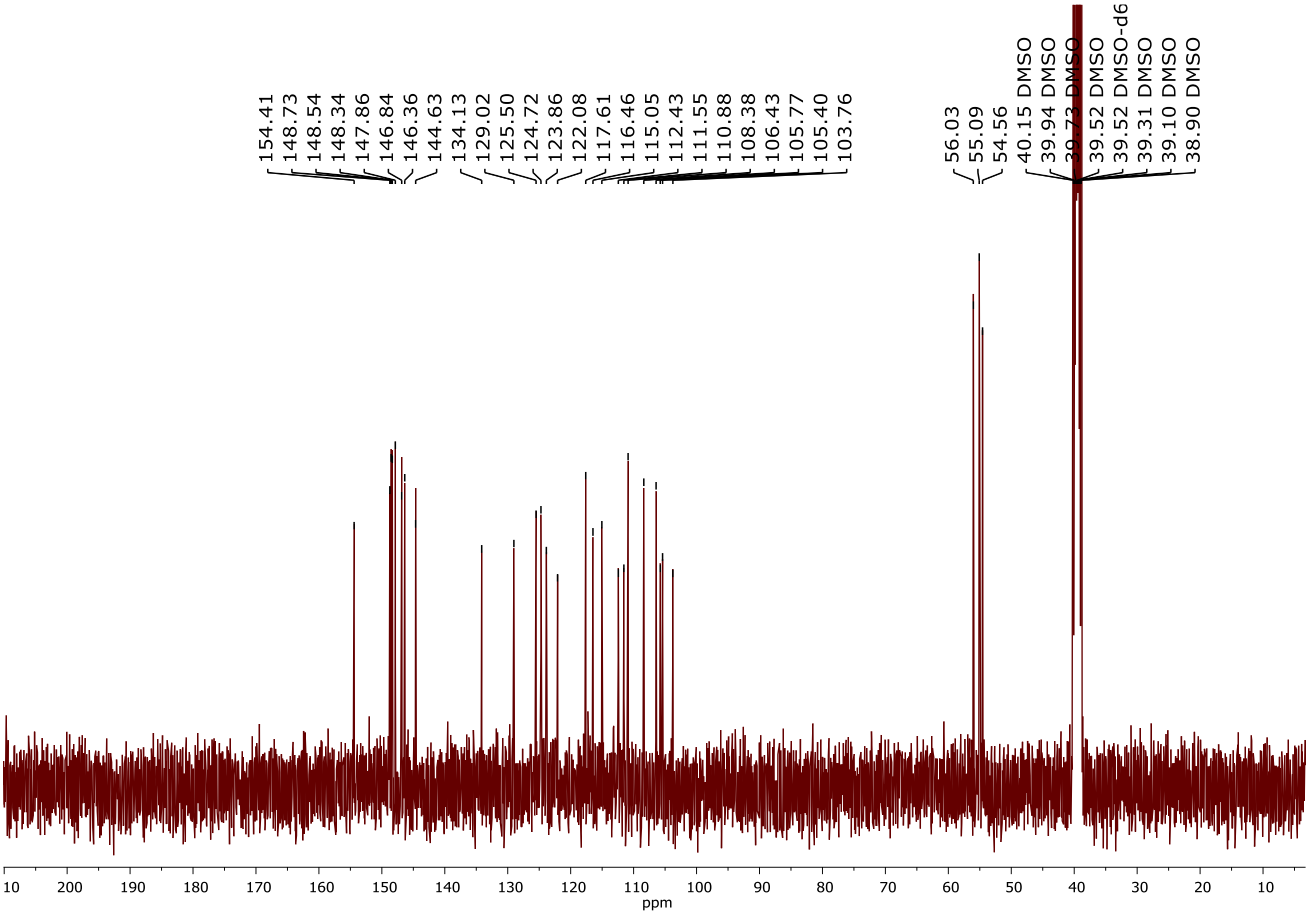
154.41
148.73
148.54
148.34
147.86
146.84
146.36
144.63
134.13
129.02
125.50
124.72
123.86
122.08
117.61
116.46
115.05
112.43
111.55
110.88
108.38
106.43
105.77
105.40
103.76
56.03
55.09
54.56
40.15 DMSO
39.94 DMSO
39.52 DMSO
39.52 DMSO-d6
39.31 DMSO
39.10 DMSO
38.90 DMSO
200 190 180 170 160 150 140 130 120 110 100 90 80 70 60 50 40 30 20 10
ppm

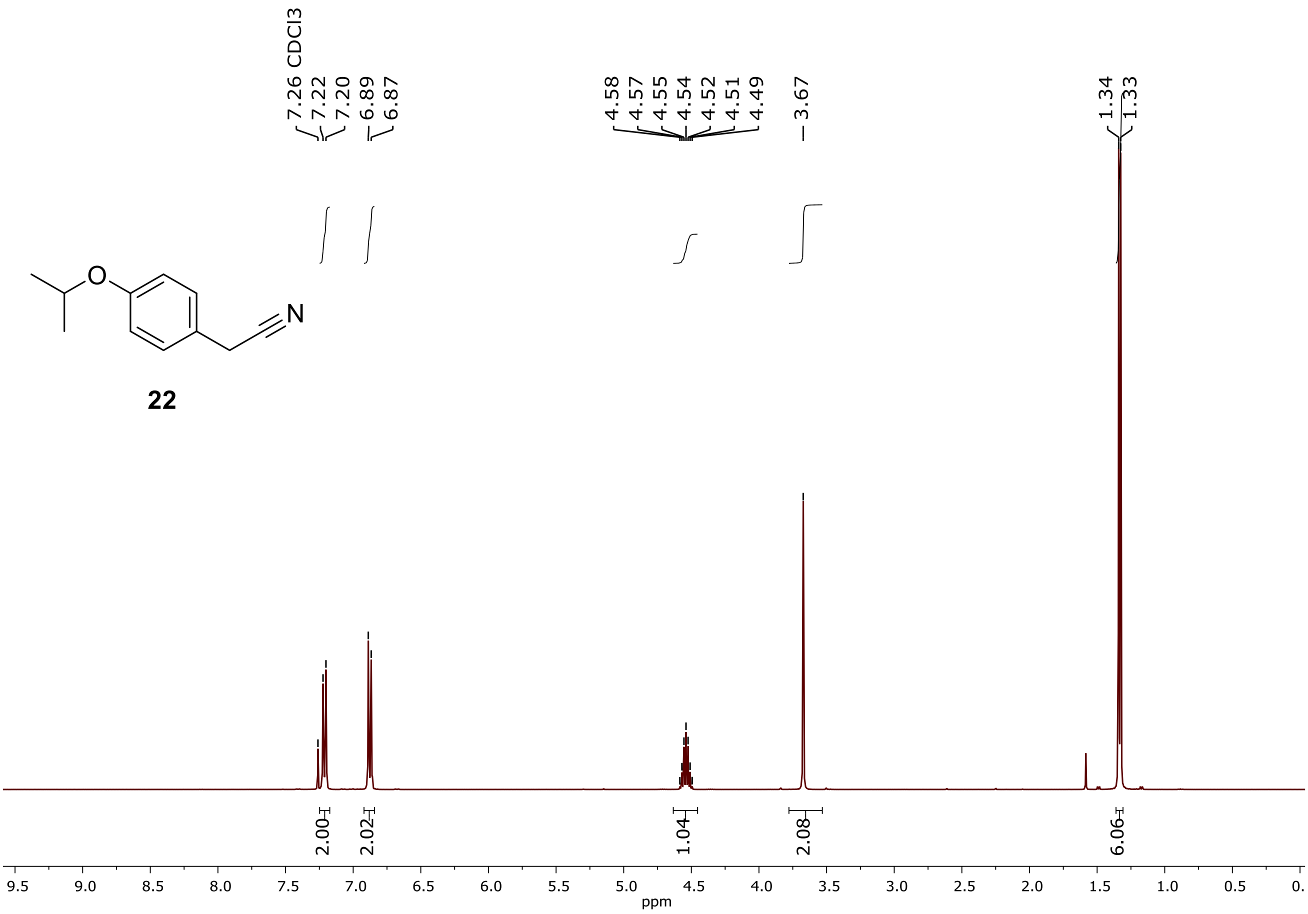
O
N
7.26 CDCl3
7.22
7.20
6.89
6.87
4.58
4.57
4.55
4.54
4.52
4.51
4.49
3.67
1.34
1.33
2.00
2.02
1.04
2.08
6.06
9.5 9.0 8.5 8.0 7.5 7.0 6.5 6.0 5.5 5.0 4.5 4.0 3.5 3.0 2.5 2.0 1.5 1.0 0.5 0.
ppm

22

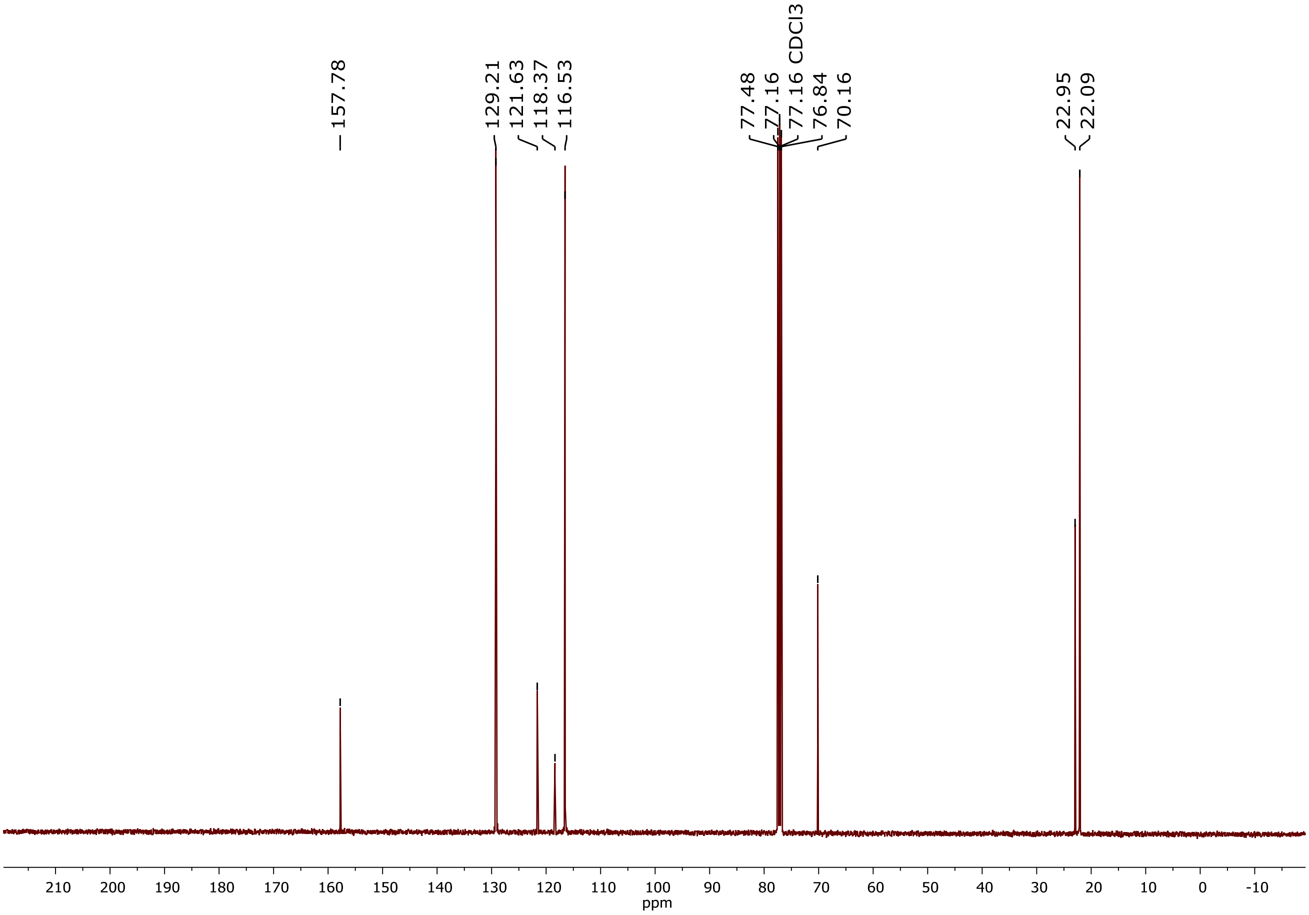
157.78
129.21
121.63
118.37
116.53
77.48
77.16
77.16 CDCl3
76.84
70.16
22.95
22.09
210 200 190 180 170 160 150 140 130 120 110 100 90 80 70 60 50 40 30 20 10 0 -10
ppm

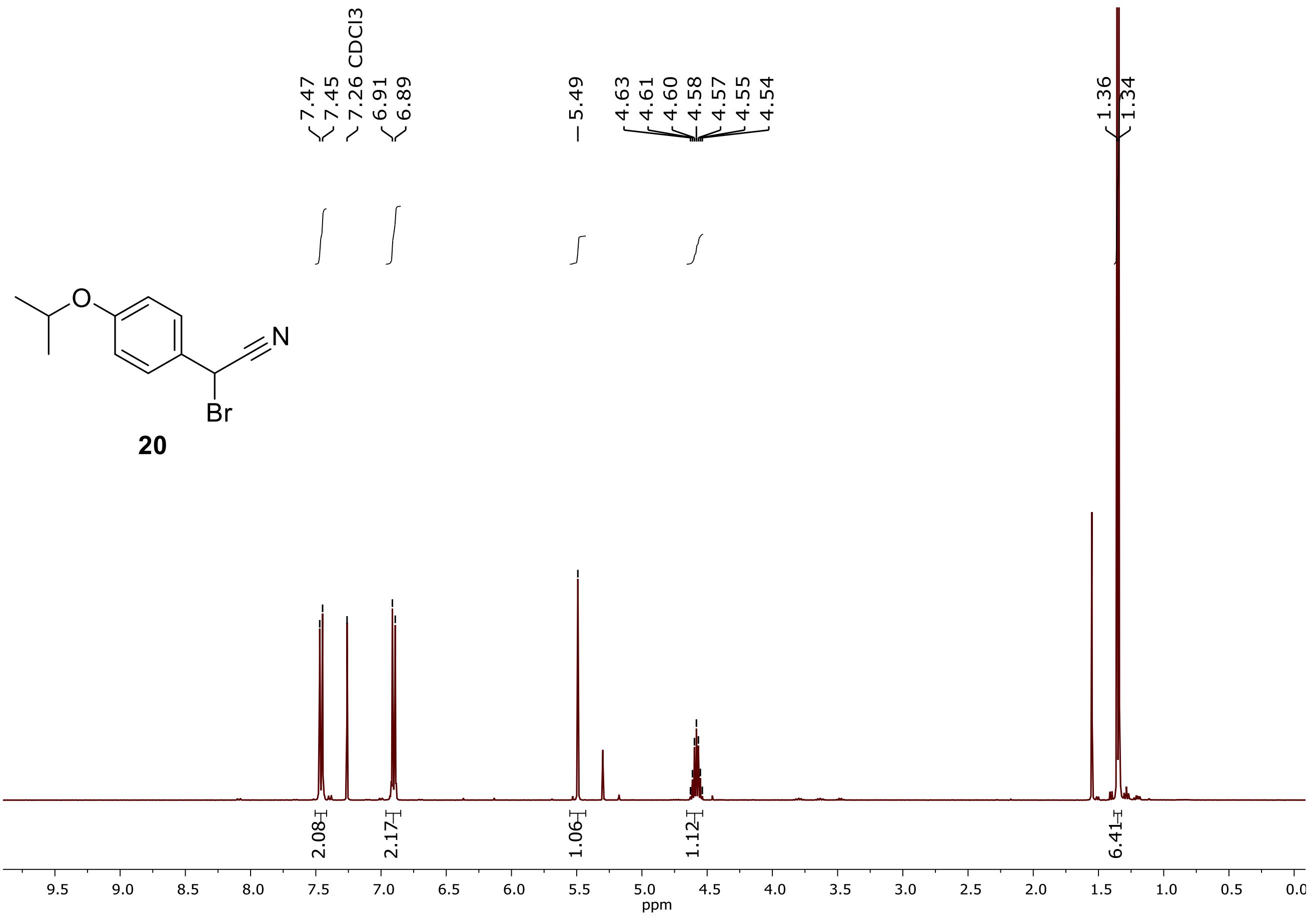
O
N
Br
20
7.47
7.45
7.26 CDCl3
6.91
6.89
5.49
4.63
4.61
4.60
4.58
4.57
4.55
4.54
1.36
1.34
2.08
2.17
1.06
1.12
6.41
9.5 9.0 8.5 8.0 7.5 7.0 6.5 6.0 5.5 5.0 4.5 4.0 3.5 3.0 2.5 2.0 1.5 1.0 0.5
ppm

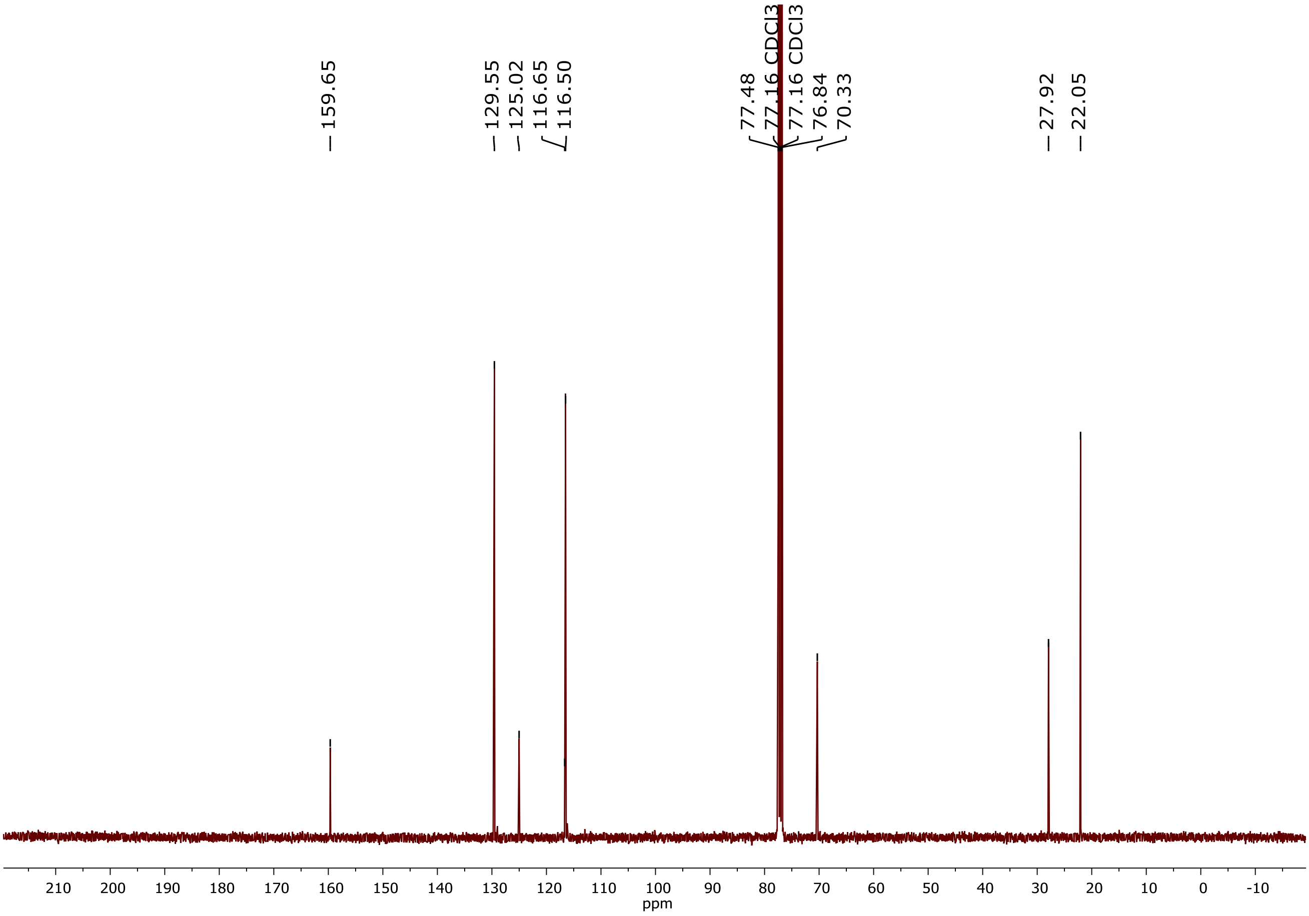
159.65
129.55
125.02
116.65
116.50
77.48
77.16 CDCl3
77.16 CDCl3
76.84
70.33
27.92
22.05
210 200 190 180 170 160 150 140 130 120 110 100 90 80 70 60 50 40 30 20 10 0 -10
ppm

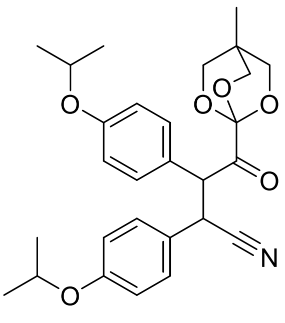

18

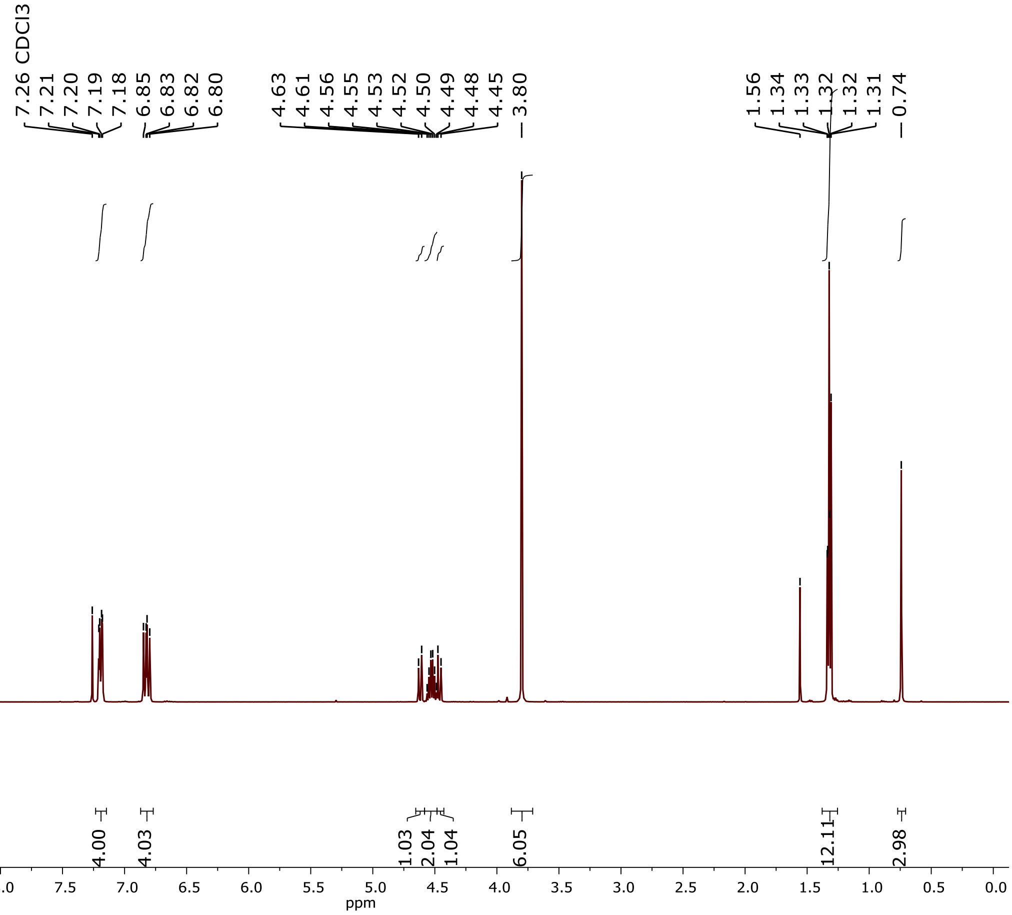
7.26 CDCl3
7.21
7.20
7.19
7.18
6.85
6.83
6.82
6.80
4.63
4.61
4.56
4.55
4.53
4.52
4.50
4.49
4.48
4.45
3.80
1.56
1.34
1.33
1.32
1.32
1.31
0.74
4.00
4.03
1.03
2.04
1.04
6.05
12.11
2.98
10.0 9.5 9.0 8.5 8.0 7.5 7.0 6.5 6.0 5.5 5.0 4.5 4.0 3.5 3.0 2.5 2.0 1.5 1.0 0.5 0.0
ppm

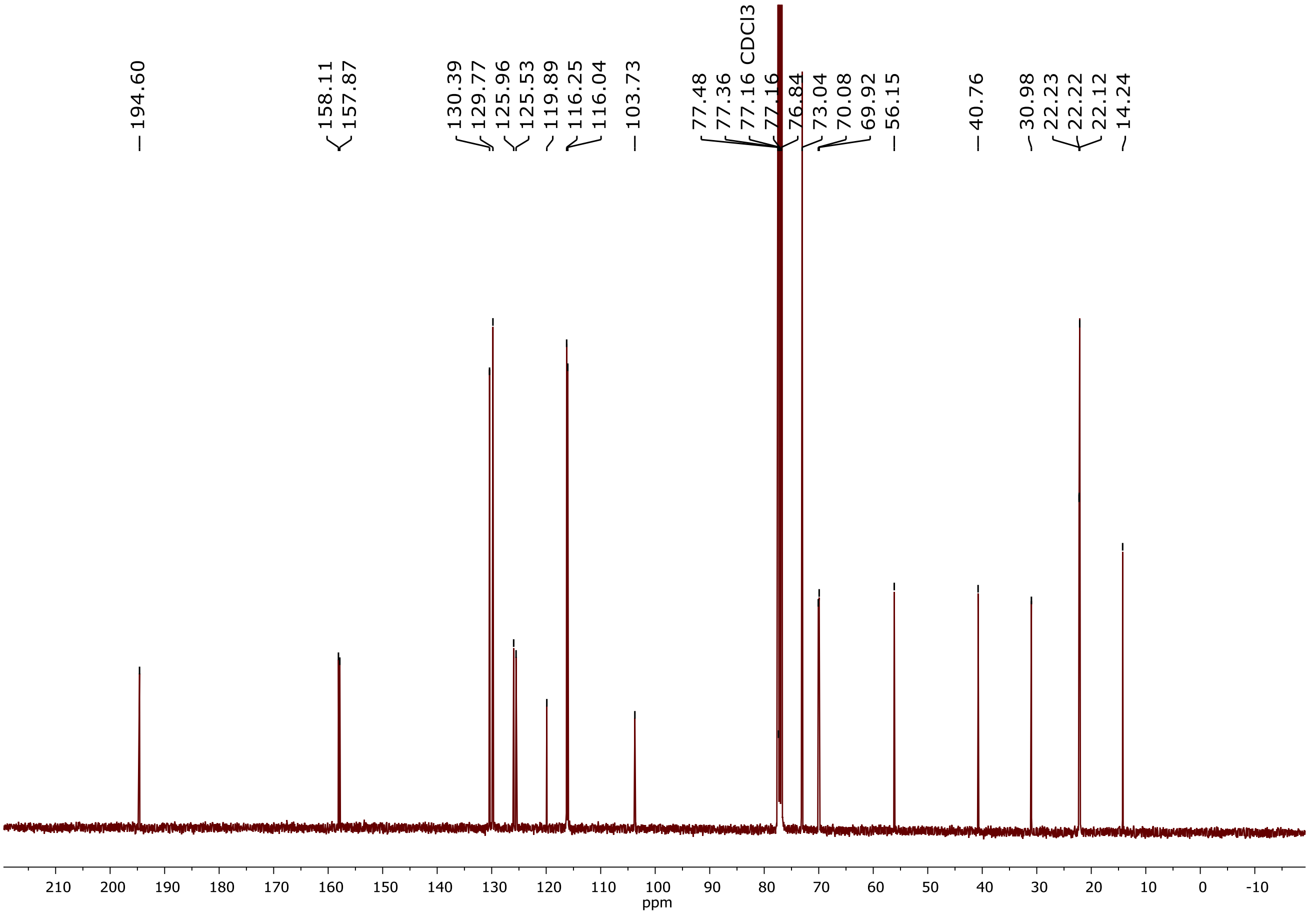
194.60
158.11
157.87
130.39
129.77
125.96
125.53
119.89
116.25
116.04
103.73
77.48
77.36
77.16 CDCl3
77.16
76.84
73.04
70.08
69.92
56.15
40.76
30.98
22.23
22.22
22.12
14.24
210 200 190 180 170 160 150 140 130 120 110 100 90 80 70 60 50 40 30 20 10 0 -10
ppm

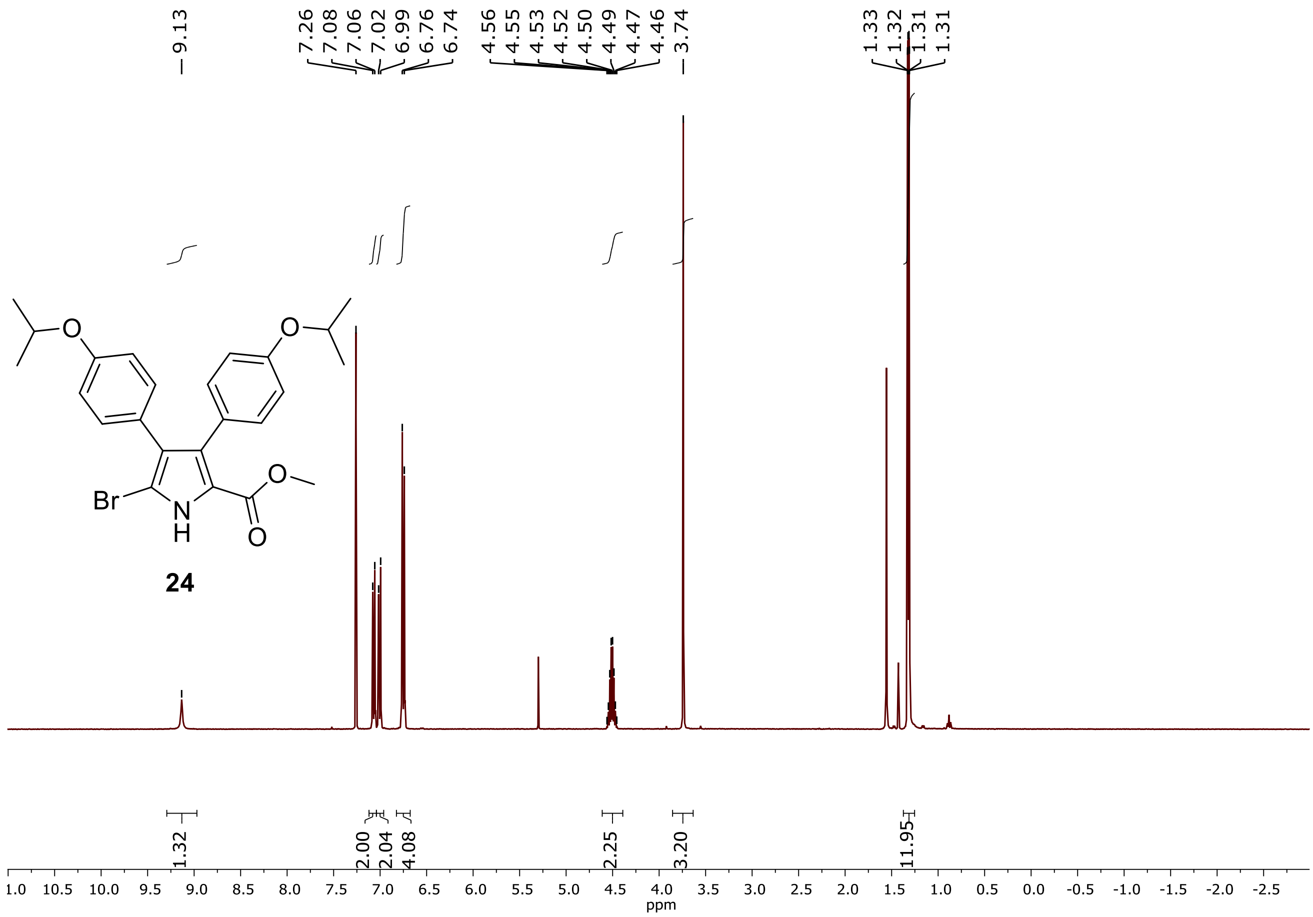
9.13
7.26
7.08
7.06
7.02
6.99
6.76
6.74
4.56
4.55
4.53
4.52
4.50
4.49
4.47
4.46
3.74
1.33
1.32
1.31
1.31
O
O
Br
N
H
O
O
24
1.32
2.00
2.04
4.08
2.25
3.20
11.95
1.0 10.5 10.0 9.5 9.0 8.5 8.0 7.5 7.0 6.5 6.0 5.5 5.0 4.5 4.0 3.5 3.0 2.5 2.0 1.5 1.0 0.5 0.0 -0.5 -1.0 -1.5 -2.0 -2.5
ppm

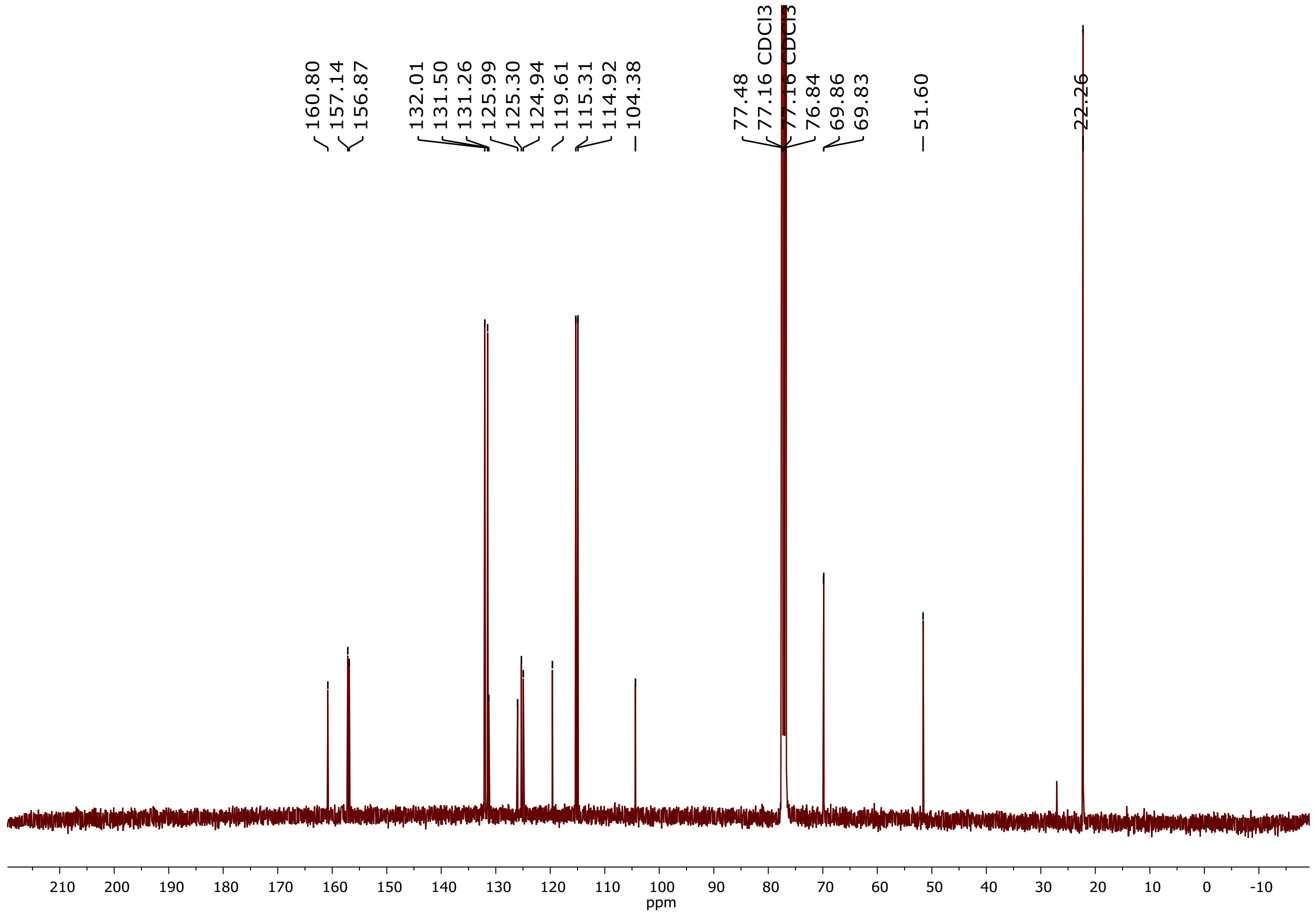
160.80
157.14
156.87
132.01
131.50
131.26
125.99
125.30
124.94
119.61
115.31
114.92
104.38
77.48
77.16 CDCl3
77.16 CDCl3
76.84
69.86
69.83
51.60
22.26
210 200 190 180 170 160 150 140 130 120 110 100 90 80 70 60 50 40 30 20 10 0 -10
ppm

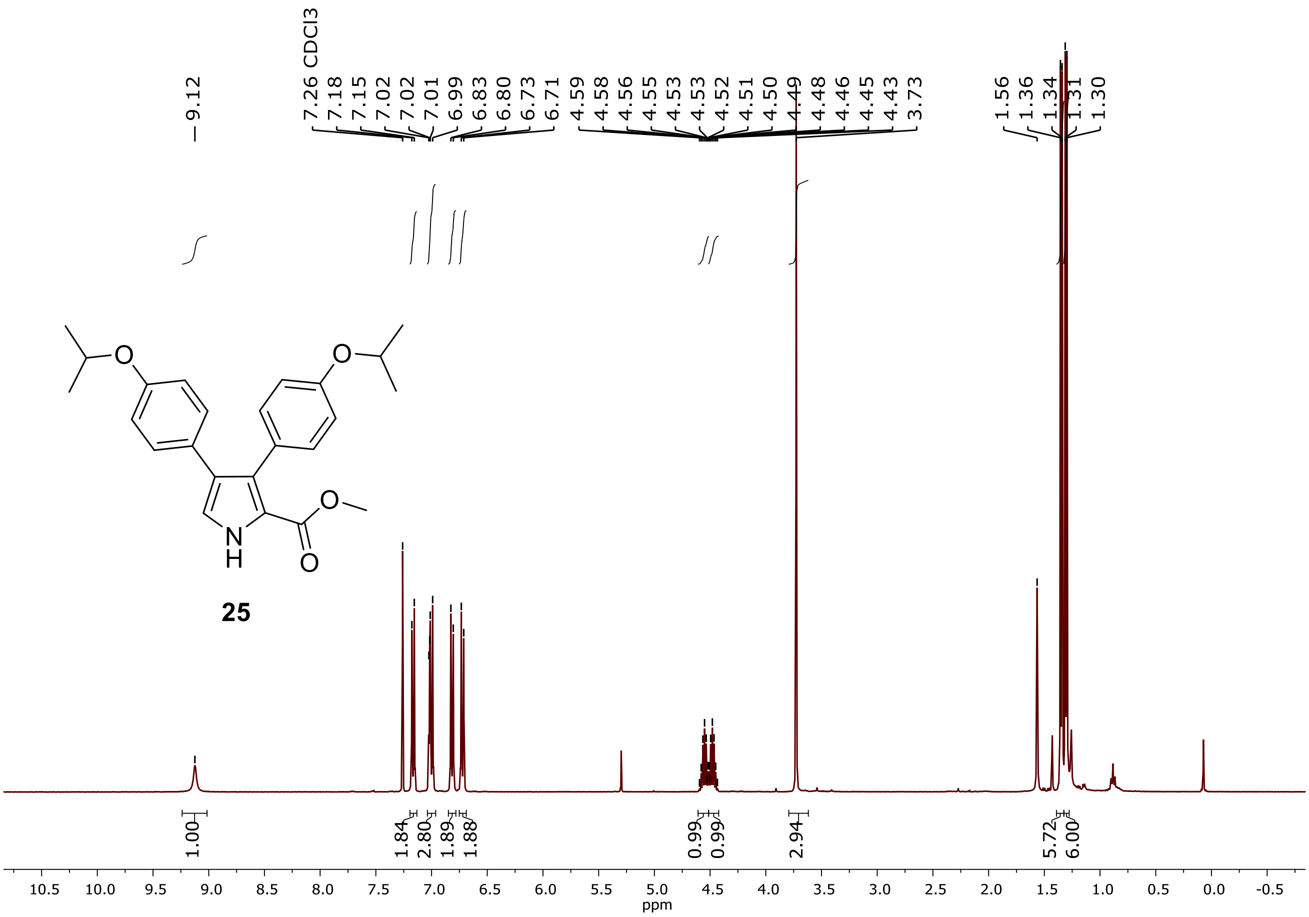
9.12
7.26 CDCl3
7.18
7.15
7.02
7.02
7.01
6.99
6.83
6.80
6.73
6.71
4.59
4.58
4.56
4.55
4.53
4.53
4.52
4.51
4.50
4.49
4.48
4.46
4.45
4.43
3.73
1.56
1.36
1.34
1.31
1.30
O
O
O
O
N
H
25
1.00
1.84
2.80
1.89
1.88
0.99
0.99
2.94
5.72
6.00
10.5 10.0 9.5 9.0 8.5 8.0 7.5 7.0 6.5 6.0 5.5 5.0 4.5 4.0 3.5 3.0 2.5 2.0 1.5 1.0 0.5 0.0 -0.5
ppm

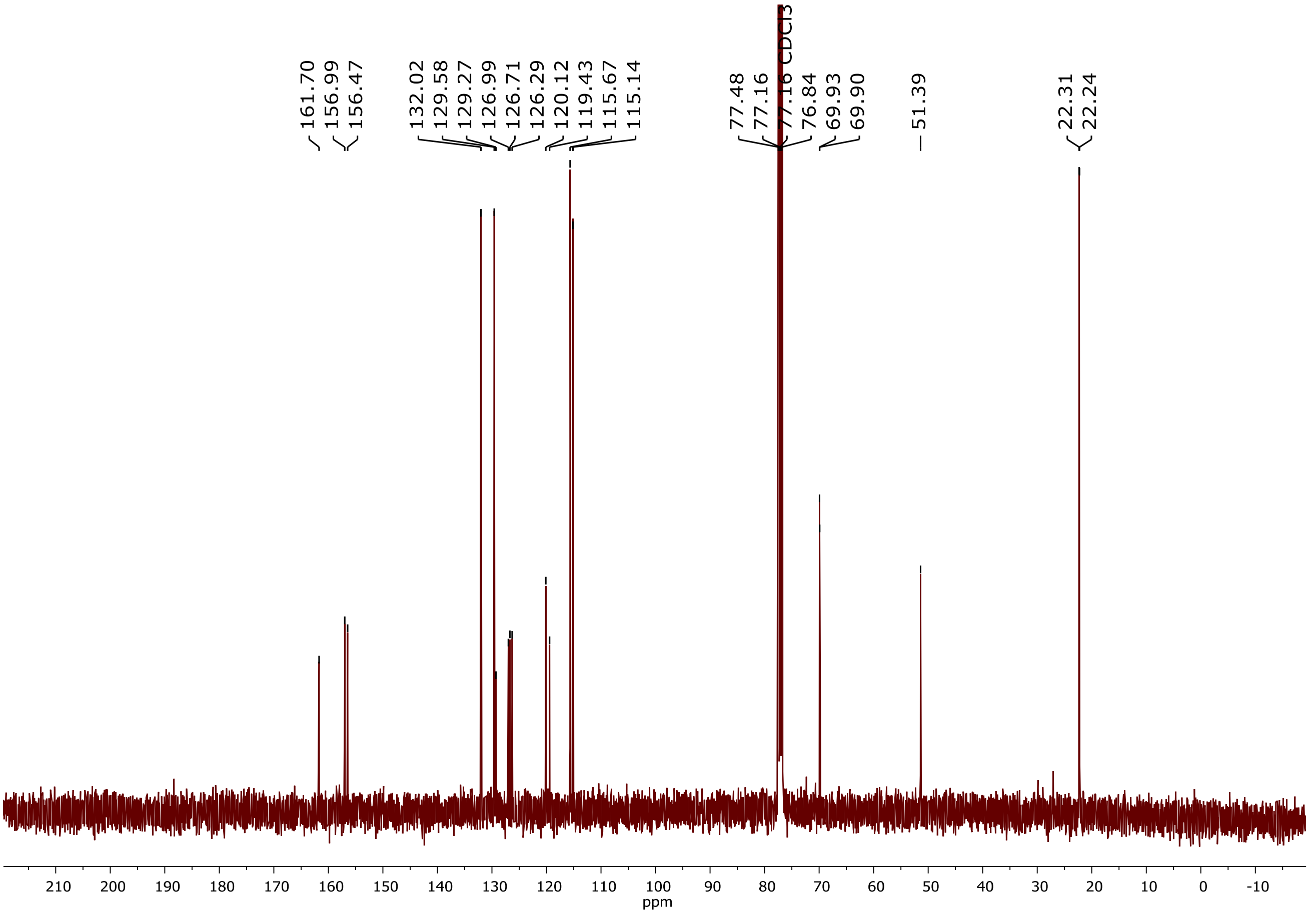
161.70
156.99
156.47
132.02
129.58
129.27
126.99
126.71
126.29
120.12
119.43
115.67
115.14
77.48
77.16
77.16 CDCl3
76.84
69.93
69.90
51.39
22.31
22.24
210 200 190 180 170 160 150 140 130 120 110 100 90 80 70 60 50 40 30 20 10 0 -10
ppm

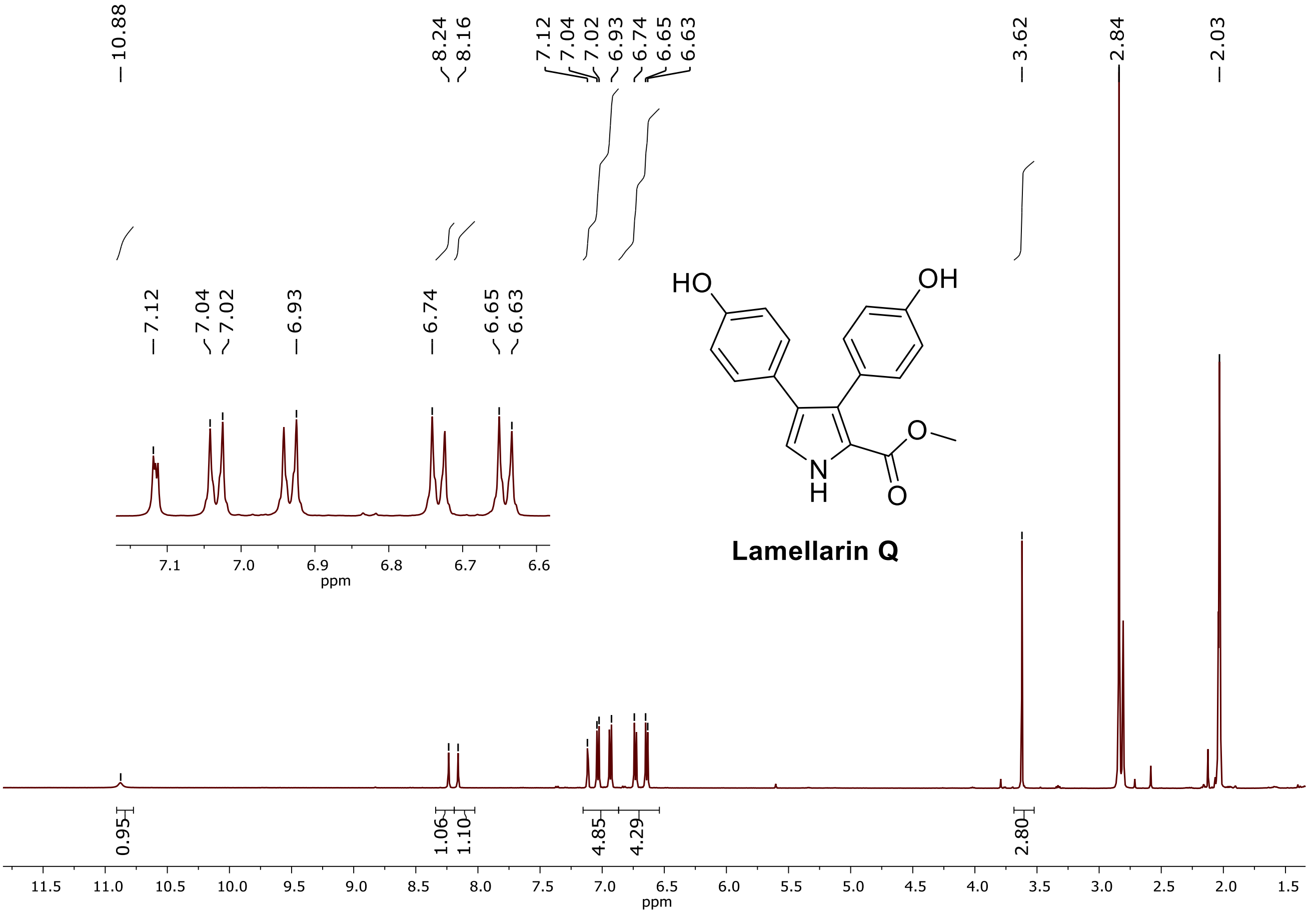
10.88
8.24
8.16
7.12
7.04
7.02
6.93
6.74
6.65
6.63
3.62
2.84
2.03
HO
OH
O
N
H
O
Lamellarin Q
0.95
1.06
1.10
4.85
4.29
2.80
ppm

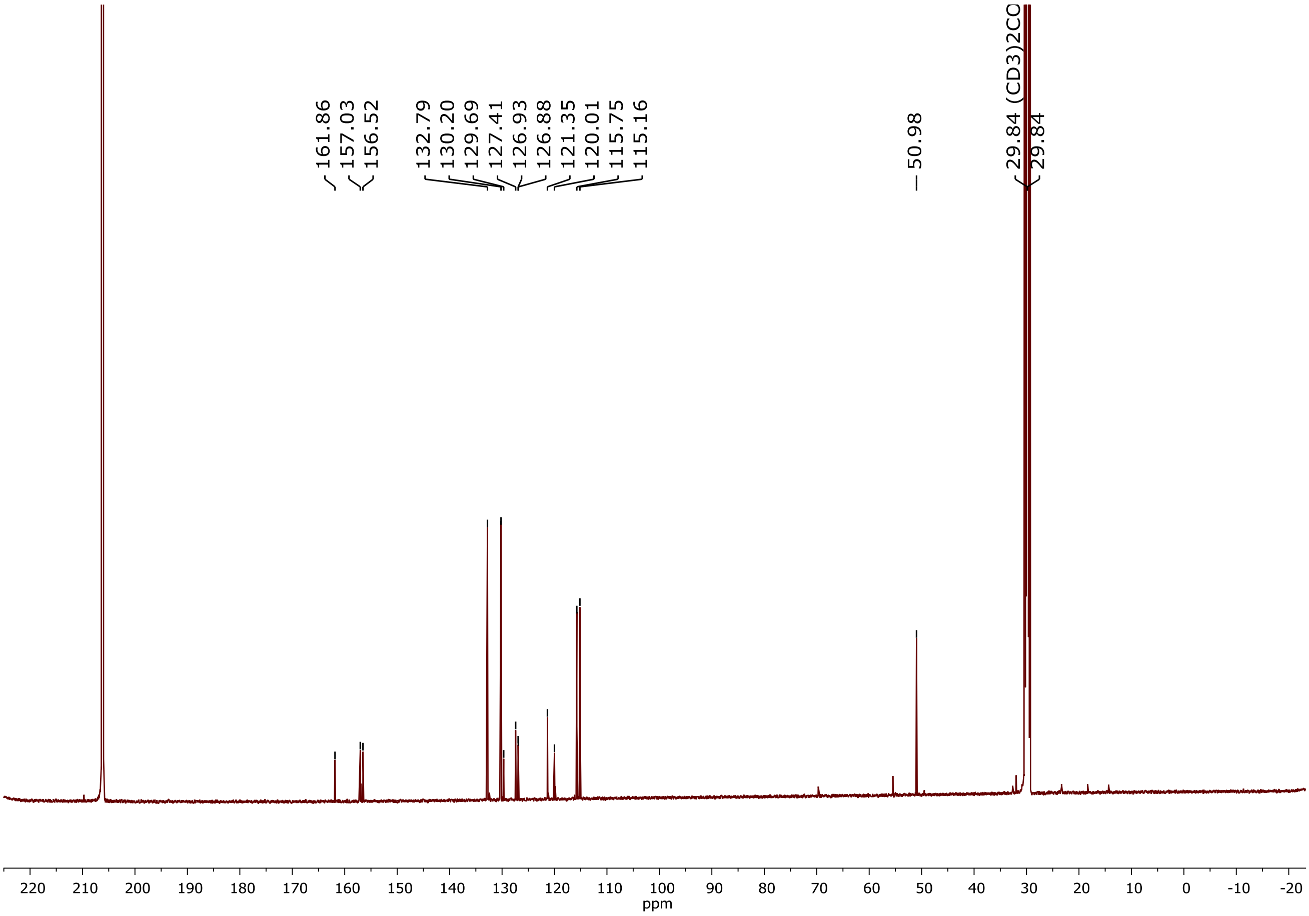
161.86
157.03
156.52
132.79
130.20
129.69
127.41
126.93
126.88
121.35
120.01
115.75
115.16
50.98
29.84 (CD3)2CO
29.84
ppm
